# Supplementary material for: Investigating Italian disinformation spreading on Twitter in the context of 2019 European elections
Source: PLoS One. 2020 Jan 17;15(1):e0227821. doi: 10.1371/journal.pone.0227821 (PMC6968875; doi:10.1371/journal.pone.0227821)
Supplement: Attachment — Submitted filename: paper.pdf [file pone.0227821.s001.pdf]

The paper discusses some results related to the spreading of false news in the period preceding the European elections. Overall, the paper is interesting, clear and relatively sound in the methods. I recommend the paper for publication after some minor issues will be fixed.

- I think the authors should introduce a bit better the context of European elections as well as the major concerns in the public debate regarding such an event. This would provide a better contextualization to the paper.
- Line 28: "As more people is more and more suspicious towards traditional media coverage". This statement should be supported by some empirical evidence (facts) or by some citations.
- Line 48: "trust in news is today particularly low (40% in general, 23% on social media)". What these percentages refer to?
- Line 134: "We can observe that, in general, articles devoted to debunk false claims were barely engaged, accounting only for 6% of the total volume of tweets spreading disinformation 135 in the same period;". This is expected also considering the very low effectiveness of debunking strategies (see Zollo, F., Bessi, A., Del Vicario, M., Scala, A., Caldarelli, G., Shekhtman, L., ... & Quattrociocchi, W. (2017). Debunking in a world of tribes. PloS one, 12(7), e0181821).
- Legend of Figure 2 is too small.
- Line 155: Authors use as subsection name "Building the re-tweeting network". However, as far as I understood, their network doesn't comprehend re-tweets only. About this aspect: the authors aggregate different kind of interactions that sometimes are treated separately. Indeed the re-tweets can be associated to an endorsement procedure while mentions etc.. can be associated to a communication procedure. The fact of aggregating is not necessarily wrong, however the authors should discuss the different possibilities of treating their data.
- Line 222-226: this paragraph results hard to follow. I suggest to the authors to spend few more lines in explaining the ratio behind the FWER.

- Figure 3: In the caption the author say "total number of shared articles" but the axis annotation says "published". I see that the two words can be used interchangeably but in the context of social media where "sharing" and "publishing" refer to a precise actions I would suggest to not interchange the two words.
- Line 285: The authors divide users in different classes based on their activity. How the partitioning that they implement resembles the distribution of users' activity? On what principles is their partitioning based?
- Lines 301-315: The authors discuss a series of findings that are not supported by Figures or other material.
- Figure 5: Pie charts are well known for being bad tools for summarizing data. Maybe a stacked bar chart would be better.
- Line 342: Are the titles somehow pre-processed? All the words mentioned by the authors are nouns so I assume that POS tagging has been performed. Additionally, the list of keywords provided by the volunteer should be better specified. How many words did they provide per topic? Afterwards they say that one of the most frequent keyword is "video". So I assume that volunteers provided the list of keywords while looking at the data rather than just providing words "of interest" during the period of european elections. Also the topics that "emerge" from the data are sometimes referred as categories. Summarizing I think that this part of the paper should be written in a more clear and precise way.
- Line 356: "proving that a remarkable fraction of disinformation was shared as multimedia content". Reference/s needed.
- Line 427: "Despite centrality measures do not generally agree in their ranking". However, centrality measures (degree, eigenvector, closeness) normally display strong correlations among each other so they may display small differences in the ranking but overall display a good agreement. (see for instance, Valente, T. W., Coronges, K., Lakon, C., & Costenbader, E. (2008). How correlated are network centrality measures?. Connections (Toronto, Ont.), 28(1), 16., among others)

- Line 495: ”measures” is used instead of ”measure”
