# Supplementary material for: Investigating Italian disinformation spreading on Twitter in the context of 2019 European elections
Source: PLoS One. 2020 Jan 17;15(1):e0227821. doi: 10.1371/journal.pone.0227821 (PMC6968875; doi:10.1371/journal.pone.0227821)
Supplement: Attachment — Submitted filename: PONE-D-19-20362_R1_reviewer.pdf [file pone.0227821.s004.pdf]

PLOS ONE

Investigating Italian disinformation spreading on Twitter in the context of 2019 European elections

--Manuscript Draft--

**Manuscript Number:**	PONE-D-19-20362R1
**Article Type:**	Research Article
**Full Title:**	Investigating Italian disinformation spreading on Twitter in the context of 2019 European elections
**Short Title:**	Italian disinformation on Twitter in 2019 European elections
**Corresponding Author:**	Francesco Pierri<br>Politecnico di Milano - Milano Leonardo<br>Milano, ITALY
**Keywords:**	computational social science; Twitter; Social networks; disinformation; European Parliament elections
**Abstract:**	We investigate the presence (and the influence) of disinformation spreading on online social networks in Italy, in the 5-month period preceding the 2019 European Parliament elections. To this aim we collected a large-scale dataset of tweets associated to thousands of news articles published on Italian disinformation websites. In the observation period, a few outlets accounted for most of the deceptive information circulating on Twitter, which focused on controversial and polarizing topics of debate such as immigration, national safety and (Italian) nationalism. We found evidence of connections between different disinformation outlets across Europe, U.S. and Russia, which often linked to each other and featured similar, even translated, articles in the period before the elections. Overall, the spread of disinformation on Twitter was confined in a limited community, strongly (and explicitly) related to the Italian conservative and far-right political environment, who had a limited impact on online discussions on the up-coming elections.
**Order of Authors:**	Francesco Pierri
	Alessandro Artoni
	Stefano Ceri
**Response to Reviewers:**	(see attached file "response_reviews_final.docx" for a better view of comments/answers)
**Additional Information:**	
**Question**	**Response**
**Financial Disclosure**<br><br>Enter a financial disclosure statement that describes the sources of funding for the work included in this submission. Review the submission guidelines for detailed requirements. View published research articles from *PLOS ONE* for specific examples.<br><br>This statement is required for submission and **will appear in the published article** if the submission is accepted. Please make sure it is accurate.	F.P. and S.C. are supported by the PRIN grant HOPE (FP6, Italian Ministry of Education). S.C. is partially supported by ERC Advanced Grant 693174.<br>The funders had no role in study design, data collection and analysis, decision to publish, or preparation of the manuscript.

Powered by Editorial Manager® and ProduXion Manager® from Aries Systems Corporation

**Unfunded studies**<br>Enter: *The author(s) received no specific funding for this work.*<br><br>**Funded studies**<br>Enter a statement with the following details:<br>• Initials of the authors who received each award<br>• Grant numbers awarded to each author<br>• The full name of each funder<br>• URL of each funder website<br>• Did the sponsors or funders play any role in the study design, data collection and analysis, decision to publish, or preparation of the manuscript?<br>• **NO** - Include this sentence at the end of your statement: *The funders had no role in study design, data collection and analysis, decision to publish, or preparation of the manuscript.*<br>• **YES** - Specify the role(s) played.<br><br>* typeset	
**Competing Interests**<br><br>Use the instructions below to enter a competing interest statement for this submission. On behalf of all authors, disclose any competing interests that could be perceived to bias this work—acknowledging all financial support and any other relevant financial or non-financial competing interests.<br><br>This statement **will appear in the published article** if the submission is accepted. Please make sure it is accurate. View published research articles from *PLOS ONE* for specific examples.	The authors have declared that no competing interests exist.

**NO authors have competing interests**<br><br>Enter: *The authors have declared that no competing interests exist.*<br><br>**Authors with competing interests**<br><br>Enter competing interest details beginning with this statement:<br><br>*I have read the journal's policy and the authors of this manuscript have the following competing interests: [insert competing interests here]*<br><br>* typeset	
**Ethics Statement**<br><br>Enter an ethics statement for this submission. This statement is required if the study involved:<br><br>• Human participants<br>• Human specimens or tissue<br>• Vertebrate animals or cephalopods<br>• Vertebrate embryos or tissues<br>• Field research<br><br>Write "N/A" if the submission does not require an ethics statement.<br><br>General guidance is provided below. Consult the submission guidelines for detailed instructions. **Make sure that all information entered here is included in the Methods section of the manuscript.**	N/A

**Format for specific study types**<br><br>**Human Subject Research (involving human participants and/or tissue)**<br>• Give the name of the institutional review board or ethics committee that approved the study<br>• Include the approval number and/or a statement indicating approval of this research<br>• Indicate the form of consent obtained (written/oral) or the reason that consent was not obtained (e.g. the data were analyzed anonymously)<br><br>**Animal Research (involving vertebrate animals, embryos or tissues)**<br>• Provide the name of the Institutional Animal Care and Use Committee (IACUC) or other relevant ethics board that reviewed the study protocol, and indicate whether they approved this research or granted a formal waiver of ethical approval<br>• Include an approval number if one was obtained<br>• If the study involved *non-human primates*, add *additional details* about animal welfare and steps taken to ameliorate suffering<br>• If anesthesia, euthanasia, or any kind of animal sacrifice is part of the study, include briefly which substances and/or methods were applied<br><br>**Field Research**<br><br>Include the following details if this study involves the collection of plant, animal, or other materials from a natural setting:<br>• Field permit number<br>• Name of the institution or relevant body that granted permission	
**Data Availability**<br><br>Authors are required to make all data underlying the findings described fully available, without restriction, and from the time of publication. PLOS allows rare exceptions to address legal and ethical concerns. See the PLOS Data Policy and FAQ for detailed information.	Yes - all data are fully available without restriction

A Data Availability Statement describing where the data can be found is required at submission. Your answers to this question constitute the Data Availability Statement and **will be published in the article**, if accepted.<br><br>**Important:** Stating 'data available on request from the author' is not sufficient. If your data are only available upon request, select 'No' for the first question and explain your exceptional situation in the text box.<br><br>Do the authors confirm that all data underlying the findings described in their manuscript are fully available without restriction?	
**Describe where the data may be found in full sentences. If you are copying our sample text, replace any instances of XXX with the appropriate details.**<br><br>• If the data are **held or will be held in a public repository**, include URLs, accession numbers or DOIs. If this information will only be available after acceptance, indicate this by ticking the box below. For example: *All XXX files are available from the XXX database (accession number(s) XXX, XXX.*).<br>• If the data are all contained **within the manuscript and/or Supporting Information files**, enter the following: *All relevant data are within the manuscript and its Supporting Information files.*<br>• If neither of these applies but you are able to provide **details of access elsewhere**, with or without limitations, please do so. For example:<br><br>*Data cannot be shared publicly because of [XXX]. Data are available from the XXX Institutional Data Access / Ethics Committee (contact via XXX) for researchers who meet the criteria for access to confidential data.*<br><br>*The data underlying the results presented in the study are available from (include the name of the third party*	Data is publicly accessible on Dataverse at: https://doi.org/10.7910/DVN/OQHLAJ

and contact information or URL).<br>• This text is appropriate if the data are owned by a third party and authors do not have permission to share the data.<br><br>* typeset	
Additional data availability information:	

Powered by Editorial Manager® and ProduXion Manager® from Aries Systems Corporation

Manuscript
Click here to access/download;Manuscript;manuscript_revised.pdf

Investigating Italian disinformation spreading on Twitter in the context of 2019 European elections

Francesco Pierri[1,*], Alessandro Artoni[1], Stefano Ceri[1]

1 Dept. of Electronics, Information and Bioengineering, Politecnico di Milano, 20133 Milano, Italy

*Corresponding author. E-mail: francesco.pierri@polimi.it

Abstract

We investigate the presence (and the influence) of disinformation spreading on online social networks in Italy, in the 5-month period preceding the 2019 European Parliament elections. To this aim we collected a large-scale dataset of tweets associated to thousands of news articles published on Italian disinformation websites. In the observation period, a few outlets accounted for most of the deceptive information circulating on Twitter, which focused on controversial and polarizing topics of debate such as immigration, national safety and (Italian) nationalism. We found evidence of connections between different disinformation outlets across Europe, U.S. and Russia, which often linked to each other and featured similar, even translated, articles in the period before the elections. Overall, the spread of disinformation on Twitter was confined in a limited community, strongly (and explicitly) related to the Italian conservative and far-right political environment, who had a limited impact on online discussions on the up-coming elections.

Introduction

In recent times, growing concern has risen over the presence and the influence of deceptive information spreading on social media [1]. The research community has employed a variety of different terms to indicate the same issue, namely disinformation, misinformation, propaganda, junk news and false (or "fake") news.

As people are more and more suspicious towards traditional media coverage [2], news consumption has considerably shifted towards online social media; these exhibit unique characteristics which favored, among other things, the proliferation of low-credibility content and malicious information [1, 2]. Consequently, it has been questioned in many circumstances whether and to what extent disinformation news circulating on social platforms impacted on the outcomes of political votes [2–5].

Focusing on 2016 US Presidential elections, recent research has shown that false news spread deeper, faster and broader than reliable news [6], with social bots and echo chambers playing an important role in the diffusion of deceptive information [7, 8]. However, it has also been highlighted that disinformation only amounted to a negligible

fraction of online news [9–11], the majority of which were exposed to and shared by a restricted community of old and conservative leaning people, highly engaged with political news [9–11]. In spite of such small volumes, a study suggested that false news (and the alleged interference of Russian trolls) played an important role in the election of Donald Trump [2].

As the European Union (EU) failed to counter the debt crisis which took place since the end of 2009 (following 2008 financial crisis in the US), populist and anti-establishment movements slowly formed up against EU which was now seen as a purely bureaucratic elite [12]. After 2016 Brexit Referendum, several parties embodying these ideals gained a lot of consensus in political elections across different countries (e.g. Hungary, France, Austria, Italy), building their propaganda on a so-called principle of sovereignty, claiming authority over flexibility clauses (which had previously led to austerity policies) and the Schengen treaty, which allows free movement, even willing to leave the EU [13]. As Europeans were called to elect their new representatives at the European Parliament–between the 23rd and the 26th of May 2019–traditional parties, such as European People's Party (EPP), Socialists and Democrats (S&D) and Alliance of Liberals and Democrats for Europe (ALDE), opposed a more cohesive yet renewed vision of Europe. Eventually, the pro-European side prevailed on aforementioned disruptive forces in all countries, with the only exception of Italy where "Lega" amplified its consensus (33%) and instead "Movimento 5 Stelle" declined (18%). Outside of our scope, a change of government occurred during the Summer of 2019.

For what concerns misbehavior on social platforms in European countries, recent research has highlighted the impact and the influence of social bots and online disinformation in different circumstances, including 2016 Brexit [5], 2017 French Presidential Elections [4,14] and 2017 Catalan referendum [15]. A significant presence of junk news in online conversations concerning 2019 European elections has been recently reported across several countries [14,16–18]. The European Commission has itself raised concerns–since 2015 [19]–about the large exposure of citizens to disinformation, promoting an action plan to build capabilities and enforce cooperation between different member states. In anticipation of 2019 European Parliament elections, they sponsored an ad-hoc fact-checking portal (`www.factcheck.eu`) to debunk false claims relative to political topics, aggregating reports from several agencies across different countries.

For what concerns Italy, according to Reuters [20], trust in news is today particularly low (40% of people trust overall news most of the time, 23% trust news in social media most of the time), as result of a long-standing trend which is mainly due to the political polarization of mainstream news organizations and of the resulting partisan nature of Italian journalism. Previous research on online news consumption highlighted the existence of segregated communities [21] and explored the characteristics of polarizing and controversial topics which are traditionally prone to misinformation [22]. Remarkable exposure to online disinformation was highlighted by authors of [23], who exhaustively investigated online media coverage in the run-up to 2018 Italian General elections; in particular, the study observed a rising trend in the spread of malicious information, with a peak of interactions in correspondence with the Italian elections. This result was later substantiated in a report of the Italian Authority for Communications Guarantees (AGCOM) [24]. A very recent work [25] has collected electoral and socio-demographic data, relative to Trentino and South Tyrol regions, as to directly estimate the impact of fake news on the 2018 electoral outcomes, with a focus on the populist vote; this study argues that malicious information had a negligible and non-significant effect on the vote. Furthermore, a recent investigation by Avaaz [26] revealed the existence of a network of Facebook pages and fake accounts which spread low-credibility and inflammatory content–reaching over a million interactions–in explicit support of ”Lega”, ”Movimento 5 Stelle” about controversial themes such as immigration, national safety and anti-establishment. Those pages were eventually shut down by Facebook as violating the platform's terms of use.

In this work we focus on the 5-month period preceding 2019 European elections; we carry out our research on a consolidated setting, described in [8, 27], for investigating the presence (and the impact) of disinformation in the Italian Twittersphere. We recognize that our analysis has a few inherent limitations: first, according to Reuters [20] Twitter is overtaken by far by other social platforms, accounting for only 8% of total users (with a decreasing trend) when it comes to consume news online compared to Instagram (13%), YouTube (25%), WhatsApp (27%) and Facebook (54%), which exhibit instead a rising trend. Second, these differences are even more accentuated when comparing with the U.S. scenario [24], the focus of most of recent research. However, other aforementioned social media offer today little opportunities to

researchers to conveniently analyze the spread of online information, given the limitations they impose on the acquisition of data and the different user experiences they offer. Our study sheds light on the Italian mechanisms of disinformation spreading, and thus the outcomes of the analysis indicate directions for future research in the field.

To collect relevant data, we manually curated a list of websites which have been flagged by fact-checking agencies for fabricating and spreading a variety of malicious information, namely inaccurate and misleading news reports, hyper-partisan and propaganda stories, hoaxes and conspiracy theories. Differently from [8], satire was excluded from the analysis. Following literature on the subject [3, 7, 9–11], we used a "source-based" approach, and assumed that all articles published on aforementioned outlets indeed carried deceptive information; nonetheless, we are aware that this might not be always true and reported cases of misinformation on mainstream outlets are not rare [3]. Our analysis was driven by the following research questions:

RQ1: What was the reach of disinformation which circulated on Twitter in the run-up to European Parliament elections? How active and strong was the community of users sharing disinformation?

RQ2: What were the most debated themes of disinformation? How much were they influenced by national vs European-scale topics?

RQ3: Who are the most influential spreaders of disinformation? Do they exhibit precise political affiliations? How could we dismantle the disinformation network?

RQ4: Did disinformation outlets organize their deceptive strategies in a coordinated manner? Can we identify inter-connections across different countries?

We first describe the data collection and the methodology employed to perform our analysis, then we discuss each of the aforementioned research questions, and finally we summarize our findings.

Fig 1. Time series for the number of tweets, containing links to disinformation articles, collected in the period from 07/01/2019 to 27/05/2019. We annotated it with some events of interest.

Methods

Data Collection

Following a consolidated strategy [7, 8, 27], we leveraged Twitter Streaming API in order to collect tweets containing an explicit Uniform Resource Locator (URL) associated to news articles shared on a set of Italian disinformation websites. As a matter of fact, using the standard streaming endpoint allows to gather 100% of shared tweets matching the defined query (see next) [27].

To this aim we manually compiled a list of 63 disinformation websites that were still active in January 2019. We relied on blacklists curated by local fact-checking organizations (such as "butac.net", "bufale.net" and "pagellapolitica.it"); these include websites and blogs which share hyper-partisan and conspiratorial news, hoaxes, pseudo-science and satire. We initially started with only a dozen of websites, and we successively added other sources; this did not alter the overall collection procedure.

For sake of comparison, we also included four Italian fact-checking and debunking agencies, namely "lavoce.info", "pagellapolitica.it", "butac.net", "bufale.org".

In accordance with current literature [6, 9–11, 27] we use a "source-based" approach: we do not verify each news article manually but we assign the *disinformation* label to all items published on websites labeled as such (the same holds for *fact-checking* articles).

In order to filter relevant tweets, we used all domains as query `filter` parameters (dropping "www", "https", etc) in the form "`byoblu com OR voxnews info OR ...`" as suggested by Twitter Developers guide (`https://developer.twitter.com`). We built a crawler to visit these websites and parse URLs as to extract article text and other metadata (published date, author, hyperlinks, etc). We handled URL duplicates by directly visiting hyperlinks and comparing the associated HTML content. We also extracted profile information and Twitter timelines for all users using Twitter API.

The collection of tweets containing disinformation (see Fig 1) and fact-checking articles was carried out continuously from January 1st (2019) to May 27th, the day after EU elections in Italy. We collected 16,867 disinformation articles shared over

Fig 2. Time series for the number of shares on both Twitter (red) and Facebook (blue) for two disinformation outlets, respectively "byoblu.com" (left) and "silenziefalsita.it" (right), in the period from 07/01/2019 to 27/05/2019.

354,746 tweets by 23,243 unique users, and 1,743 fact-checking posts shared over 23,215 tweets by 9814 unique users.

We can observe that, in general, articles devoted to debunk false claims were barely engaged, accounting only for 6% of the total volume of tweets spreading disinformation in the same period; such findings are comparable with the US scenario [8], and they are in accordance with the very low effectiveness of debunking strategies which is documented in [28]. We leave for future research an in-depth comparative analysis of diffusion networks pertaining to the two news domains.

The entire data is available at: `https://doi.org/10.7910/DVN/OQHLAJ`

Comparison with Facebook

In order to perform a rough estimate of the different reach of disinformation on Twitter compared to Facebook, we collected data relative to the latter platform regarding two disinformation outlets, namely "byoblu.com" and "silenziefalsita.it", which have an associated Facebook page and are among Top-3 prolific and engaged sources of malicious information (see Results).

We used `netvizz` [29] to collect statistics on the number of daily shares of Facebook posts published by aforementioned outlets, and we compared with the traffic observed on Twitter. As we can see in Fig 2, disinformation has a stronger reach on Facebook than Twitter, for both sources, throughout the observation period; this is also shown in other works [23, 24, 26], coherently with the Italian consumption of social news. An in-depth analysis of the Italian disinformation on Facebook would be required, but it needs suitable assistance from Facebook for what concerns the disinformation diffusion network.

Network analysis

Building Twitter diffusion network

We built a global diffusion network–corresponding to the union of all sharing cascades

associated to articles gathered in our dataset–following a consolidated strategy [7, 8]. We considered different Twitter social interactions altogether and for each tweet we add nodes and edges differently according to the action(s) performed by users:

- **Tweet**: a basic tweet corresponds to originally authored content, and it thus identifies a single node (author).
- **Mention**: whenever a tweet of user a contains a mention to user b, we build an edge from the author a of the tweet to the mentioned account b.
- **Reply**: when user a replies to user b we build an edge from a to b.
- **Retweet**: when user a retweets another account b, we build an edge from b to a.
- **Quote**: when user a quotes user b the edges goes from b to a.

When processing tweets, we add a new node for users involved in aforementioned interactions whenever they are not present in the network. As a remark, a single tweet can contain simultaneously several actions and thus it can generate multiple nodes and edges. Finally, we consider edges to be weighted, where the weight corresponds to the number of times two users interacted via actions mentioned beforehand.

Building the network of websites

In order to investigate existing inter-connections among different disinformation websites, and to understand the nature of external sources which are usually mentioned by deceptive outlets, we searched for URLs in all articles present in our dataset, i.e. which were shared at least once on Twitter. We accordingly built a graph where each node is a distinct Top-Level Domain–the highest level in the hierarchical Domain Name System (DNS) of the Internet–and an edge is built between two nodes a and b whenever a has published at least an article containing an URL belonging to b domain; the weight of an edge corresponds to the number of shared tweets carrying an URL with an hyperlink from a to b. The final result is a directed weighted network of approximately 5k nodes and 8k edges. We used `networkx` Python package [30] to handle the network.

Main core decomposition, centrality measures and community detection

In our analysis we employed several techniques coming from the network science toolbox [31], namely k-core decomposition, community detection algorithms and centrality measures. We used `networkx` Python package to perform all the computations.

The k-core [32] of a graph G is the maximal connected sub-graph of G in which all vertices have degree at least k. Given the k-core, recursively removing all nodes with degree k allows to extract the $(k+1)$-core; the main core is the non-empty graph with maximum value of k. k-core decomposition can be employed as to uncover influential nodes in a social network [8].

Community detection is the task of identifying *communities* in a network, i.e. dense sub-graphs which are well separated from each other [33]. In this work we consider Louvain's fast greedy algorithm [34], which is an iterative procedure that maximizes the Newman-Girvan *modularity* [35]; this measure is based on randomizations of the original graph as to check how non-random the group structure is.

A centrality measure is an indicator that allows to quantify the importance of a node in a network. In a weighted directed network we can define the *In-strength* of a node as the sum of the weights on the incoming edges, and the *Out-strength* as the sum of the weights on the out-going edges. *Betweenness* centrality [36] instead quantifies the probability for a node to act as a bridge along the shortest path between two other nodes; it is computed as the sum of the fraction of all-pairs shortest paths that pass through the node. *PageRank* centrality [37] is traditionally used to rank webpages in search engine queries; it counts both the number and quality of links to a page to estimate the importance of a website, assuming that more important websites will likely receive more links from other websites.

Time series analysis

In our experiments, we carried out a trend analysis of time series concerning users' activity, topics contained in disinformation articles and the number of interconnections between different outlets.

In statistics, a trend analysis refers to the task of identifying a population

characteristic changing with another variable, usually time or spatial location. Trends can be increasing, decreasing, or periodic (cyclic). We used the Mann-Kendall statistical test [38, 39] as to determine whether a given time series showed a monotonic trend. The test is non-parametric and distribution-free, e.g. it does not make any assumption on the distribution of the data. The null hypothesis H_0, no monotonic trend, is tested against the alternative hypothesis H_a that there is either an upward or downward monotonic trend, i.e. the variable consistently increases or decreases through time; the trend may or may not be linear. We used `mkt` Python package.

The multiple testing (or large-scale testing) problem occurs when observing simultaneously a set of test statistics, to decide which if any of the null hypotheses to reject [40]. In this case it is desirable to have confidence level for the whole family of simultaneous tests, e.g. requiring a stricter significance value for each individual test. For a collection of null hypotheses we define the family-wise error rate (FWER) as the probability of making at least one false rejection, (at least one type I error). We used the classical *Bonferroni* correction to control the FWER at $\leq \alpha$ by strengthening the threshold of each individual testing, i.e. for an overall significance level α and N simultaneous tests, we reject the individual null hypothesis at significance level α/N.

Ethics statement

We do not need ethical approval as data was publicly available and collected through Twitter Streaming API; we do not infringe Twitter terms and conditions of use. The same holds for data relative to Facebook, which was obtained using `netvizz` application in accordance with their terms of service.

Results and discussion

Assessing the reach of Italian disinformation

Sources of disinformation

To understand the reach of different disinformation outlets, we first computed the distribution of the number of articles and tweets per source. We observed, as shown in Fig 3, that a few websites dominate on the remaining ones both in terms of activity and

Fig 3. A (Top). The distribution of the total number of shared articles per website. **B (Bottom).** The distribution of the total number of associated tweets per website. We show Top-11 (which account for over 95% of the total volume of tweets), and we aggregate remaining sources as "Others".

social audience.

In particular, with approximately 200k tweets (over 50% of the total volume) and 6k articles (about 1/3 of the total number), "voxnews.info" stands out on all other sources; this outlet spreads disinformation spanning several subjects, from immigration to health-care and conspiratorial theories, and it runs campaigns against fact-checkers as well as labeling its articles with false "fact-checking" labels as to deceive readers.

Interestingly, two other uppermost prolific sources such as "skynew.it" and "tuttiicriminidegliimmigrati.com" do not receive the same reception on the platform; the former has stopped its activity on March and the latter is literally–it translates as "All the immigrants crimes"–a repository of true, false and mixed statements about immigrants who committed crimes in Italy.

We can also recognize three websites associated to public Facebook pages that have been recently banned after the investigation of Avaaz NGO, namely "jedanews.it", "catenaumana.it" and "mag24.es", as they were "regularly spreading fake news and hate speech in Italy" violating the platform's terms of use [26].

We further computed the distribution of the daily engagement (the ratio `no.articles published/no.tweets shared` per day) per each source, noticing that a few sources exhibit a considerable number of social interactions in spite of fewer associated tweets, compared to uppermost "voxnews.info". We show the time series for the daily engagement of Top-10 sources, which account for over 95% of total tweets, in Fig 4. We can notice in particular that "byoblu.com" exhibits remarkable spikes of engagement w.r.t to a very small number of total tweets compared to other outlets, whereas "mag24.es" shows a suspiciously large number of shares in the month preceding the elections (and after the release of Avaaz report).

We excluded "ilprimatonazionale.it" from this analysis as it was added only at the end of April (we collected around 30k associated tweets and less than 1000 articles); official magazine of "CasaPound" (former) neo-fascist party–with style and agenda-setting that remind of Breitbart News–it exhibits a daily engagement of over 200

Fig 4. Daily engagement for Top-10 sources (ranked according to the total number of shared tweets). The Mann-Kendall test (upward trend at significance level 0.005) was accepted only for ”byoblu.com”.

tweets, exceeding all other websites .

As elections approached, we were interested to understand whether there were particular trends in the daily reception of different sources. Focusing on Top-10 sources (except ”ilprimatonazionale.it”) we performed a Mann-Kendall test to assess the presence of an upward or downward monotonic trend in the time series of (a) daily shared tweets and (b) daily engagement. Taking into account Bonferroni's correction, the test was rejected at $\alpha = 0.05/10 = 0.005$; both (a) and (b) exhibit an upward trend for ”byoblu.com” alone, whereas the remaining sources are either stationary or monotonically decreasing. As this outlet strongly supported euro-skeptical positions (and often gave visibility to many Italian representatives of such arguments) we argue that in the run-up to the European elections its agenda became slightly more captivating for the social audience.

User activity

For what concerns the underlying community of users sharing disinformation, we first computed the distribution of the number of shared tweets and unique URLs shared per number of users, noticing that a restricted community of users is responsible for spreading most of the online disinformation. In fact, approximately 20% of the community (∼4k users) accounts for more than 90% of total tweets (∼330k), in accordance with similar findings elsewhere [8–10]. Among them, we identified accounts officially associated to 18 different outlets (we manually looked at users' profile description and usernames); they overall shared 8310 tweets.

We also distinguished five classes of users based on their generic activity, i.e. the number of shared tweets containing an URL to disinformation articles: *Rare* (about 9.5k users) with only 1 tweet; *Low* (about 8k users) with more than 1 tweet and less than 10; *Medium* (about 3k users) with a number of tweets between 11 and 100; *High* (about 500 users) with more than 100 tweets but less than 1000; *Extreme* (exactly 20 users) with more than 1000 shared tweets. About 1 user out of 5 shared more than 10 disinformation articles in five months.

Fig 5. A (Top). A breakdown of the total volume of tweets according to the activity of users. Fractions of users created in the six months before the elections are indicated with lighter shades; these account respectively for 0.18% (*Rare*), 0.6% (*Low*), 2.04% (*Medium*) and 2.98% (*High*) of total tweets.
B (Center). The distribution of the number of users per retweeting activity.
C (Bottom). The distribution of daily tweets shared by recently created users.

As shown in Fig 5A, we can notice that a minority of very active users (the ensemble with *High* and *Extreme* activity) accounts for half of the deceptive stories that were shared, and over 3/4 of the total number of tweets was shared by less than 4 thousand users (*Medium*, *High* and *Extreme* activity).

We overall report 21,124 active (20 of which are also verified), 800 deleted, 124 protected and 112 suspended accounts. Verified accounts were altogether involved in 5761 tweets, only 18 of which in an "active" way, i.e. a verified account actually authored the tweet. We observed that they were mostly called in with the intent to mislead their followers, adding deceptive content on top of quoted statuses or replies.

Next we inspected the distribution of the number of users concerning their re-tweeting activity, i.e. the fraction of re-tweets compared to the number of pure tweets; as shown in Fig 5B this is strongly bi-modal, and it reveals that users sharing disinformation are mostly "re-tweeters": more than 60% of the accounts exhibit a re-tweeting activity larger than 0.95 and less than 30% have a re-tweeting activity smaller than 0.05. This shows that a restricted group of accounts is presumably responsible for conveying in the first place disinformation articles on the platform, which are propagated afterwards by the rest of the community.

We computed the distribution of some user profile features, namely the count of followers and friends, the number of statuses authored by users and the age on the social platform (in number of months passed since the creation date to May 2019). We report that users sharing disinformation tend to be quite "old" and active on the platform–with an average age of 3 years and more than a thousand authored statuses. We were able to gather information via Twitter API only for active and non-protected users.

We further inspected recently created accounts, noticing that approximately a thousand user was registered during the collection period, i.e. the last six months; they show similar distributions of aforementioned features compared to older users. Overall (see Fig 5B) they mostly pertain to active classes (*Medium* and *High*) and they account

for 15% (around 18k tweets) of the total volume of tweets considered–which lowers to approximately 288k tweets excluding those authored by non-active, suspended and protected accounts. Furthermore, about a hundred exhibit abnormal activities, producing more than 10k (generic) tweets in the period preceding the elections and directly sharing more than 10 disinformation stories each. We performed a Mann-Kendall test to the time series of daily tweets shared by such users (see Fig 5C), assessing the presence of a monotonically increasing trend (at significance level $\alpha = 0.05$). The main referenced source of disinformation is "voxnews.info" with more than 60% (circa 12k tweets) of the total number of shared stories. An activity of this kind is quite suspicious and could be further investigated as to detect the presence of "cyber-troops" (bots, cyborgs or trolls) that either attempted to drive public opinion in light of up-coming elections (via so-called "astroturfing" campaigns [41]) or simply redirected traffic as to generate online revenue through advertisement [1–3].

The agenda-setting of disinformation

Topic analysis

For what concerns the main themes covered by different disinformation outlets, relative to the resulting audience on Twitter, we based our analysis on the first level of agenda-setting theory [42], which states that news media set the public importance for objects based on the frequency in which these are mentioned and covered. In the case of fake news an agenda-setting effect could occur as a result of the rise in the coverage, even if some audience members are aware that fake news is fake [43]. We focused on the prevalence of titles, which were shared at least once, as they usually pack a lot of information about their claims in simple and repetitive structures [44]; besides, the exposure such as the presence alone of misleading titles on users' timelines could affect ordinary beliefs and result in a resistance to opposite arguments [28] and an increased perceived accuracy of the content, irrespective of its credibility [45].

We avoided automatic topic modeling algorithms [46] as they are not suitable for small texts. Therefore we carried out a topic analysis with a dictionary-based approach, and we manually compiled a list of keywords associated to five distinct topics namely: Politics/Government (PG), Immigration/Refugees (IR), Crime/Society (CS),

Fig 6. A stacked-area chart showing the distribution of different topics over the collection period. The daily coverage on themes related to Immigration/Refugees and Europe/Foreign is stationary, whereas focus on subjects related to Crime/Society and Politics/Government is monotonically increasing towards the elections (end of May 2019).

**Politics/Government**	**Immigration/Refugees**	**Europe/Foreign**	**Crime/Society**	**Other**
salvini	immigrati	euro	rom	video
italia	profughi	europa	milano	anni
pd	clandestini	ue	casa	contro
italiani	profugo	fusaro	bergoglio	foto
m5s	ong	diego	morti	vuole
italiana	porti	meluzzi	mafia	può
italiano	migranti	libia	bambini	vogliono
milioni	africani	macron	roma	parla
lega	immigrato	soros	donne	byoblu
sinistra	islamici	francia	bruciato	via
casapound	imam	francesi	confessa	niccolò
maio	seawatch	gilet	falsi	casal
soldi	nigeriani	gialli	bus	vero
guerra	nigeriana	europee	choc	ufficiale
cittadinanza	nigeriano	germania	figli	bufala
prima	islamica	tedesca	case	anti
raggi	africano	mondo	chiesa	sta
governo	stranieri	notre	famiglia	grazie
renzi	chiusi	dame	magistrato	casarini
zingaretti	sea	francese	polizia	farli

Table 1. Top-20 keywords associated with each topic.

Europe/Foreign (EF), Other (OT). Keywords were obtained with a data-driven approach, i.e. inspecting Top-500 most frequent words appearing in the titles, and taking into account relevant events that occurred in the last months. We provide Top-20 keywords for each topic in Table 1.

In particular, PG refers to main political parties and state government as well as the main political themes of debate. IR includes references to immigration, refugees and hospitality whereas CS includes terms mostly referring to crime, minorities and national security. Finally EF contains direct references to European elections and foreign countries. It is worth mentioning that the most frequent keyword was ”video”, suggesting that a remarkable fraction of disinformation was shared as multimedia content [47].

We computed the relative presence of each topic in each article and accordingly assessed their distribution across tweets over different months. We can observe in Fig 6 that the discussion was stable on controversial topics such immigration, refugees, crime and government, whereas focus on European elections and foreign affairs was quite negligible throughout the period, with only a single spike of interest at the beginning of

Fig 7. Top-10 hashtags per number of shared tweets (blue) and unique users (orange).

January corresponding to the quarrel between Italian and France prime ministers. We also performed Mann-Kendall test to assess the presence of any monotonic trends in the daily distribution of different topics; we rejected the test for $\alpha = 0.05/5 = 0.01$ for IR and EF whereas we accepted it for the remaining topics, detecting the presence of an upward monotonic trend in CS and PG, and a downward monotonic trend in OT.

In the observation period, the disinformation agenda was well settled on main arguments supported by leading parties, namely ”Lega” and ”Movimento 5 Stelle”, since 2018 general elections; this suggests that they might have profited from and directly exploited hoaxes and misleading reports as to support their populist and nationalist views (whereas ”Partito Democratico” appeared among main targets of misinformation campaigns); empirical evidence for this phenomenon has been also widely reported elsewhere [23, 25]. However, the electoral outcome confirmed the decreasing trend of ”Movimento 5 Stelle” electoral consensus in favor of ”Lega”, which was rewarded with an unprecedented success.

Differently from 2018 [23] we in fact observed one main cited leader: Matteo Salvini (”Lega” party). This is consistent with a recent report on online hate speech [48], contributed by Amnesty International, which has shown that his activity (and reception) on Twitter and Facebook is 5 times higher than Luigi Di Maio (leader of ”Movimento 5 Stelle”); not surprisingly, his main agenda focuses (negatively) on immigration, refugees and Islam (which generated most of online interactions in 2018 [23]), which are also the main objects of hate speech and controversy in online conversations of Italian political representatives overall.

It appears that mainstream news actually disregarded European elections in the months preceding them, focusing on arguments of national debate [49]; this trend was also observed in other European countries according to FactCheckEU [50], claiming that misinformation was not prominent in online conversations mainly because European elections are not particularly polarized and are seen as less important compared to national elections. We believe that this might have affected the agenda of disinformation outlets, which are in general susceptible to traditional media coverage [51], thus explaining the focus on different targets in their deceptive strategies.

Fig 8. The cloud of words for Top-50 most frequent hashtags embedded in the users' profile description.

Usage of hashtags

Among most relevant hashtags shared along with tweets–in terms of number of tweets and unique users who used them (see Fig 7)–a few indicate main political parties (cf. "m5s", "pd", "lega") and others convey supporting messages for precise factions, mostly "Lega" (cf. "salvininonmollare", "26maggiovotolega"); some hashtags manifest instead active engagement in public debates which ignited on polarizing and controversial topics (such as immigrants hospitality, vaccines, the Romani community and George Soros). We also found explicit references to (former) far-right party "CasaPound" and the associated "Altaforte" publishing house, as well as some disinformation websites (with a remarkable polarization on "criminiimmigrati" which was shared more than 5000 times by only a few hundred accounts).

We also extracted hashtags directly embedded in the profile description of users collected in our data, for which we provide a cloud of words in Fig 8. The majority of them expresses extreme positions in matter of Europe and immigration: beside explicit references to "Lega" and "Movimento 5 Stelle", we primarily notice euro-skeptical (cf. "italexit", "noue"), anti-Islam (cf. "noislam") and anti-immigration positions (cf. "noiussoli", "chiudiamo i porti") and, surprisingly enough, also a few (alleged) Trump followers (cf. "maga" and "kag"). The latter finding is odd but somehow reflects the vicinity of Matteo Salvini and Donald Trump on several political matters (such as refugees and national security). On the other hand, we also notice "facciamorete", which refers to a Twitter grassroots anti-fascist and anti-racist movement that was born on December 2018, as a reaction to the recent policies in matter of immigration and national security of the Italian establishment.

Principal spreaders of disinformation

Central users in the main core

In order to identify most influential nodes in the diffusion network, we computed the value of several centrality measures for each account. We show in Table 2 the list of Top-10 users according to each centrality measure, and we also indicate whether they

Table 2. List of Top-10 users according to different centrality measures, namely In-strength, Out-Strength, Betweenness and PageRank; we indicate with a cross nodes that do not belong to the main K-core (k=47) of the network.

Rank	In-Strength	Out-Strength	Betweenness	PageRank
**1**	napolinordsud ×	Filomen30847137	IlPrimatoN	IlPrimatoN
**2**	RobertoPer1964	POPOLOdiTW1TTER	matteosalvinimi	matteosalvinimi
**3**	razorblack66	laperlaneranera	Filomen30847137	Sostenitori1 ×
**4**	polizianuovanaz ×	byoblu	byoblu	armidmar
**5**	Giulia46489464	IlPrimatoN	a_meluzzi	Conox_it ×
**6**	geokawa	petra_romano	AdryWebber	lauraboldrini ×
**7**	Gianmar26145917	araldoiustitia	claudioerpiu	pdnetwork ×
**8**	pasqualedimaria ×	max_ronchi	razorblack66	libreidee ×
**9**	il_brigante07	Fabio38437290	armidmar	byoblu
**10**	AngelaAnpoche	claudioerpiu	Sostenitori1 ×	Pontifex_it ×

belong or not to the main K-core of the network [32]; this corresponds to the sub-graph of neighboring nodes with degree greater or equal than $k = 47$, which is shown in Fig 9. We color nodes according to the communities identified by the Louvain modularity-based community algorithm [34] run on the original diffusion network (over 20k nodes and 100k edges).

Although we expect centrality measures to display small differences in their ranking, we can notice that the majority of nodes with highest values of In-Strength, Out-Strength and Betweenness centralities also belong to the main K-core of the network; the same does not hold for users which have a large PageRank centrality value. A few users strike the eye:

1. `matteosalvinimi` is Matteo Salvini, leader of the far-right wing "Lega" party; he is not an active spreader of disinformation, being responsible for just one (true) story coming from disinformation outlet "lettoquotidiano.com" (available at `https://twitter.com/matteosalvinimi/status/1102654128944308225`), which was shared over 1800 times. He is generally passively involved in deceptive strategies of malicious users who attempt to "lure" his followers by attaching disinformation links in replies/re-tweets/mentions to his account.

2. `a_meluzzi` is Alessandro Meluzzi, a former representative of centre-right wing "Forza Italia" party (whose leader is Silvio Berlusconi); he is a well-known supporter of conspiracy theories and a very active user in the disinformation network, with approximately 400 deceptive stories shared overall.

3. Accounts associated to disinformation outlets, namely `IlPrimatoN` with

Fig 9. The main K-core ($k = 47$) of the re-tweeting diffusion network. Colors correspond to different communities identified with the Louvain's algorithm. Node size depends on the total Strength (In + Out) and edge color is determined by the source node.

"ilprimatonazionale.it", `byoblu` with "byoblu.com", `libreidee` with "libreidee.org", `Sostenitori1` with "sostenitori.info" and `Conox_it` with "conoscenzealconfine.it".

A manual inspection revealed that most of the influential users are indeed actively involved in the spread of disinformation, with the only exception of `matteosalvinimi` who is rather manipulated by other users, via mentions/retweets/replies, as to mislead his huge community of followers (more than 2 millions). The story shared by Matteo Salvini underlines a common strategy of disinformation outlets identified in this analysis: they often publish simple true and factual news as to bait users and expose them to other harmful and misleading content present on the same website.

Besides, we recognized a few influential users who are (or have been in the past) target of several disinformation campaigns:

1. `lauraboldrini` is Laura Boldrini, representative of left-wing "Liberi e Uguali" party and actual member of the Italian Parliament; in the last few years she has been repetitively a target of fake news.

2. `pdnetwork` is the account of the centre-left "Partito Democratico" party; as the former ruling party it has been severely attacked in the propaganda of both actual "Lega" and "Movimento 5 Stelle".

3. `Pontifex_it` is the account of Papa Francesco; due to his recent statements showing empathy for migrants he has become another target of Italian far-right online hateful speech.

We also report a suspended account (`polizianuovanaz`), a protected one (`Giulia46489464`) and a deleted user (`pasqualedimaria`).

In addition, we investigated communities of users in the main K-core–which contains 218 nodes (see Fig 9)–and we noticed systematic interactions between distinct accounts. We manually inspected usernames, most frequent hashtags and referenced sources, deriving the following qualitative characterizations:

1. the **Green** community corresponds to "Lega" party official accounts: `matteosalvinimi` and `legasalvini`, whereas the third account, `noipersalvini`, belongs to the same community but does not appear in the core.

2. the **Red** community represents Italian far-right supporters, with several representatives of CasaPound (former) party (including his secretary `distefanoTW` who does not appear in the core), who obviously refer to "ilprimatonazionale.it" news outlet.

3. the **Yellow** community is strongly associated to two disinformation outlets, namely "silenziefalsita.it" (`SilenzieFalsita`) and "jedanews.it" (`jedasupport`); the latter was one of the pages identified in Avaaz report [26] and deleted by Facebook.

4. the **Orange** community is associated to the euro-skeptical and conspiratory outlet "byoblu.com" (`byoblu`), and it also features Antonio Maria Rinaldi (`a_rinaldi`), a well-known euro-skeptic economist who has just been elected with "Lega" in the European Parliament.

5. the **Purple** community corresponds to the community associated to "tuttiicriminidegliimmigrati.com" (`TuttICrimin`) disinformation outlet.

6. the remaining **Blue** (`Filomen30847137`), **Light-blue** (`araldoiustitia`) and **Brown** communities (`petra_romano`) represent different groups of very active "loose cannons" who do not exhibit a clear affiliation.

Eventually, we employed Botometer algorithm [52] as to detect the presence of social bots among users in the main core of the network. We set a threshold of 50% on the Complete Automation Probability (CAP)–i.e. the probability of an account to be completely automated–which, according to the authors, is a more conservative measure that takes into account an estimate of the overall presence of bots on the network; besides, we computed the CAP value based on the language independent features only, as the model includes also some features conceived for English-language users. We only detected two bot-like accounts, namely `simonemassetti` and `jedanews`, respectively with probabilities 58% and 64%, that belong to the same Purple community. A manual check confirmed that the former habitually shares random news content (also

Fig 10. Results of different network dismantling strategies w.r.t to remaining unique disinformation articles in the network. The x-axis indicates the number of disconnected accounts and the y-axis the fraction of remaining items in the network.

mainstream news) in an automatic flavour whereas the latter is the official spammer account of ”jedanews.it” disinformation outlet. We argue that the impact of automated accounts in the diffusion of malicious information is quite negligible compared to findings reported in [8], where about 25% of accounts in the main core of the US disinformation diffusion network were classified as bots.

Dismantling the disinformation network

Similar to [8], we performed an exercise of network dismantling analysis using different centrality measures, as to investigate possible intervention strategies that could prevent disinformation from spreading with the greatest effectiveness.

We first ranked nodes in decreasing order w.r.t to each metric, plus the core number–the largest k for which the node is present in the corresponding k-core–and the In and Out-degree, which exhibited the same Top 10 ranking as their weighted formulation (Strengths), but they do entail different results at dismantling the network. Next we delete them one by one while tracking the resulting fraction of remaining edges, tweets and unique articles in the network.

We observed that eliminating a few hundred nodes with largest values of Out-Degree promptly disconnects the network; in fact these users alone account for 90% of the total number of interactions between users. For what concerns the number of tweets sharing disinformation articles, the best strategy would be to target users with largest values of In-Strength who, according to our network representation, are likely to be users with a high re-tweeting activity; in fact, confirming previous observations, a few thousand nodes account for more than 75% of the total number of tweets shared in the five months before the elections. However, as shown in Fig 10, it is more challenging to prevent users to be exposed from even a tiny fraction of disinformation articles, as the network exhibits an almost linear relationship between the number of users disconnected and the corresponding number of remaining stories; as such the spread of malicious information would be completely prevented only blocking the entire network.

Fig 11. Two different views of the network of websites; the size of each node is adjusted w.r.t to the Out-strength, the color of edges is determined by the target node and the thickness depends on the weight (i.e. the number of shared tweets containing an article with that hyperlink).
A (Left). The main core of the network ($k = 14$); blue nodes are Italian disinformation websites, green ones are Italian traditional news outlets, red nodes are social networks, the sky-blue node is a video sharing website and the pink one is an online encyclopedia.
B (Right). The sub-graph of Russian (orange), EU (olive green), US (violet) and Italian (blue) disinformation outlets.

Coordinated strategies of deception

To investigate existing connections between different disinformation outlets and other external sources, we first analyzed the network of websites with a core decomposition [32], obtaining a main core ($k = 14$) which contains 35 nodes as a result of over 75,000 external re-directions via hyperlinks (shown in Fig 11A). Over 99% of the articles includes a hyperlink in the body. We may first notice frequent connections between distinct disinformation outlets, suggesting the presence of shared agendas and presumably coordinated deceptive tactics, as well as frequent mentions to reputable news websites; among them we distinguish ”IlFattoQuotidiano”, which is a historical supporter of ”Movimento 5 Stelle”, and conservative outlets such as ”IlGiornale” and ”LiberoQuotidiano” which lean instead towards ”Lega”. We also observe that most of the external re-directions point to social networks (Facebook and Twitter) and video sharing websites (Youtube); this is no wonder given that disinformation is often shared on social networks as multimedia content [1,3]. In addition, we inspected nodes with the largest number of incoming edges (In-degree) in the original network, discovering among uppermost 20 nodes a few misleading reports originated on dubious websites (such as ”neoingegneria.com”), flagged by fact-checkers but that were not included in any blacklist. We believe that a more detailed network analysis could reveal additional relevant connections and we leave it for future research.

Furthermore, we focused on the sub-graph composed of three particular classes of nodes, namely Russian (RU) sources, EU/US disinformation websites and our list of Italian (IT) outlets; we manually identified notable Russian sources (”RussiaToday” and ”SputnikNews” networks) and we resorted to notable blacklists to spotlight other EU/US disinformation websites–namely ”opensources.co”, ”décodex.fr”, the list compiled by Hoaxy [27] and references to junk news in latest data memos by

COMPROP research group [14, 16–18].

The resulting bipartite network–we filtered out intra-edges between IT sources to better visualize connections with the "outside" world–contains over 60 foreign websites (RU, US and EU) and it is shown in Fig 11B.

We observe a considerable number of external connections (over 500 distinct hyperlinks present in articles shared more than 5 thousand times) with other countries sources, which were primarily included within "voxnews.info", "ilprimatonazionale.it" and "jedanews.it". Among foreign sources we encounter several well-known US sources ("breitbart.com", "naturalnews.com" and "infowars.com" to mention a few) as well as RU ("rt.com", "sputniknews.com" and associated networks in several countries), but we also find interesting connections with notable disinformation outlets from France ("fdesouche.com" and "breizh-info.com"), Germany ("tagesstimme.com), Spain ("latribunadeespana.com") and even Sweden ("nyheteridag.se" and "samnytt.se"). Besides, a manual inspection of a few articles revealed that stories often originated in one country were immediately translated and promoted from outlets in different countries (see Fig 12). Such findings suggest the existence of coordinated deceptive strategies which span across several countries, consistently with claims in latest report by Avaaz [26] which revealed the existence of a network of far-right and anti-EU websites, leading to the shutdown of hundreds of Facebook pages with more than 500 million views just ahead of the elections. Far-right disinformation tactics comprised the massive usage of fake and duplicate accounts, recycling followers and bait and switch of pages covering topics of popular interest (e.g. sport, fitness, beauty).

It is interesting that Facebook decided on the basis of external insights to shutdown pages delivering misleading content and hate speech; differently from the recent past [3, 7, 8] it might signal that social media are more willing to take action against the spread of deceptive information in coordination with findings from third-party researchers. Nevertheless, we argue that closing malicious pages is not sufficient and more proactive strategies should be followed [3, 26].

Finally, we performed a Mann-Kendall test to see whether there was an increasing trend, towards the elections, in the number of external connections with US and RU disinformation websites; we rejected it at $\alpha = 0.05/2 = 0.0025$.

Fig 12. An example of disinformation story who was published on a Swedish website (”friatider.se”) and then reported by an Italian outlet (”voxnews.info”). Interestingly, this news is old (July 2018) but it was diffused again in the first months of 2019.

Conclusions

We studied the reach of Italian disinformation on Twitter for a period of five months immediately preceding the European elections **(RQ1)** by analyzing the content production of websites producing disinformation, and the characteristics of users sharing malicious items on the social platform. Overall, thousands of articles–which included hoaxes, propaganda, hyper-partisan and conspiratorial news–were shared in the period preceding the elections. We observed that a few outlets accounted for most of the deceptive information circulating on Twitter; among them, we also encountered a few websites which were recently banned from Facebook after violating the platform's terms of use. We identified a heterogeneous yet limited community of thousands of users who were responsible for sharing disinformation. The majority of the accounts (more than 75%) occasionally engaged with malicious content, sharing less than 10 stories each, whereas only a few hundred accounts were responsible for (the spreading) of thousands of articles (see Fig 5).

We singled out the most debated topics of disinformation **(RQ2)** by inspecting news items and Twitter hashtags. We observed that they mostly concern polarizing and controversial arguments of the local political debate such as immigration, crime and national safety, whereas discussion around the topics of Europe global management had a negligible presence throughout the collection period; the lack of European topics was also reported in the agenda of mainstream media.

Then we identified the most influential accounts in the diffusion network resulting from users sharing disinformation articles on Twitter **(RQ3)**, so as to detect the presence of active groups with precise political affiliations. We discovered strong ties with the Italian far-right and conservative community, in particular with ”Lega” party, as most of the users manifested explicit support to the party agenda through the use of keywords and hashtags. Besides, a common deceptive strategy was to passively involve his leader Matteo Salvini via mentions, quotes and replies as to potentially mislead his audience of million of followers. We found limited evidence of bot activity in the main

core, and we observed that disabling a limited number of central users in the network would promptly interrupt to a certain extent the spread of disinformation circulating on Twitter, but it would immediately raise censorship concerns.

Finally, we investigated inter-connections within different deceptive agents **(RQ4)**, thereby observing that they repeatedly linked to each other websites during the period preceding the elections. Moreover we discovered many cases where the same (or similar) stories were shared in different languages across different European countries, as well as U.S. and Russia.

This analysis confirms that disinformation is present on Twitter and that its spread shows some peculiarities in terms of topics being discussed and of political affiliation of the key members of the information spreading community. We are aware that disinformation news in Italy have a higher share on Facebook than Twitter and that the use of Twitter in Italy as a social channel is limited compared to other social platforms such as Facebook, WhatsApp or Instagram. Therefore similar studies on other social media platforms will be needed and beneficial to our understanding of the spread of disinformation.

Acknowledgements

F.P. and S.C. are supported by the PRIN grant HOPE (FP6, Italian Ministry of Education). S.C. is partially supported by ERC Advanced Grant 693174. The authors are very grateful to Hoaxy support team at Bloomington Indiana University.

References

1. Pierri F, Ceri S. False news on social media: a data-driven survey. ACM SIGMOD Record Vol 48 Issue 2 (June). 2019;.

2. Allcott H, Gentzkow M. Social media and fake news in the 2016 election. Journal of Economic Perspectives. 2017;31(2):211–36.

3. Lazer DMJ, Baum MA, Benkler Y, Berinsky AJ, Greenhill KM, Menczer F, et al. The science of fake news. Science. 2018;359(6380):1094–1096. doi:10.1126/science.aao2998.

4. Ferrara E. Disinformation and social bot operations in the run up to the 2017 French presidential election. First Monday. 2017;22(8).

5. Bastos MT, Mercea D. The Brexit botnet and user-generated hyperpartisan news. Social Science Computer Review. 2019;37(1):38–54.

6. Vosoughi S, Roy D, Aral S. The spread of true and false news online. Science. 2018;359(6380):1146–1151. doi:10.1126/science.aap9559.

7. Shao C, Ciampaglia GL, Varol O, Yang KC, Flammini A, Menczer F. The spread of low-credibility content by social bots. Nature communications. 2018;9(1):4787.

8. Shao C, Hui PM, Wang L, Jiang X, Flammini A, Menczer F, et al. Anatomy of an online misinformation network. PLOS ONE. 2018;13(4):1–23. doi:10.1371/journal.pone.0196087.

9. Grinberg N, Joseph K, Friedland L, Swire-Thompson B, Lazer D. Fake news on Twitter during the 2016 U.S. presidential election. Science. 2019;363(6425):374–378. doi:10.1126/science.aau2706.

10. Bovet A, Makse HA. Influence of fake news in Twitter during the 2016 US presidential election. Nature Communications. 2019;10(1):7. doi:10.1038/s41467-018-07761-2.

11. Pierri F, Piccardi C, Ceri S. Topology comparison of Twitter diffusion networks reliably reveals disinformation news. arXiv. 2019;.

12. Henley J. How populism emerged as an electoral force in Europe. The Guardian. 2018;.

13. Dennison S, Zerka P. THE 2019 EUROPEAN ELECTION: HOW ANTI-EUROPEANS PLAN TO WRECK EUROPE AND WHAT CAN BE DONE TO STOP IT. European council on foreign relations. 2019;.

14. Howard PN, Bradshaw S, Kollanyi B, Bolsolver G. Junk News and Bots during the French Presidential Election: What Are French Voters Sharing Over Twitter In Round Two?;.

15. Stella M, Ferrara E, De Domenico M. Bots increase exposure to negative and inflammatory content in online social systems. Proceedings of the National Academy of Sciences. 2018;115(49):12435–12440.

16. Hedman F, Sivnert F, Howard P. News and political information consumption in Sweden: Mapping the 2018 Swedish general election on Twitter; 2018.

17. Kollanyi B, Howard PN. Junk news and bots during the German parliamentary election: What are German voters sharing over Twitter; 2017.

18. Marchal N, Kollanyi B, Neudert LM, Howard PN. Junk News During the EU Parliamentary Elections: Lessons from a Seven-Language Study of Twitter and Facebook. 2019;.

19. Commission E. Tackling online disinformation;. Available from: `https://ec.europa.eu/digital-single-market/en/tackling-online-disinformation`.

20. Nielsen RK, Newman N, Fletcher R, Kalogeropoulos A. Reuters Institute Digital News Report 2019. Report of the Reuters Institute for the Study of Journalism. 2019;.

21. Del Vicario M, Gaito S, Quattrociocchi W, Zignani M, Zollo F. News consumption during the Italian referendum: A cross-platform analysis on facebook and twitter. In: 2017 IEEE International Conference on Data Science and Advanced Analytics (DSAA). IEEE; 2017. p. 648–657.

22. Vicario MD, Quattrociocchi W, Scala A, Zollo F. Polarization and fake news: Early warning of Potential misinformation targets. ACM Transactions on the Web (TWEB). 2019;13(2):10.

23. Giglietto F, Iannelli L, Rossi L, Valeriani A, Righetti N, Carabini F, et al. Mapping Italian News Media Political Coverage in the Lead-Up to 2018 General Election. Available at SSRN 3179930. 2018;.

24. AGCOM. News vs Fake nel sistema dell'informazione; 2018.

25. Cantarella M, Fraccaroli N, Volpe R. Does Fake News Affect Voting Behaviour? 2019;.

26. Avaaz. Far Right Networks of Deception; 2019.

27. Shao C, Ciampaglia GL, Flammini A, Menczer F. Hoaxy: A Platform for Tracking Online Misinformation. In: Proceedings of the 25th International Conference Companion on World Wide Web. WWW '16 Companion. Republic and Canton of Geneva, Switzerland: International World Wide Web Conferences Steering Committee; 2016. p. 745–750. Available from: https://doi.org/10.1145/2872518.2890098.

28. Zollo F, Bessi A, Del Vicario M, Scala A, Caldarelli G, Shekhtman L, et al. Debunking in a world of tribes. PloS one. 2017;12(7):e0181821.

29. Rieder B. Studying Facebook via data extraction: the Netvizz application. In: Proceedings of the 5th annual ACM web science conference. ACM; 2013. p. 346–355.

30. Hagberg A, Swart P, S Chult D. Exploring network structure, dynamics, and function using NetworkX. Los Alamos National Lab.(LANL), Los Alamos, NM (United States); 2008.

31. Barabási AL. Network science. Cambridge university press; 2016.

32. Batagelj V, Zaversnik M. An O(m) algorithm for cores decomposition of networks. arXiv preprint cs/0310049. 2003;.

33. Fortunato S, Hric D. Community detection in networks: A user guide. Physics reports. 2016;659:1–44.

34. Blondel VD, Guillaume JL, Lambiotte R, Lefebvre E. Fast unfolding of communities in large networks. Journal of statistical mechanics: theory and experiment. 2008;2008(10):P10008.

35. Girvan M, Newman ME. Community structure in social and biological networks. Proceedings of the national academy of sciences. 2002;99(12):7821–7826.

36. Freeman LC. A set of measures of centrality based on betweenness. Sociometry. 1977; p. 35–41.

37. Page L, Brin S, Motwani R, Winograd T. The PageRank citation ranking: Bringing order to the web. Stanford InfoLab; 1999.

38. Mann HB. Nonparametric tests against trend. Econometrica: Journal of the Econometric Society. 1945; p. 245–259.

39. Kendall MG. Rank correlation methods. 1948;.

40. Efron B, Hastie T. Computer age statistical inference. vol. 5. Cambridge University Press; 2016.

41. Ratkiewicz J, Conover MD, Meiss M, Gonçalves B, Flammini A, Menczer FM. Detecting and tracking political abuse in social media. In: Fifth international AAAI conference on weblogs and social media; 2011.

42. McCombs ME, Shaw DL, Weaver DH. New directions in agenda-setting theory and research. Mass communication and society. 2014;17(6):781–802.

43. Vargo CJ, Guo L, Amazeen MA. The agenda-setting power of fake news: A big data analysis of the online media landscape from 2014 to 2016. New Media & Society. 2018;20(5):2028–2049. doi:10.1177/1461444817712086.

44. Horne BD, Adali S. This just in: fake news packs a lot in title, uses simpler, repetitive content in text body, more similar to satire than real news. arXiv preprint arXiv:170309398. 2017;.

45. Zajonc RB. Mere exposure: A gateway to the subliminal. Current directions in psychological science. 2001;10(6):224–228.

46. Blei DM, Ng AY, Jordan MI. Latent dirichlet allocation. Journal of machine Learning research. 2003;3(Jan):993–1022.

47. Wang P, Angarita R, Renna I. Is this the era of misinformation yet: combining social bots and fake news to deceive the masses. In: Companion Proceedings of the The Web Conference 2018. International World Wide Web Conferences Steering Committee; 2018. p. 1557–1561.

48. Amnesty I. Il Barometro dell'odio - Elezioni europee 2019; 2019.

49. Conti N. Elezioni europee, ma poca Europa. La Repubblica. 2019;.

50. FactCheckEU. Good news and bad news after election week-end. 2019;.

51. McCombs M. Setting the agenda: Mass media and public opinion. John Wiley & Sons; 2018.

52. Davis CA, Varol O, Ferrara E, Flammini A, Menczer F. Botornot: A system to evaluate social bots. In: Proceedings of the 25th International Conference Companion on World Wide Web. International World Wide Web Conferences Steering Committee; 2016. p. 273–274.

Click here to access/download;Figure;Fig1.tiff

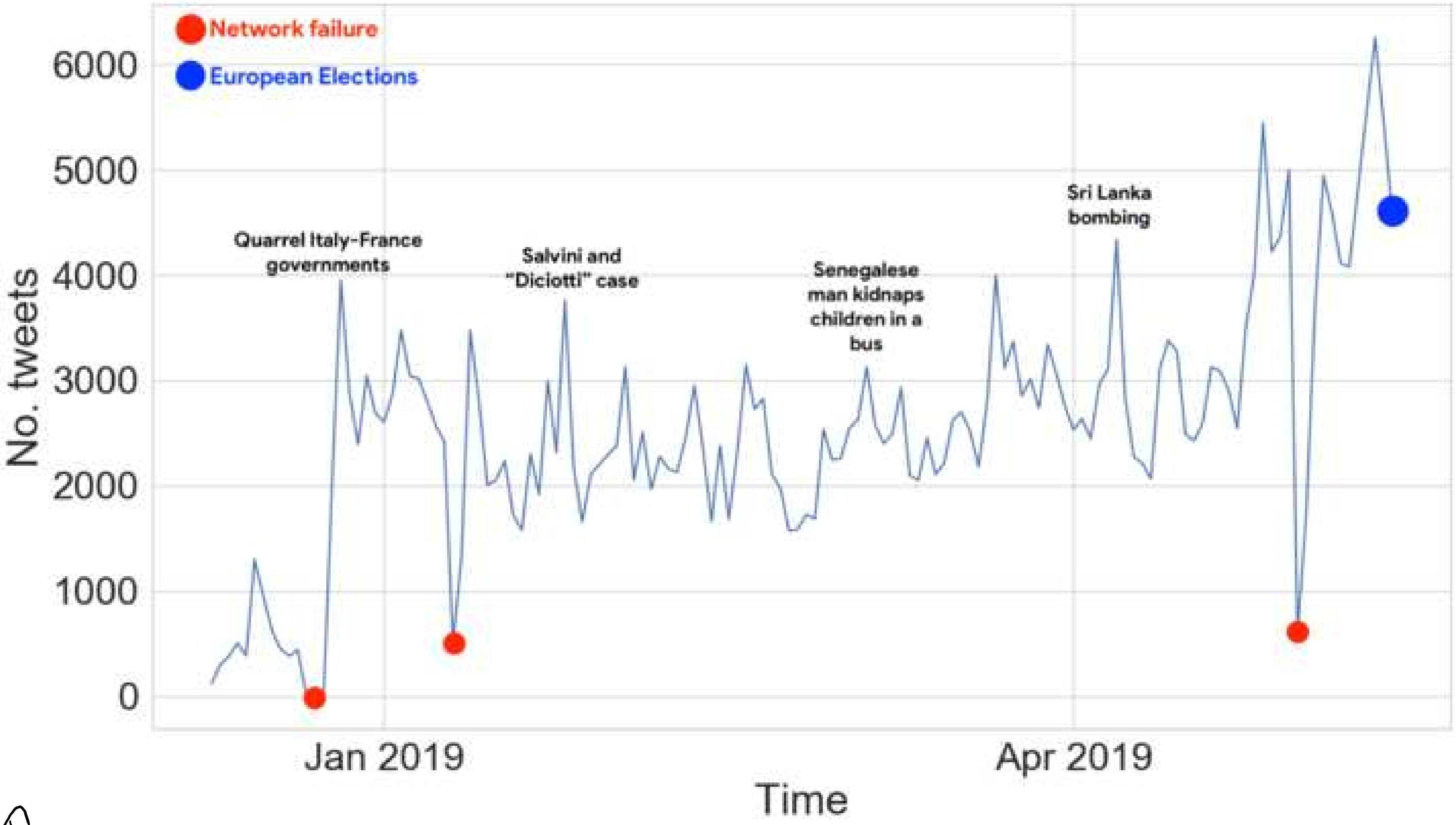

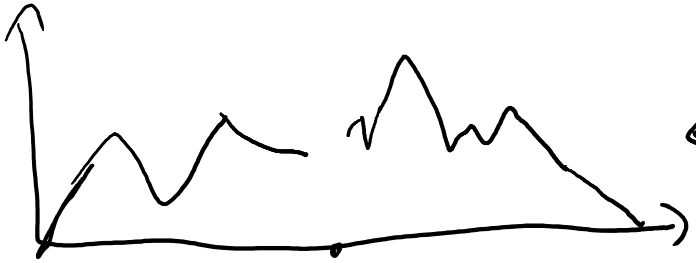

This was what I meant as alternative way of representing data gaps.

Click here to access/download;Figure;Fig2.tiff

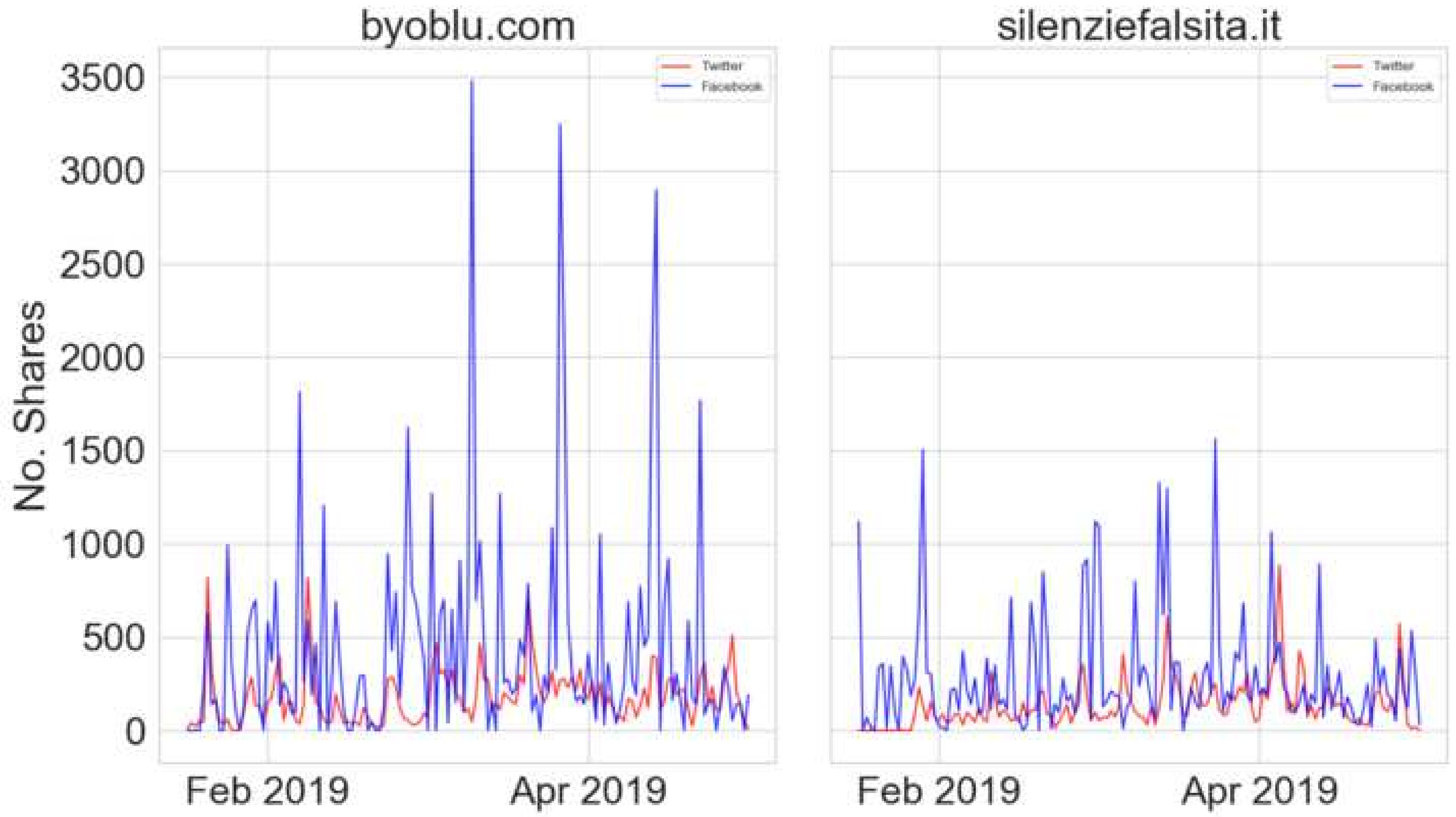

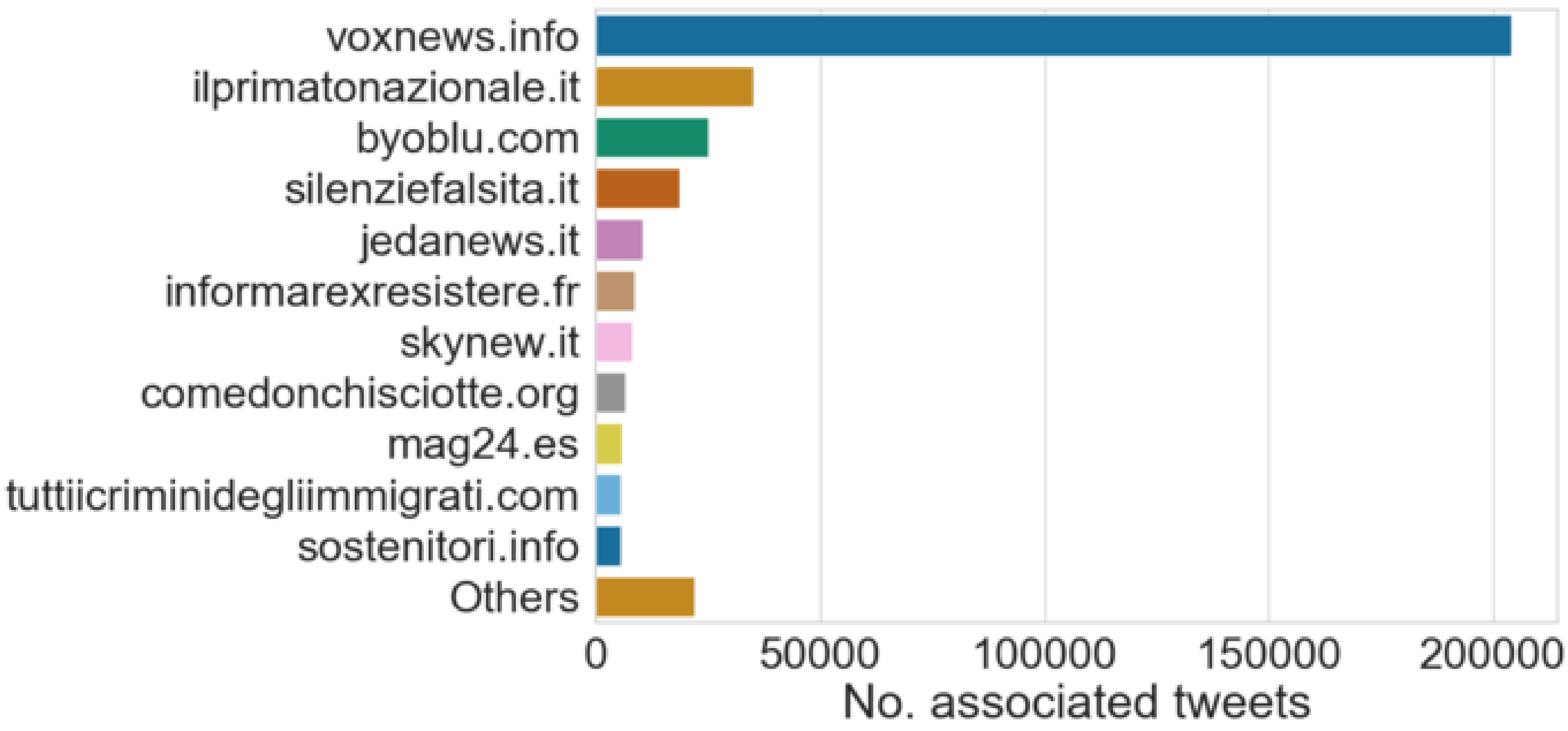
voxnews.info
ilprimatonazionale.it
byoblu.com
silenziefalsita.it
jedanews.it
informarexresistere.fr
skynew.it
comedonchisciotte.org
mag24.es
tuttiicriminidegliimmigrati.com
sostenitori.info
Others
0
50000
100000
150000
200000
No. associated tweets

Click here to access/download;Figure;Fig4.tiff

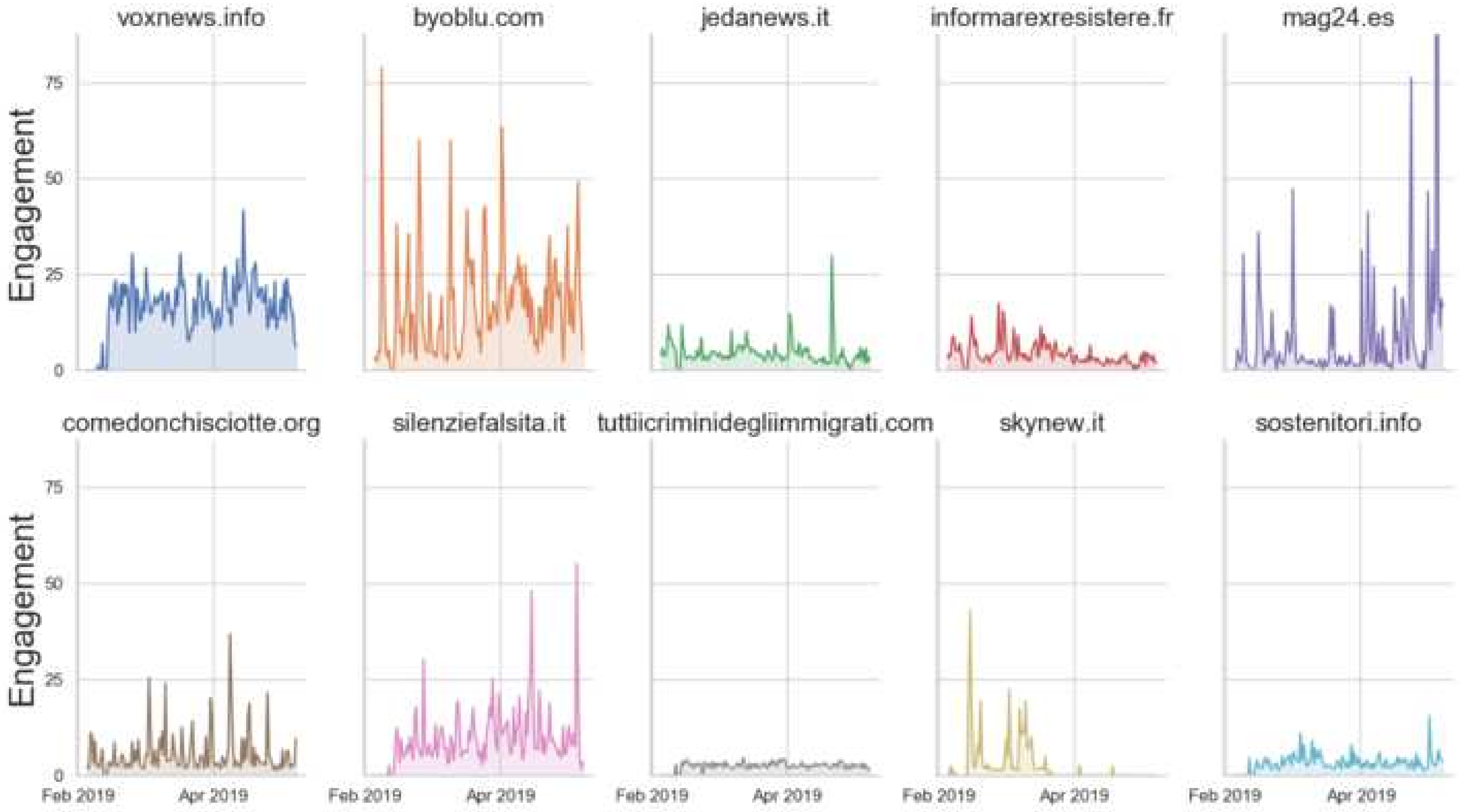

Click here to access/download;Figure;Fig6.tiff

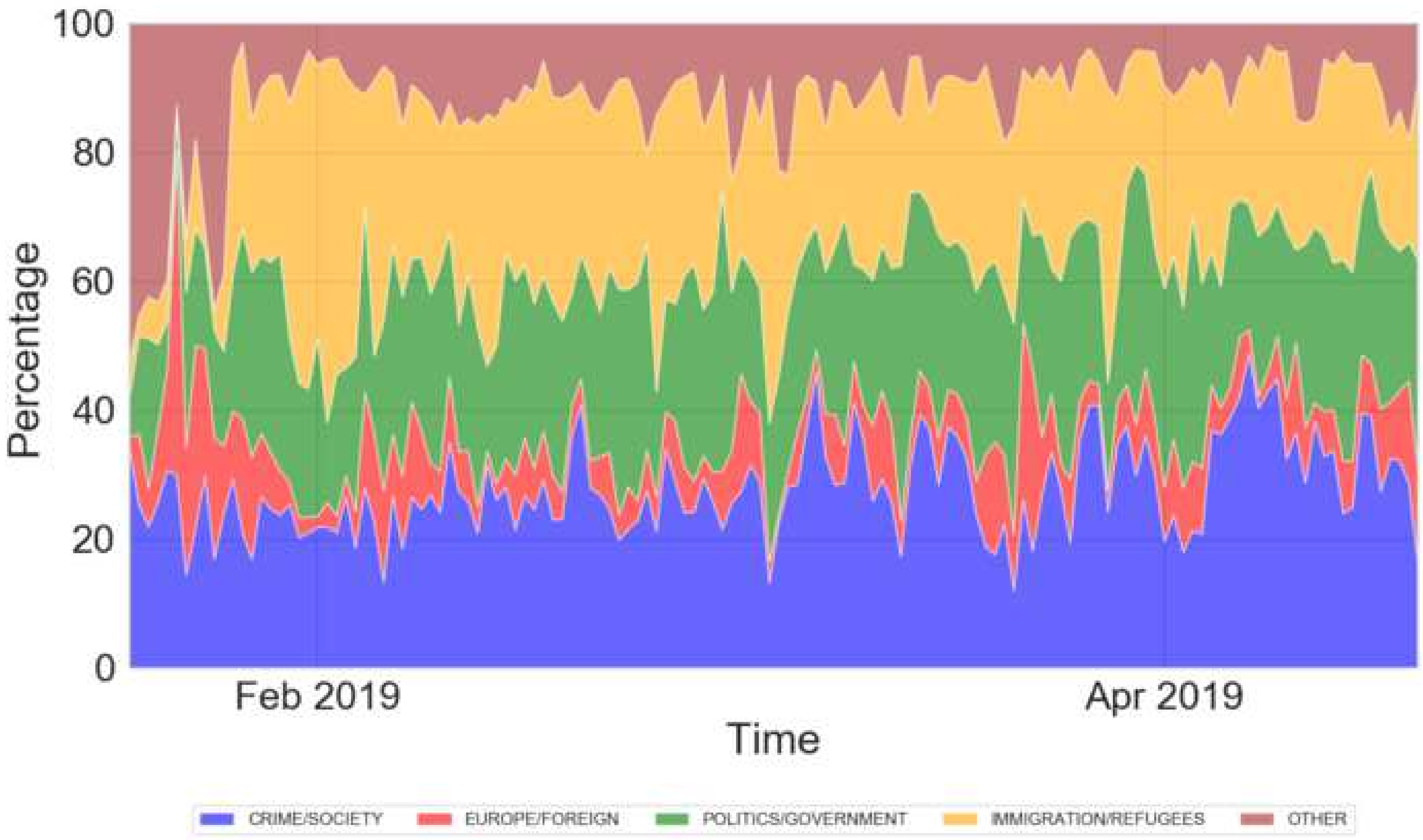

Click here to access/download;Figure;Fig7.tiff

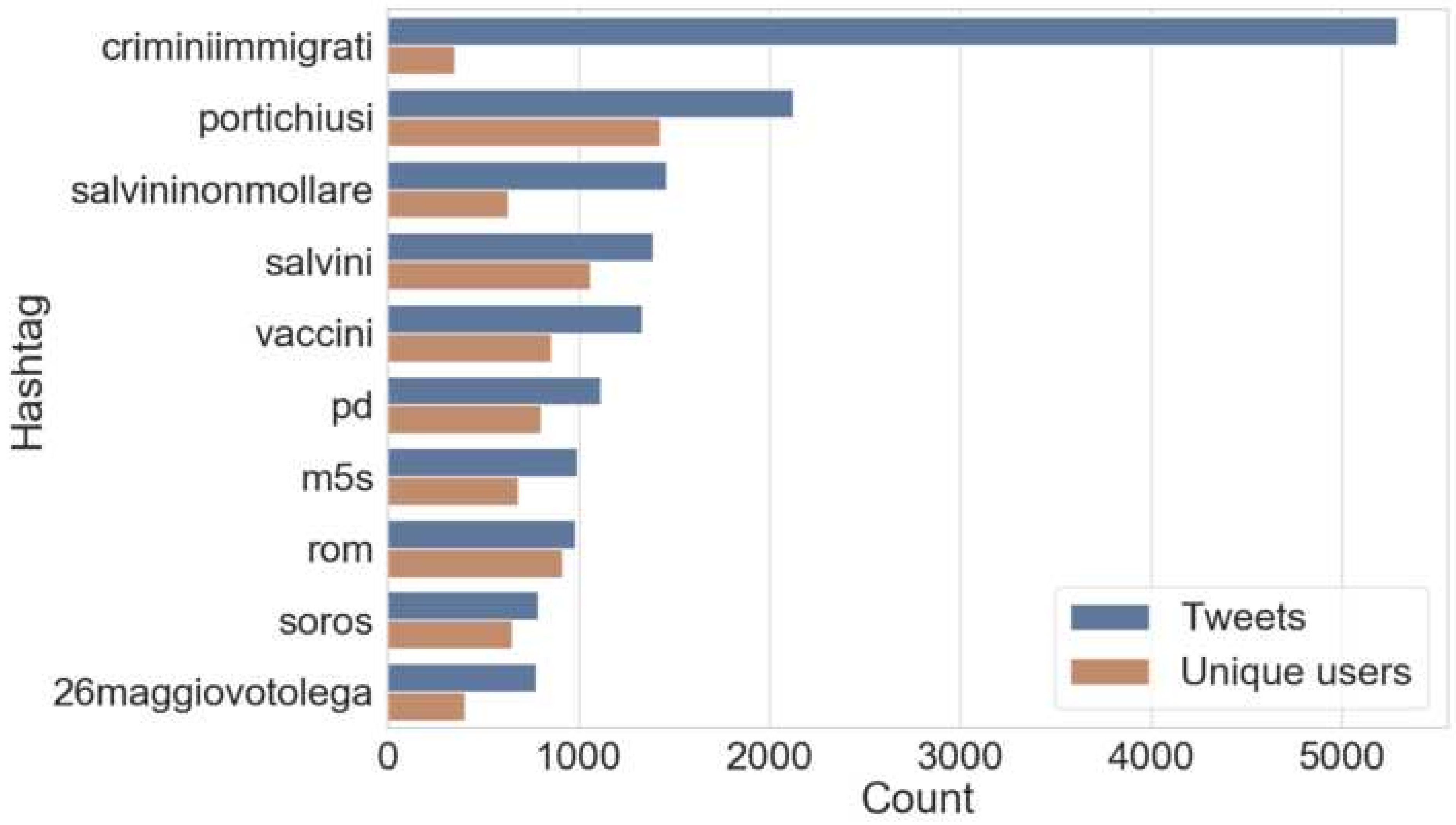

Click here to access/download;Figure;Fig8.tiff

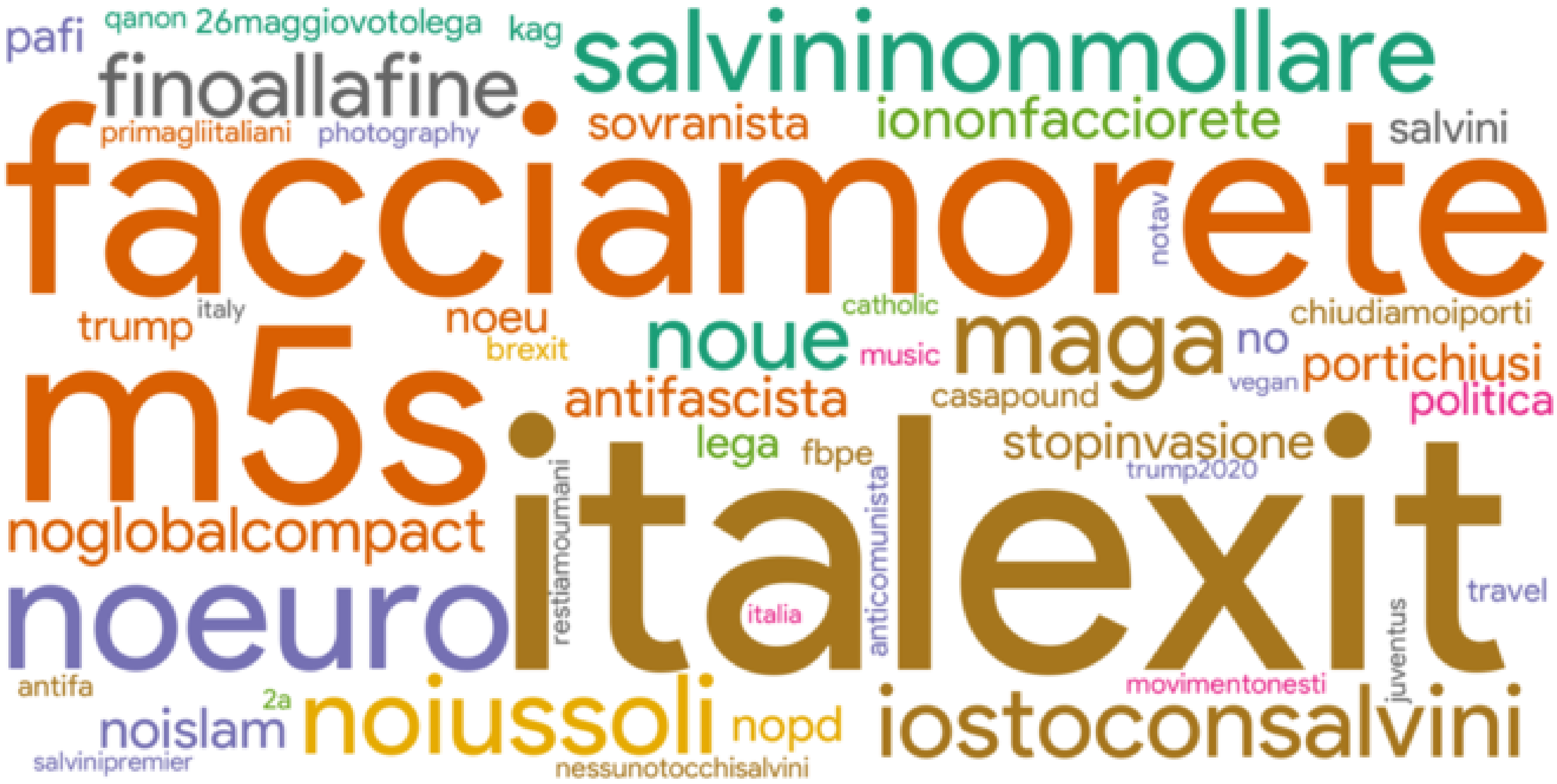

Click here to access/download;Figure;Fig9.tiff

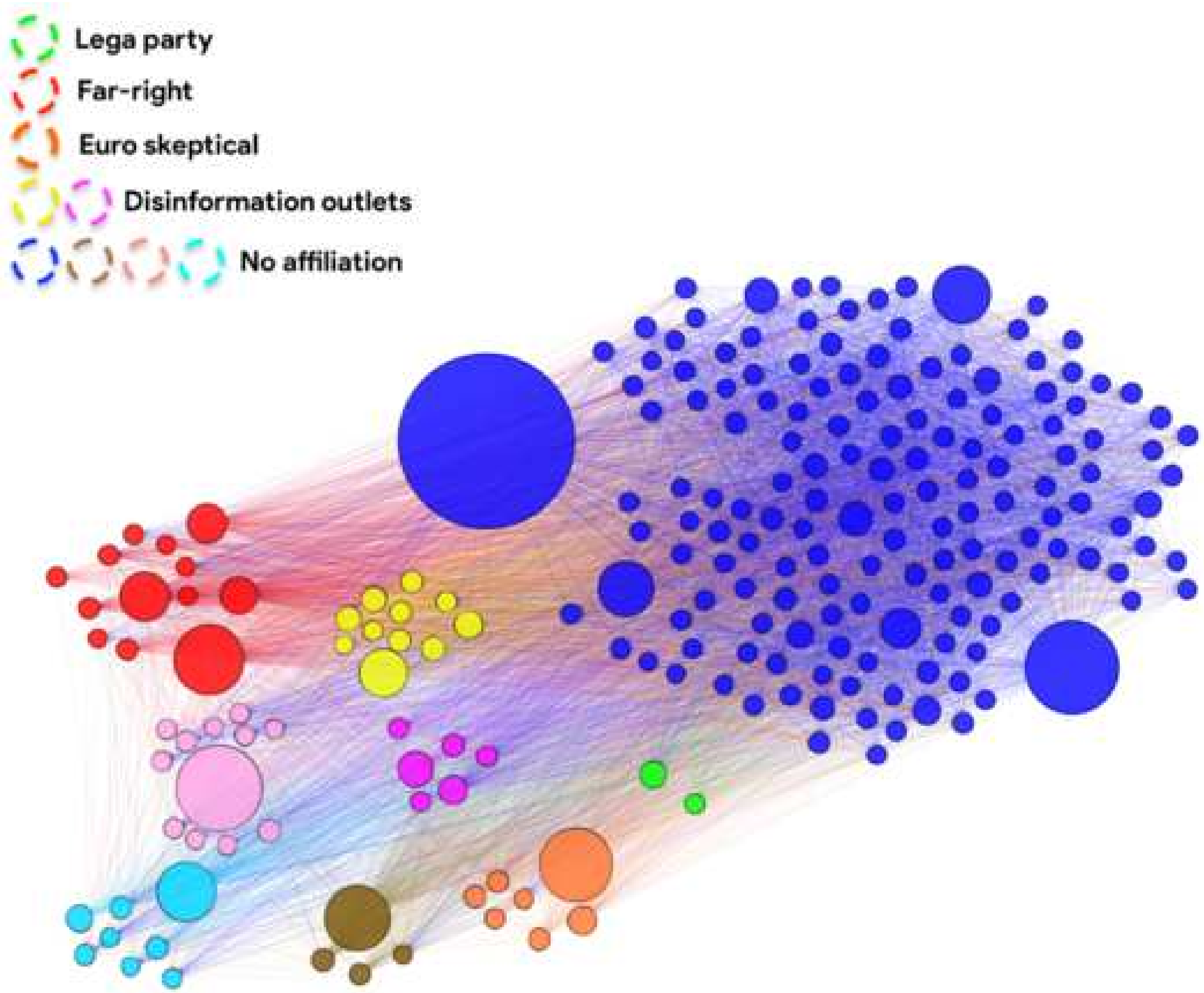

Click here to access/download;Figure;Fig10.tiff

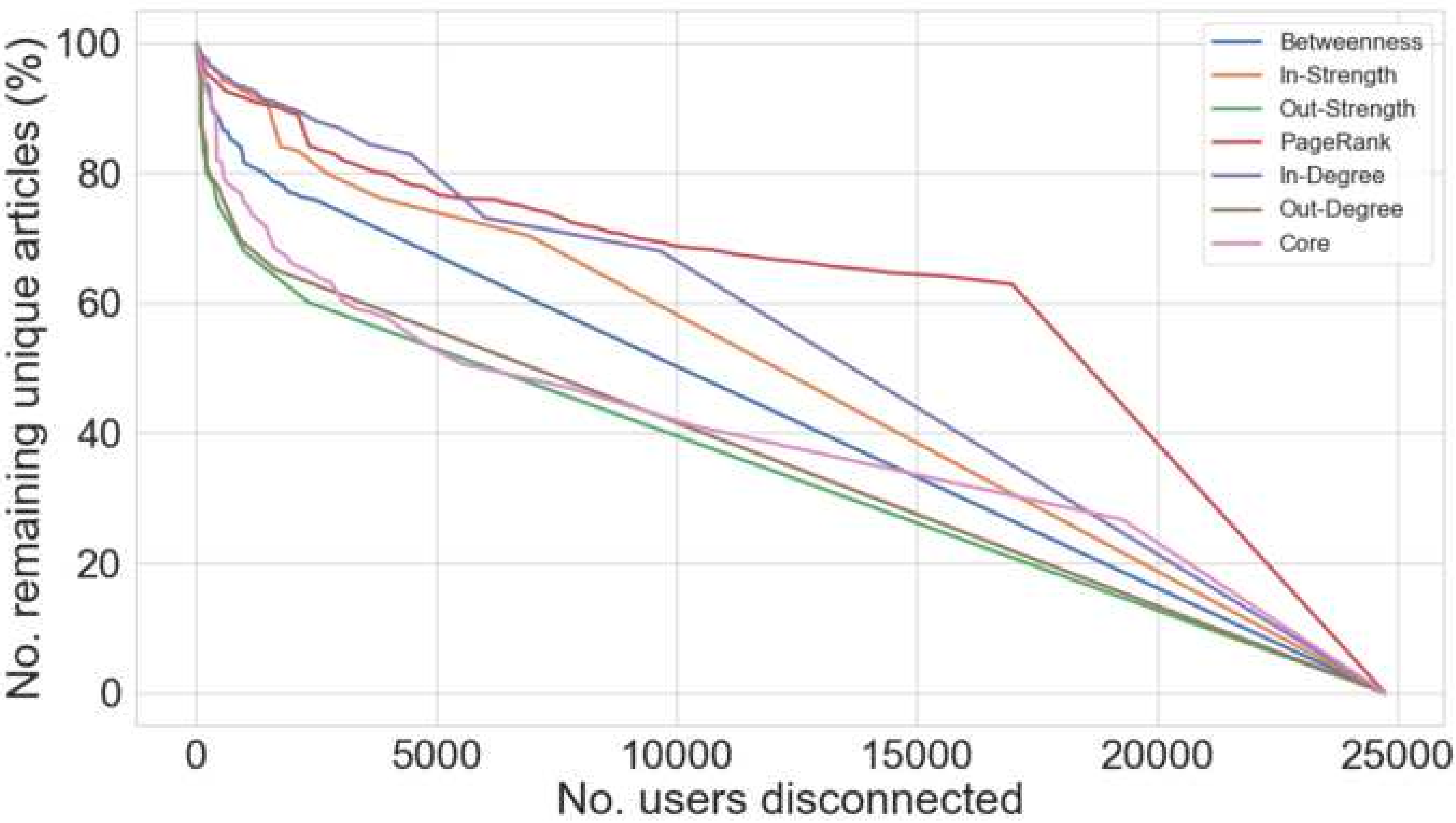

Click here to access/download;Figure;Fig11B.tiff

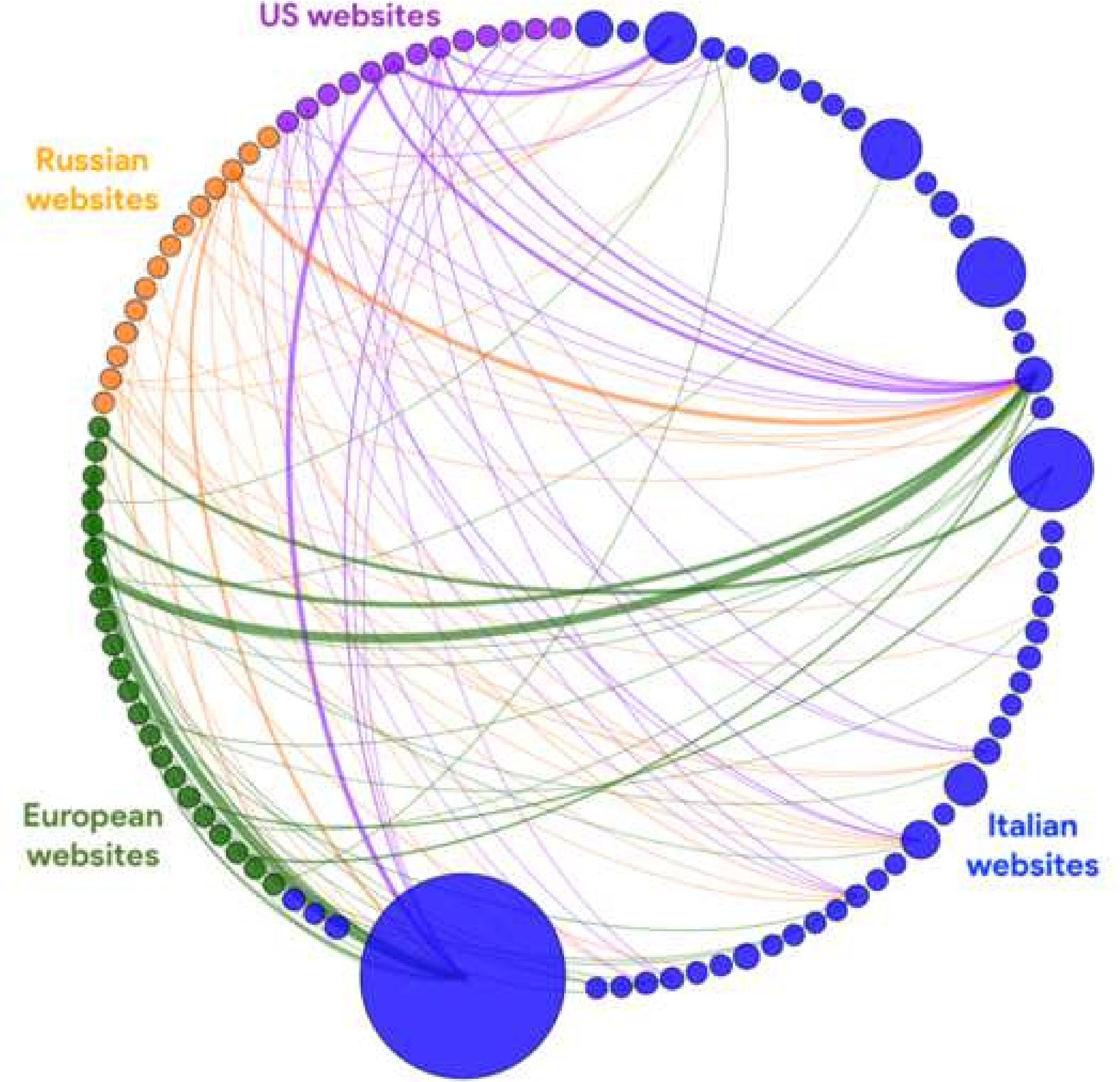

Click here to access/download;Figure;Fig11A.tiff

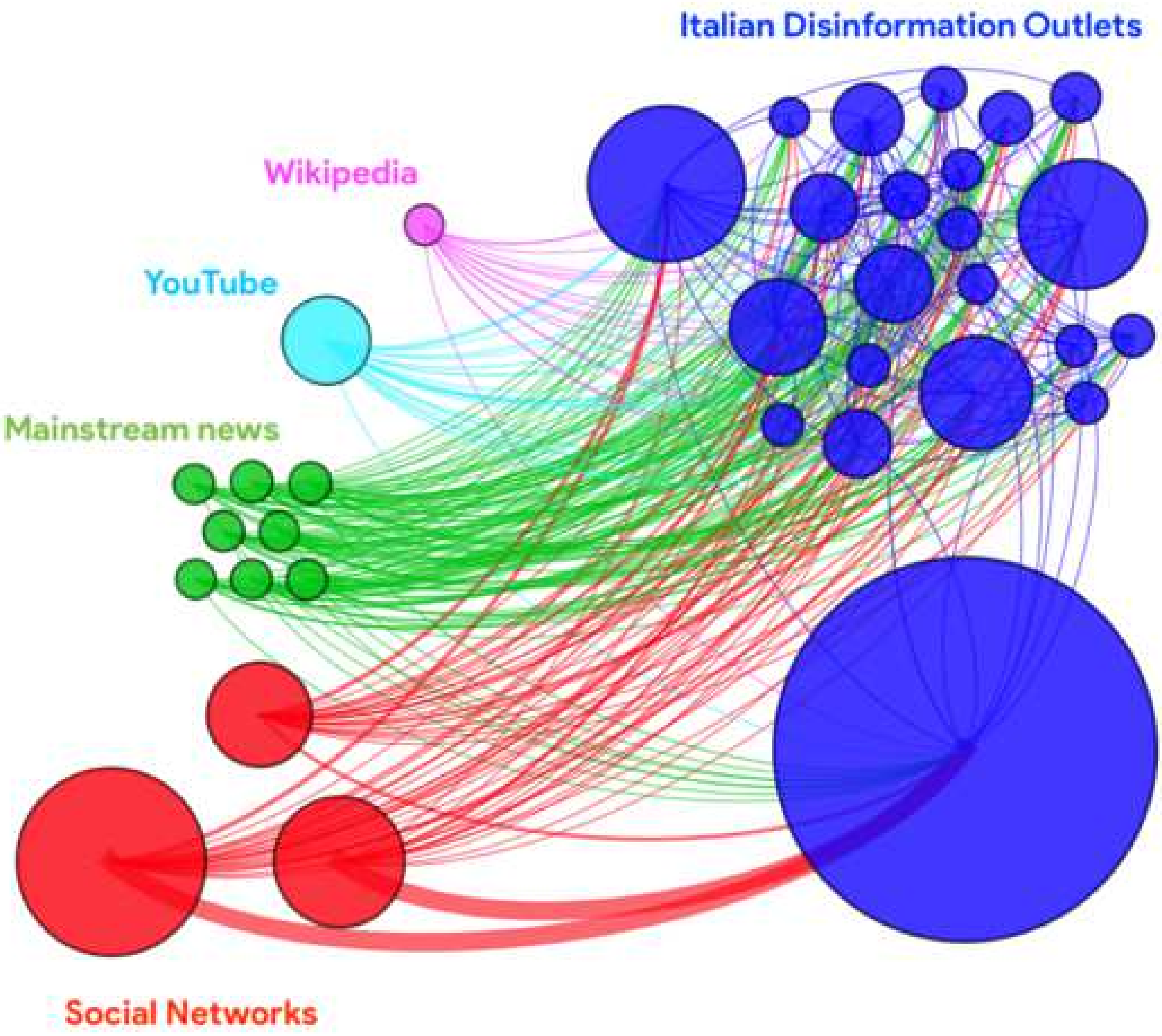

Click here to access/download;Figure;Fig12.tiff

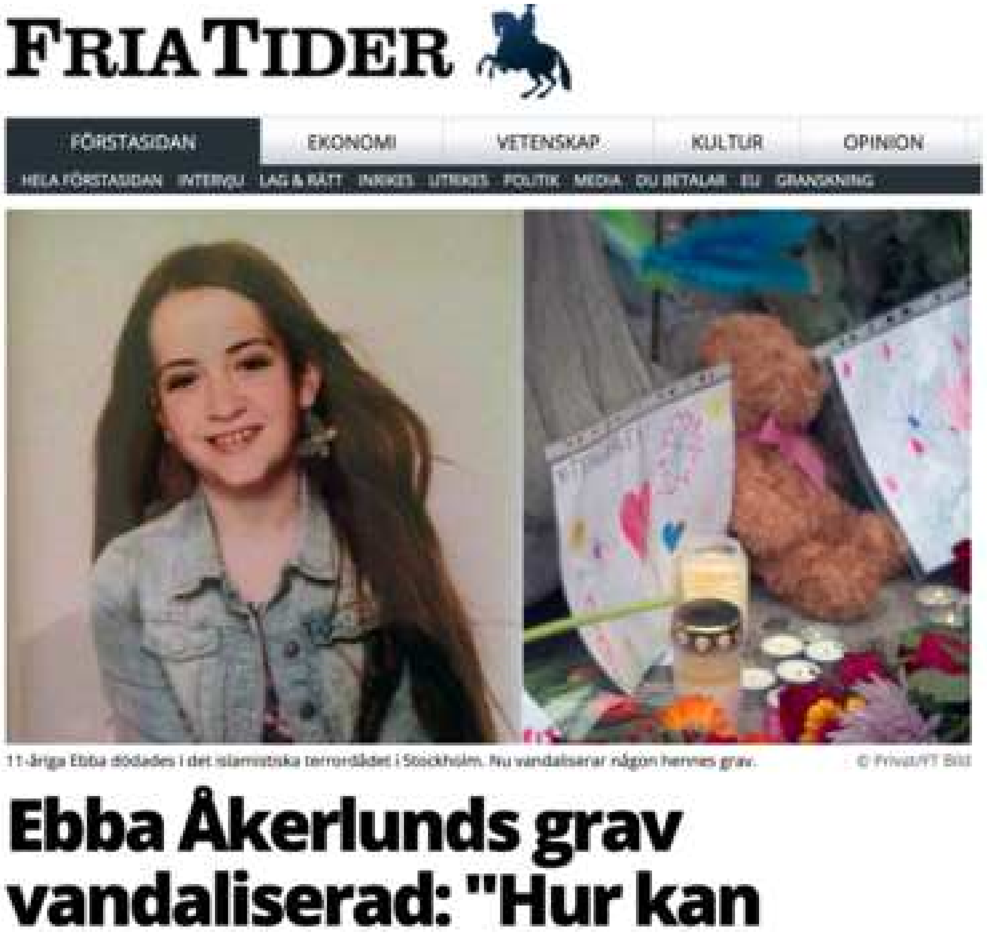

FRIATIDER

FÖRSTASIDAN | EKONOMI | VETENSKAP | KULTUR | OPINION

HELA FÖRSTASIDAN INTERVJU LAG & RÄTT INRIKES UTRIKES POLITIK MEDIA DU BETALAR EU GRANSKNING

11-åriga Ebba dödades i det islamistiska terrordådet i Stockholm. Nu vandaliserar någon hennes grav. © Privat/TT Bild

Ebba Åkerlunds grav vandaliserad: "Hur kan

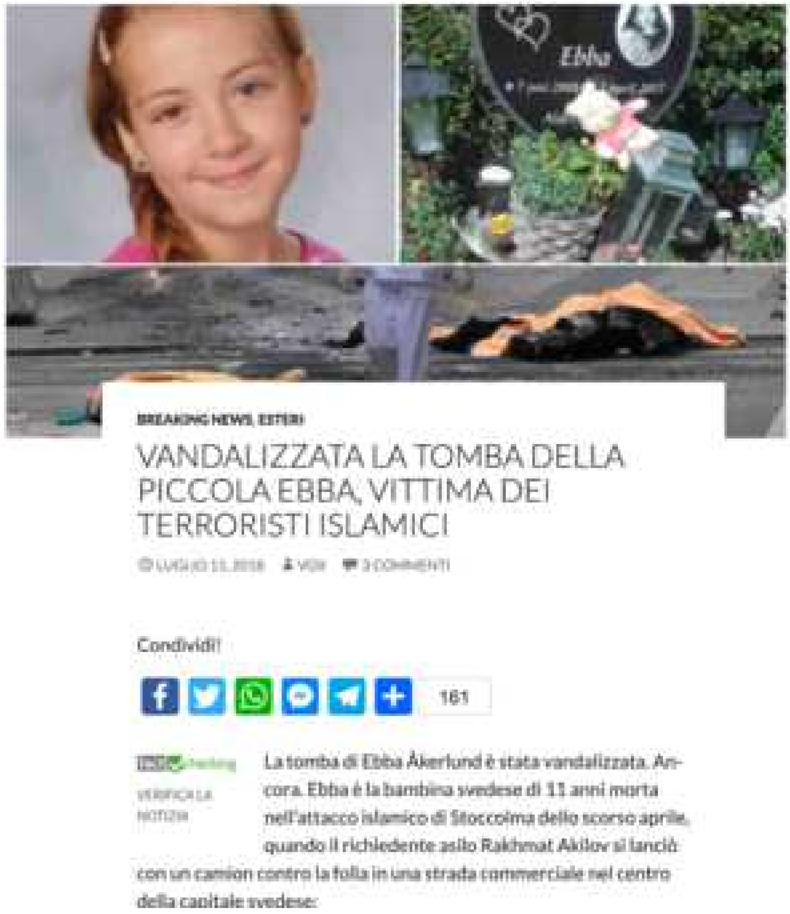

BREAKING NEWS, ESTERI

VANDALIZZATA LA TOMBA DELLA PICCOLA EBBA, VITTIMA DEI TERRORISTI ISLAMICI

LUGLIO 11, 2018 VOX 3 COMMENTI

Condividi!

161

VERIFICA LA NOTIZIA

La tomba di Ebba Åkerlund è stata vandalizzata. Ancora. Ebba è la bambina svedese di 11 anni morta nell'attacco islamico di Stoccolma dello scorso aprile, quando il richiedente asilo Rakhmat Akilov si lanciò con un camion contro la folla in una strada commerciale nel centro della capitale svedese:

Click here to access/download;Figure;Fig5B.tif

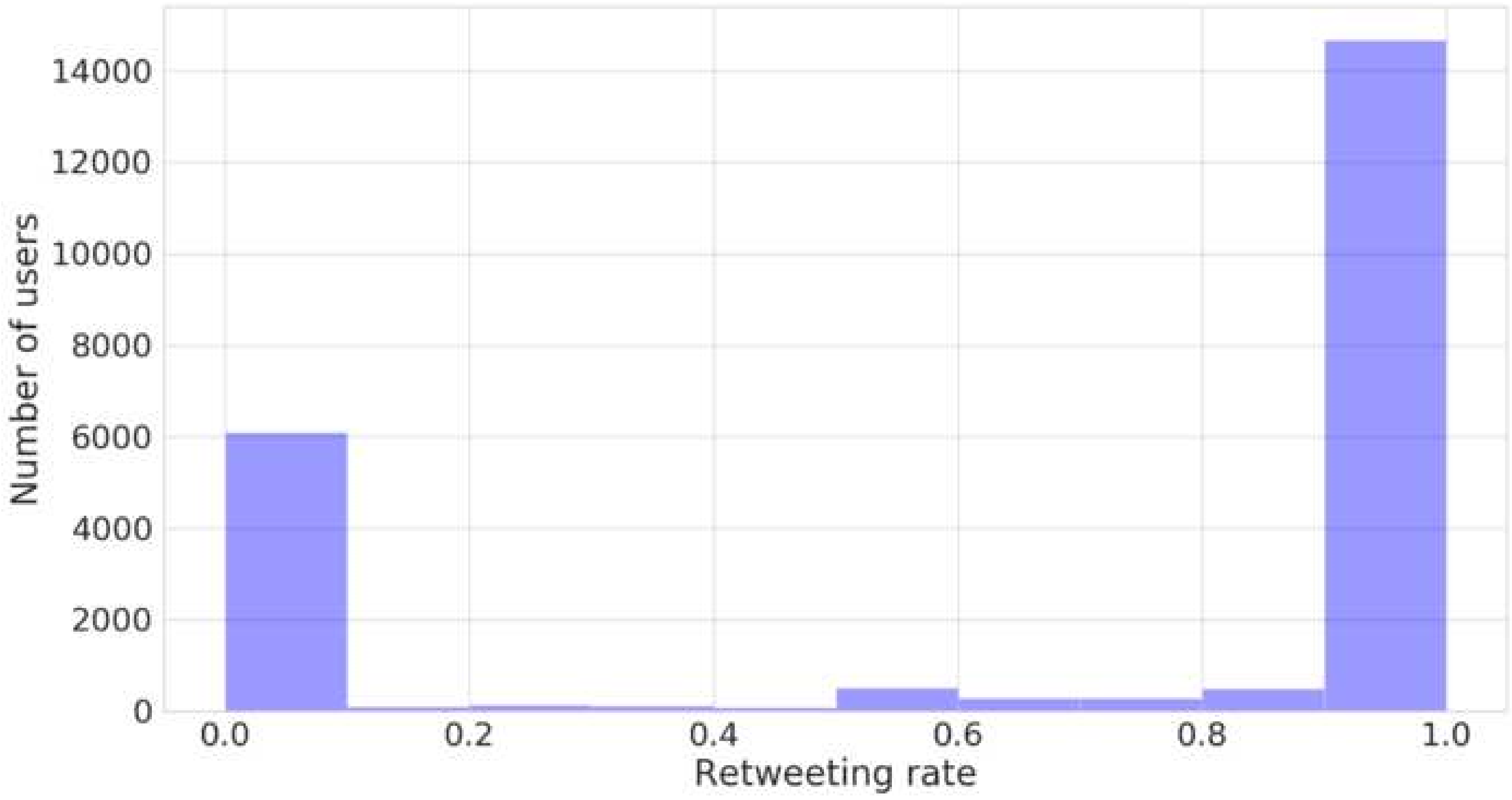

Click here to access/download;Figure;Fig5C.tiff

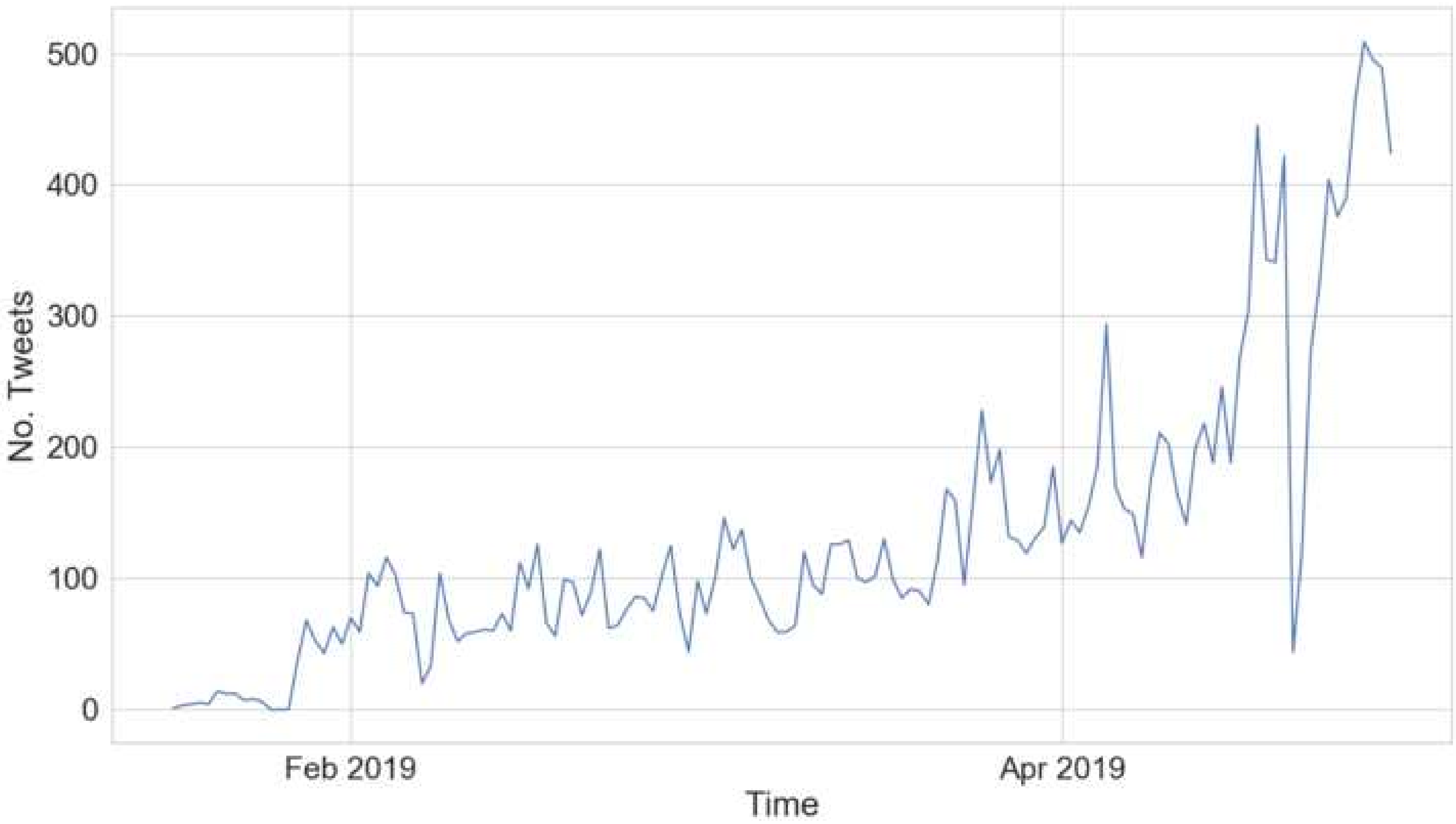

Click here to access/download;Figure;Fig5A.tiff

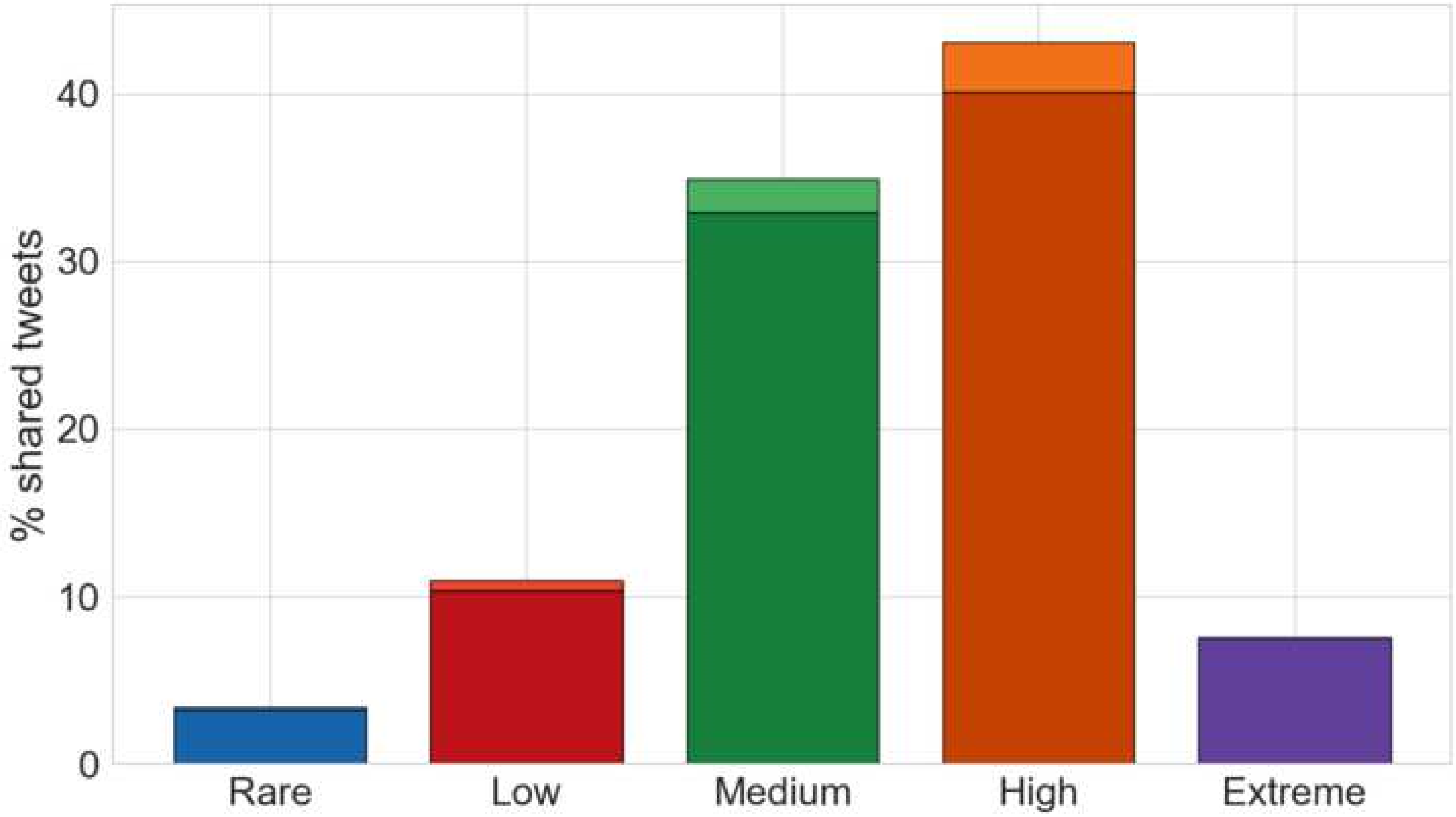

Click here to access/download;Figure;Fig3B.tiff

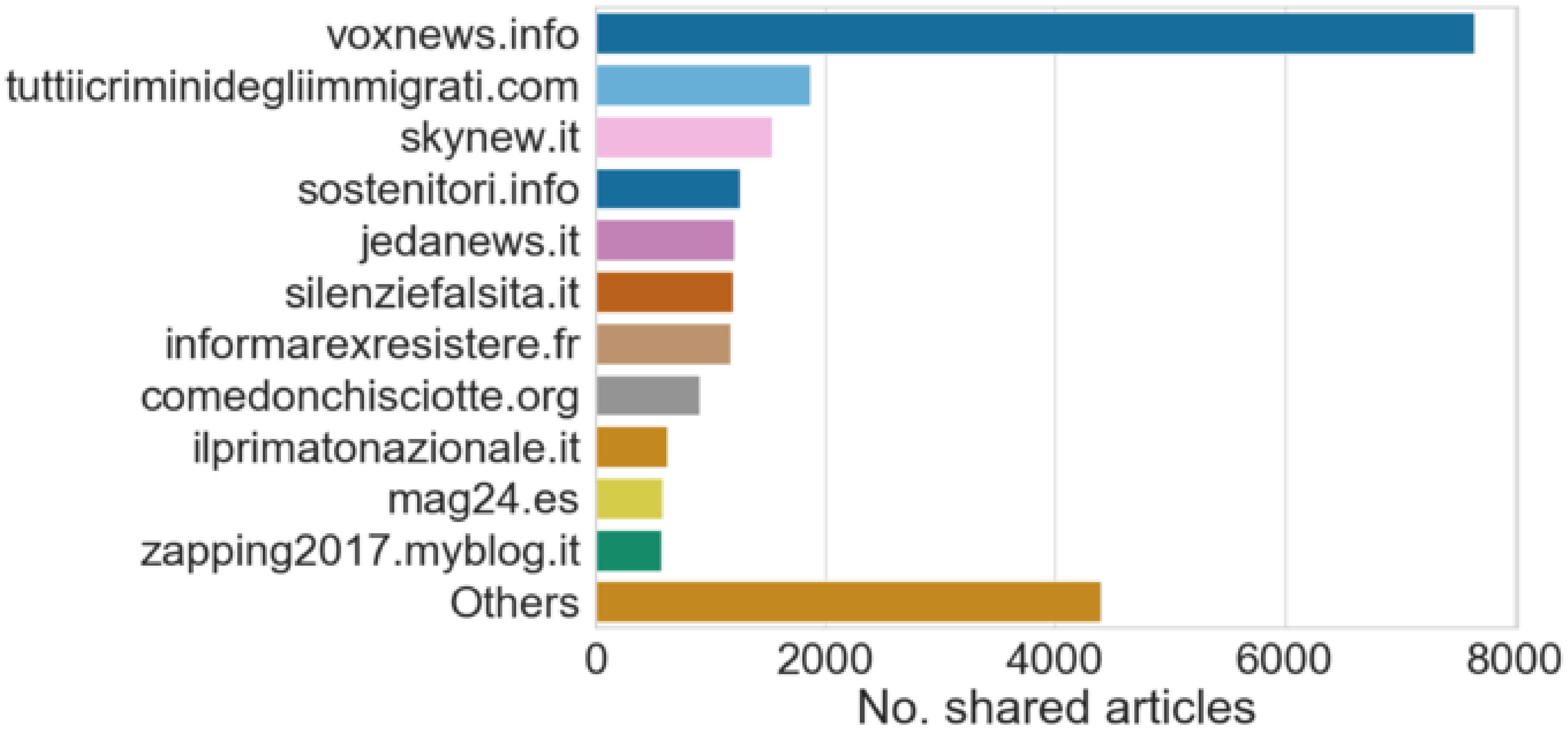

Revised Manuscript with Track Changes

Investigating Italian disinformation (spreading) on Twitter in the context of 2019 European elections

Francesco Pierri[1,*], Alessandro Artoni[1], Stefano Ceri[1]

1 Dept. of Electronics, Information and Bioengineering, Politecnico di Milano, 20133 Milano, Italy

*Corresponding author. E-mail: francesco.pierri@polimi.it

Check consistent terminology:
e.g theme/topic, fake/false/junk news,
disinformation/misinformation, etc

Abstract

We investigate the presence (and the influence) of disinformation spreading on online social networks in Italy, in the 5-month period preceding the 2019 European Parliament elections. To this aim we collected a large-scale dataset of tweets associated to thousands of news articles published on Italian disinformation websites. In the observation period, a few outlets accounted for most of the deceptive information circulating on Twitter, which focused on controversial and polarizing topics of debate such as immigration, national safety and (Italian) nationalism. We found evidence of connections between different disinformation outlets across Europe, U.S. and Russia, which often linked to each other and featured similar, even translated, articles in the period before the elections. Overall, the spread of disinformation on Twitter was confined in a limited community, strongly (and explicitly) related to the Italian conservative and far-right political environment, who had a limited impact on online discussions on the up-coming elections.

Better!

Introduction

In recent times, growing concern has risen over the presence and the influence of deceptive information spreading on social media [1]. The research community has employed a variety of different terms to indicate the same issue, namely disinformation, misinformation, propaganda, junk news and false (or ”fake”) news.

As people are more and more suspicious towards traditional media coverage [2], news consumption has considerably shifted towards online social media; these exhibit unique characteristics which favored, among other things, the proliferation of low-credibility content and malicious information [1, 2]. Consequently, it has been questioned in many circumstances whether and to what extent disinformation news circulating on social platforms impacted on the outcomes of political votes [2–5].

Focusing on 2016 US Presidential elections, recent research has shown that false news spread deeper, faster and broader than reliable news [6], with social bots and echo chambers playing an important role in the diffusion of deceptive information [7, 8]. However, it has also been highlighted that disinformation only amounted to a negligible

fraction of online news [9–11], the majority of which were exposed to and shared by a restricted community of old and conservative leaning people, highly engaged with political news [9–11]. In spite of such small volumes, a study suggested that false news (and the alleged interference of Russian trolls) played an important role in the election of Donald Trump [2].

As the European Union (EU) failed to counter the debt crisis which took place since the end of 2009 (following 2008 financial crisis in the US), populist and anti-establishment movements slowly formed up against EU which was now seen as a purely bureaucratic elite [12]. After 2016 Brexit Referendum, several parties embodying these ideals gained a lot of consensus in political elections across different countries (e.g. Hungary, France, Austria, Italy), building their propaganda on a so-called principle of sovereignty, claiming authority over flexibility clauses (which had previously led to austerity policies) and the Schengen treaty, which allows free movement, even willing to leave the EU [13]. As Europeans were called to elect their new representatives at the European Parliament–between the 23rd and the 26th of May 2019–traditional parties, such as European People's Party (EPP), Socialists and Democrats (S&D) and Alliance of Liberals and Democrats for Europe (ALDE), opposed a more cohesive yet renewed vision of Europe. Eventually, the pro-European side prevailed on aforementioned disruptive forces in all countries, with the only exception of Italy where "Lega" amplified its consensus (33%) and instead "Movimento 5 Stelle" declined (18%). Outside of our scope, a change of government occurred during the Summer of 2019.

For what concerns misbehavior on social platforms in European countries, recent research has highlighted the impact and the influence of social bots and online disinformation in different circumstances, including 2016 Brexit [5], 2017 French Presidential Elections [4,14] and 2017 Catalan referendum [15]. A significant presence of junk news in online conversations concerning 2019 European elections has been recently reported across several countries [14,16–18]. The European Commission has itself raised concerns–since 2015 [19]–about the large exposure of citizens to disinformation, promoting an action plan to build capabilities and enforce cooperation between different member states. In anticipation of 2019 European Parliament elections, they sponsored an ad-hoc fact-checking portal (`www.factcheck.eu`) to debunk false claims relative to political topics, aggregating reports from several agencies across different countries.

For what concerns Italy, according to Reuters [20], trust in news is today particularly low (40% of people trust overall news most of the time, 23% trust news in social media most of the time), as result of a long-standing trend which is mainly due to the political polarization of mainstream news organizations and of the resulting partisan nature of Italian journalism. Previous research on online news consumption highlighted the existence of segregated communities [21] and explored the characteristics of polarizing and controversial topics which are traditionally prone to misinformation [22]. Remarkable exposure to online disinformation was highlighted by authors of [23], who exhaustively investigated online media coverage in the run-up to 2018 Italian General elections; in particular, the study observed a rising trend in the spread of malicious information, with a peak of interactions in correspondence with the Italian elections. This result was later substantiated in a report of the Italian Authority for Communications Guarantees (AGCOM) [24]. A very recent work [25] has collected electoral and socio-demographic data, relative to Trentino and South Tyrol regions, as to directly estimate the impact of fake news on the 2018 electoral outcomes, with a focus on the populist vote; this study argues that malicious information had a negligible and non-significant effect on the vote. Furthermore, a recent investigation by Avaaz [26] revealed the existence of a network of Facebook pages and fake accounts which spread low-credibility and inflammatory content–reaching over a million interactions–in explicit support of ”Lega”, ”Movimento 5 Stelle” about controversial themes such as immigration, national safety and anti-establishment. Those pages were eventually shut down by Facebook as violating the platform’s terms of use.

In this work we focus on the 5-month period preceding 2019 European elections; we carry out our research on a consolidated setting, described in [8, 27], for investigating the presence (and the impact) of disinformation in the Italian Twittersphere. We recognize that our analysis has a few inherent limitations: first, according to Reuters [20] Twitter is overtaken by far by other social platforms, accounting for only 8% of total users (with a decreasing trend) when it comes to consume news online compared to Instagram (13%), YouTube (25%), WhatsApp (27%) and Facebook (54%), which exhibit instead a rising trend. Second, these differences are even more accentuated when comparing with the U.S. scenario [24], the focus of most of recent research. However, other aforementioned social media offer today little opportunities to

researchers to conveniently analyze the spread of online information, given the limitations they impose on the acquisition of data and the different user experiences they offer. Our study sheds light on the Italian mechanisms of disinformation spreading, and thus the outcomes of the analysis indicate directions for future research in the field.

To collect relevant data, we manually curated a list of websites which have been flagged by fact-checking agencies for fabricating and spreading a variety of malicious information, namely inaccurate and misleading news reports, hyper-partisan and propaganda stories, hoaxes and conspiracy theories. Differently from [8], satire was excluded from the analysis. Following literature on the subject [3, 7, 9–11], we used a "source-based" approach, and assumed that all articles published on aforementioned outlets indeed carried deceptive information; nonetheless, we are aware that this might not be always true and reported cases of misinformation on mainstream outlets are not rare [3]. Our analysis was driven by the following research questions:

RQ1: What was the reach of disinformation which circulated on Twitter in the run-up to European Parliament elections? How active and strong was the community of users sharing disinformation?

RQ2: What were the most debated themes of disinformation? How much were they influenced by national vs European-scale topics?

RQ3: Who are the most influential spreaders of disinformation? Do they exhibit precise political affiliations? How could we dismantle the disinformation network?

RQ4: Did disinformation outlets organize their deceptive strategies in a coordinated manner? Can we identify inter-connections across different countries?

We first describe the data collection and the methodology employed to perform our analysis, then we discuss each of the aforementioned research questions, and finally we summarize our findings.

Fig 1. Time series for the number of tweets, containing links to disinformation articles, collected in the period from 07/01/2019 to 27/05/2019. We annotated it with some events of interest.

Methods

Data Collection

Following a consolidated strategy [7, 8, 27], we leveraged Twitter Streaming API in order to collect tweets containing an explicit Uniform Resource Locator (URL) associated to news articles shared on a set of Italian disinformation websites. As a matter of fact, using the standard streaming endpoint allows to gather 100% of shared tweets matching the defined query (see next) [27].

next what?

To this aim we manually compiled a list of 63 disinformation websites that were still active in January 2019. We relied on blacklists curated by local fact-checking organizations (such as "butac.net", "bufale.net" and "pagellapolitica.it"); these include websites and blogs which share hyper-partisan and conspiratorial news, hoaxes, pseudo-science and satire. We initially started with only a dozen of websites, and we successively added other sources; this did not alter the overall collection procedure.

For sake of comparison, we also included four Italian fact-checking and debunking agencies, namely "lavoce.info", "pagellapolitica.it", "butac.net", "bufale.org".

In accordance with current literature [6, 9–11, 27] we use a "source-based" approach: we do not verify each news article manually but we assign the *disinformation* label to all items published on websites labeled as such (the same holds for *fact-checking* articles).

In order to filter relevant tweets, we used all domains as query `filter` parameters (dropping "www", "https", etc) in the form "`byoblu com OR voxnews info OR ...`" as suggested by Twitter Developers guide (`https://developer.twitter.com`). We built a crawler to visit these websites and parse URLs as to extract article text and other metadata (published date, author, hyperlinks, etc). We handled URL duplicates by directly visiting hyperlinks and comparing the associated HTML content. We also extracted profile information and Twitter timelines for all users using Twitter API.

The collection of tweets containing disinformation (see Fig 1) and fact-checking articles was carried out continuously from January 1st (2019) to May 27th, the day after EU elections in Italy. We collected 16,867 disinformation articles shared over

Fig 2. Time series for the number of shares on both Twitter (red) and Facebook (blue) for two disinformation outlets, respectively "byoblu.com" (left) and "silenziefalsita.it" (right), in the period from 07/01/2019 to 27/05/2019.

354,746 tweets by 23,243 unique users, and 1,743 fact-checking posts shared over 23,215 tweets by 9814 unique users.

We can observe that, in general, articles devoted to debunk false claims were barely engaged, accounting only for 6% of the total volume of tweets spreading disinformation in the same period; such findings are comparable with the US scenario [8], and they are in accordance with the very low effectiveness of debunking strategies which is documented in [28]. We leave for future research an in-depth comparative analysis of diffusion networks pertaining to the two news domains.

The entire data is available at: `https://doi.org/10.7910/DVN/OQHLAJ`

Comparison with Facebook

In order to perform a rough estimate of the different reach of disinformation on Twitter compared to Facebook, we collected data relative to the latter platform regarding two disinformation outlets, namely "byoblu.com" and "silenziefalsita.it", which have an associated Facebook page and are among Top-3 prolific and engaged sources of malicious information (see Results).

We used `netvizz` [29] to collect statistics on the number of daily shares of Facebook posts published by aforementioned outlets, and we compared with the traffic observed on Twitter. As we can see in Fig 2, disinformation has a stronger reach on Facebook than Twitter, for both sources, throughout the observation period; this is also shown in other works [23, 24, 26], coherently with the Italian consumption of social news. An in-depth analysis of the Italian disinformation on Facebook would be required, but it needs suitable assistance from Facebook for what concerns the disinformation diffusion network.

Network analysis

Building Twitter diffusion network

We built a global diffusion network–corresponding to the union of all sharing cascades

associated to articles gathered in our dataset–following a consolidated strategy [7,8]. We considered different Twitter social interactions altogether and for each tweet we add nodes and edges differently according to the action(s) performed by users:

- **Tweet**: a basic tweet corresponds to originally authored content, and it thus identifies a single node (author).
- **Mention**: whenever a tweet of user a contains a mention to user b, we build an edge from the author a of the tweet to the mentioned account b.
- **Reply**: when user a replies to user b we build an edge from a to b.
- **Retweet**: when user a retweets another account b, we build an edge from b to a.
- **Quote**: when user a quotes user b the edges goes from b to a.

When processing tweets, we add a new node for users involved in aforementioned interactions whenever they are not present in the network. As a remark, a single tweet can contain simultaneously several actions and thus it can generate multiple nodes and edges. Finally, we consider edges to be weighted, where the weight corresponds to the number of times two users interacted via actions mentioned beforehand.

Building the network of websites

In order to investigate existing inter-connections among different disinformation websites, and to understand the nature of external sources which are usually mentioned by deceptive outlets, we searched for URLs in all articles present in our dataset, i.e. which were shared at least once on Twitter. We accordingly built a graph where each node is a distinct Top-Level Domain–the highest level in the hierarchical Domain Name System (DNS) of the Internet–and an edge is built between two nodes a and b whenever a has published at least an article containing an URL belonging to b domain; the weight of an edge corresponds to the number of shared tweets carrying an URL with an hyperlink from a to b. The final result is a directed weighted network of approximately 5k nodes and 8k edges. We used `networkx` Python package [30] to handle the network.

Main core decomposition, centrality measures and community detection

In our analysis we employed several techniques coming from the network science toolbox [31], namely k-core decomposition, community detection algorithms and centrality measures. We used `networkx` Python package to perform all the computations.

The k-core [32] of a graph G is the maximal connected sub-graph of G in which all vertices have degree at least k. Given the k-core, recursively removing all nodes with degree k allows to extract the $(k+1)$-core; the main core is the non-empty graph with maximum value of k. k-core decomposition can be employed as to uncover influential nodes in a social network [8].

Community detection is the task of identifying *communities* in a network, i.e. dense sub-graphs which are well separated from each other [33]. In this work we consider Louvain's fast greedy algorithm [34], which is an iterative procedure that maximizes the Newman-Girvan *modularity* [35]; this measure is based on randomizations of the original graph as to check how non-random the group structure is.

A centrality measure is an indicator that allows to quantify the importance of a node in a network. In a weighted directed network we can define the *In-strength* of a node as the sum of the weights on the incoming edges, and the *Out-strength* as the sum of the weights on the out-going edges. *Betweenness* centrality [36] instead quantifies the probability for a node to act as a bridge along the shortest path between two other nodes; it is computed as the sum of the fraction of all-pairs shortest paths that pass through the node. *PageRank* centrality [37] is traditionally used to rank webpages in search engine queries; it counts both the number and quality of links to a page to estimate the importance of a website, assuming that more important websites will likely receive more links from other websites.

Time series analysis

In our experiments, we carried out a trend analysis of time series concerning users' activity, topics contained in disinformation articles and the number of interconnections between different outlets.

In statistics, a trend analysis refers to the task of identifying a population

characteristic changing with another variable, usually time or spatial location. Trends can be increasing, decreasing, or periodic (cyclic). We used the Mann-Kendall statistical test [38, 39] as to determine whether a given time series showed a monotonic trend. The test is non-parametric and distribution-free, e.g. it does not make any assumption on the distribution of the data. The null hypothesis H_0, no monotonic trend, is tested against the alternative hypothesis H_a that there is either an upward or downward monotonic trend, i.e. the variable consistently increases or decreases through time; the trend may or may not be linear. We used `mkt` Python package.

The multiple testing (or large-scale testing) problem occurs when observing simultaneously a set of test statistics, to decide which if any of the null hypotheses to reject [40]. In this case it is desirable to have confidence level for the whole family of simultaneous tests, e.g. requiring a stricter significance value for each individual test. For a collection of null hypotheses we define the family-wise error rate (FWER) as the probability of making at least one false rejection, (at least one type I error). We used the classical *Bonferroni* correction to control the FWER at $\leq \alpha$ by strengthening the threshold of each individual testing, i.e. for an overall significance level α and N simultaneous tests, we reject the individual null hypothesis at significance level α/N.

Ethics statement

We do not need ethical approval as data was publicly available and collected through Twitter Streaming API; we do not infringe Twitter terms and conditions of use. The same holds for data relative to Facebook, which was obtained using `netvizz` application in accordance with their terms of service.

Results and discussion

Assessing the reach of Italian disinformation

Sources of disinformation

To understand the reach of different disinformation outlets, we first computed the distribution of the number of articles and tweets per source. We observed, as shown in Fig 3, that a few websites dominate on the remaining ones both in terms of activity and

Fig 3. A (Top). The distribution of the total number of shared articles per website. **B (Bottom).** The distribution of the total number of associated tweets per website. We show Top-11 (which account for over 95% of the total volume of tweets), and we aggregate remaining sources as ”Others”.

social audience.

In particular, with approximately 200k tweets (over 50% of the total volume) and 6k articles (about 1/3 of the total number), ”voxnews.info” stands out on all other sources; this outlet spreads disinformation spanning several subjects, from immigration to health-care and conspiratorial theories, and it runs campaigns against fact-checkers as well as labeling its articles with false ”fact-checking” labels as to deceive readers.

Interestingly, two other uppermost prolific sources such as ”skynew.it” and ”tuttiicriminidegliimmigrati.com” do not receive the same reception on the platform; the former has stopped its activity on March and the latter is literally–it translates as ”All the immigrants crimes”–a repository of true, false and mixed statements about immigrants who committed crimes in Italy.

We can also recognize three websites associated to public Facebook pages that have been recently banned after the investigation of Avaaz NGO, namely ”jedanews.it”, ”catenaumana.it” and ”mag24.es”, as they were ”regularly spreading fake news and hate speech in Italy” violating the platform’s terms of use [26].

We further computed the distribution of the daily engagement (the ratio `no.articles published/no.tweets shared` per day) per each source, noticing that a few sources exhibit a considerable number of social interactions in spite of fewer associated tweets, compared to uppermost ”voxnews.info”. We show the time series for the daily engagement of Top-10 sources, which account for over 95% of total tweets, in Fig 4. We can notice in particular that ”byoblu.com” exhibits remarkable spikes of engagement w.r.t to a very small number of total tweets compared to other outlets, whereas ”mag24.es” shows a suspiciously large number of shares in the month preceding the elections (and after the release of Avaaz report).

We excluded ”ilprimatonazionale.it” from this analysis as it was added only at the end of April (we collected around 30k associated tweets and less than 1000 articles); official magazine of ”CasaPound” (former) neo-fascist party–with style and agenda-setting that remind of Breitbart News–it exhibits a daily engagement of over 200

Fig 4. Daily engagement for Top-10 sources (ranked according to the total number of shared tweets). The Mann-Kendall test (upward trend at significance level 0.005) was accepted only for "byoblu.com".

tweets, exceeding all other websites .

As elections approached, we were interested to understand whether there were particular trends in the daily reception of different sources. Focusing on Top-10 sources (except "ilprimatonazionale.it") we performed a Mann-Kendall test to assess the presence of an upward or downward monotonic trend in the time series of (a) daily shared tweets and (b) daily engagement. Taking into account Bonferroni's correction, the test was rejected at $\alpha = 0.05/10 = 0.005$; both (a) and (b) exhibit an upward trend for "byoblu.com" alone, whereas the remaining sources are either stationary or monotonically decreasing. As this outlet strongly supported euro-skeptical positions (and often gave visibility to many Italian representatives of such arguments) we argue that in the run-up to the European elections its agenda became slightly more captivating for the social audience.

User activity

For what concerns the underlying community of users sharing disinformation, we first computed the distribution of the number of shared tweets and unique URLs shared per number of users, noticing that a restricted community of users is responsible for spreading most of the online disinformation. In fact, approximately 20% of the community ($\sim$4k users) accounts for more than 90% of total tweets ($\sim$330k), in accordance with similar findings elsewhere [8–10]. Among them, we identified accounts officially associated to 18 different outlets (we manually looked at users' profile description and usernames); they overall shared 8310 tweets.

We also distinguished five classes of users based on their generic activity, i.e. the number of shared tweets containing an URL to disinformation articles: *Rare* (about 9.5k users) with only 1 tweet; *Low* (about 8k users) with more than 1 tweet and less than 10; *Medium* (about 3k users) with a number of tweets between 11 and 100; *High* (about 500 users) with more than 100 tweets but less than 1000; *Extreme* (exactly 20 users) with more than 1000 shared tweets. About 1 user out of 5 shared more than 10 disinformation articles in five months.

Fig 5. A (Top). A breakdown of the total volume of tweets according to the activity of users. Fractions of users created in the six months before the elections are indicated with lighter shades; these account respectively for 0.18% (*Rare*), 0.6% (*Low*), 2.04% (*Medium*) and 2.98% (*High*) of total tweets.
B (Center). The distribution of the number of users per retweeting activity.
C (Bottom). The distribution of daily tweets shared by recently created users.

As shown in Fig 5A, we can notice that a minority of very active users (the ensemble with *High* and *Extreme* activity) accounts for half of the deceptive stories that were shared, and over 3/4 of the total number of tweets was shared by less than 4 thousand users (*Medium*, *High* and *Extreme* activity).

We overall report 21,124 active (20 of which are also verified), 800 deleted, 124 protected and 112 suspended accounts. Verified accounts were altogether involved in 5761 tweets, only 18 of which in an "active" way, i.e. a verified account actually authored the tweet. We observed that they were mostly called in with the intent to mislead their followers, adding deceptive content on top of quoted statuses or replies.

Next we inspected the distribution of the number of users concerning their re-tweeting activity, i.e. the fraction of re-tweets compared to the number of pure tweets; as shown in Fig 5B this is strongly bi-modal, and it reveals that users sharing disinformation are mostly "re-tweeters": more than 60% of the accounts exhibit a re-tweeting activity larger than 0.95 and less than 30% have a re-tweeting activity smaller than 0.05. This shows that a restricted group of accounts is presumably responsible for conveying in the first place disinformation articles on the platform, which are propagated afterwards by the rest of the community.

We computed the distribution of some user profile features, namely the count of followers and friends, the number of statuses authored by users and the age on the social platform (in number of months passed since the creation date to May 2019). We report that users sharing disinformation tend to be quite "old" and active on the platform–with an average age of 3 years and more than a thousand authored statuses. We were able to gather information via Twitter API only for active and non-protected users.

We further inspected recently created accounts, noticing that approximately a thousand user was registered during the collection period, i.e. the last six months; they show similar distributions of aforementioned features compared to older users. Overall (see Fig 5B) they mostly pertain to active classes (*Medium* and *High*) and they account

for 15% (around 18k tweets) of the total volume of tweets considered–which lowers to approximately 288k tweets excluding those authored by non-active, suspended and protected accounts. Furthermore, about a hundred exhibit abnormal activities, producing more than 10k (generic) tweets in the period preceding the elections and directly sharing more than 10 disinformation stories each. We performed a Mann-Kendall test to the time series of daily tweets shared by such users (see Fig 5C), assessing the presence of a monotonically increasing trend (at significance level $\alpha = 0.05$). The main referenced source of disinformation is "voxnews.info" with more than 60% (circa 12k tweets) of the total number of shared stories. An activity of this kind is quite suspicious and could be further investigated as to detect the presence of "cyber-troops" (bots, cyborgs or trolls) that either attempted to drive public opinion in light of up-coming elections (via so-called "astroturfing" campaigns [41]) or simply redirected traffic as to generate online revenue through advertisement [1–3].

The agenda-setting of disinformation

Topic analysis

For what concerns the main themes covered by different disinformation outlets, relative to the resulting audience on Twitter, we based our analysis on the first level of agenda-setting theory [42], which states that news media set the public importance for objects based on the frequency in which these are mentioned and covered. In the case of fake news an agenda-setting effect could occur as a result of the rise in the coverage, even if some audience members are aware that fake news is fake [43]. We focused on the prevalence of titles, which were shared at least once, as they usually pack a lot of information about their claims in simple and repetitive structures [44]; besides, the exposure such as the presence alone of misleading titles on users' timelines could affect ordinary beliefs and result in a resistance to opposite arguments [28] and an increased perceived accuracy of the content, irrespective of its credibility [45].

We avoided automatic topic modeling algorithms [46] as they are not suitable for small texts. Therefore we carried out a topic analysis with a dictionary-based approach, and we manually compiled a list of keywords associated to five distinct topics namely: Politics/Government (PG), Immigration/Refugees (IR), Crime/Society (CS),

Fig 6. A stacked-area chart showing the distribution of different topics over the collection period. The daily coverage on themes related to Immigration/Refugees and Europe/Foreign is stationary, whereas focus on subjects related to Crime/Society and Politics/Government is monotonically increasing towards the elections (end of May 2019).

**Politics/Government**	**Immigration/Refugees**	**Europe/Foreign**	**Crime/Society**	**Other**
salvini	immigrati	euro	rom	video
italia	profughi	europa	milano	anni
pd	clandestini	ue	casa	contro
italiani	profugo	fusaro	bergoglio	foto
m5s	ong	diego	morti	vuole
italiana	porti	meluzzi	mafia	può
italiano	migranti	libia	bambini	vogliono
milioni	africani	macron	roma	parla
lega	immigrato	soros	donne	byoblu
sinistra	islamici	francia	bruciato	via
casapound	imam	francesi	confessa	niccolò
maio	seawatch	gilet	falsi	casal
soldi	nigeriani	gialli	bus	vero
guerra	nigeriana	europee	choc	ufficiale
cittadinanza	nigeriano	germania	figli	bufala
prima	islamica	tedesca	case	anti
raggi	africano	mondo	chiesa	sta
governo	stranieri	notre	famiglia	grazie
renzi	chiusi	dame	magistrato	casarini
zingaretti	sea	francese	polizia	farli

Table 1. Top-20 keywords associated with each topic.

Europe/Foreign (EF), Other (OT). Keywords were obtained with a data-driven approach, i.e. inspecting Top-500 most frequent words appearing in the titles, and taking into account relevant events that occurred in the last months. We provide Top-20 keywords for each topic in Table 1.

In particular, PG refers to main political parties and state government as well as the main political themes of debate. IR includes references to immigration, refugees and hospitality whereas CS includes terms mostly referring to crime, minorities and national security. Finally EF contains direct references to European elections and foreign countries. It is worth mentioning that the most frequent keyword was ”video”, suggesting that a remarkable fraction of disinformation was shared as multimedia content [47].

Do you have a reference to others using this method? The details are unclear to me.

We computed the relative presence of each topic in each article and accordingly assessed their distribution across tweets over different months. We can observe in Fig 6 that the discussion was stable on controversial topics such immigration, refugees, crime and government, whereas focus on European elections and foreign affairs was quite negligible throughout the period, with only a single spike of interest at the beginning of

Fig 7. Top-10 hashtags per number of shared tweets (blue) and unique users (orange).

January corresponding to the quarrel between Italian and France prime ministers. We also performed Mann-Kendall test to assess the presence of any monotonic trends in the daily distribution of different topics; we rejected the test for $\alpha = 0.05/5 = 0.01$ for IR and EF whereas we accepted it for the remaining topics, detecting the presence of an upward monotonic trend in CS and PG, and a downward monotonic trend in OT.

In the observation period, the disinformation agenda was well settled on main arguments supported by leading parties, namely ”Lega” and ”Movimento 5 Stelle”, since 2018 general elections; this suggests that they might have profited from and directly exploited hoaxes and misleading reports as to support their populist and nationalist views (whereas ”Partito Democratico” appeared among main targets of misinformation campaigns); empirical evidence for this phenomenon has been also widely reported elsewhere [23, 25]. However, the electoral outcome confirmed the decreasing trend of ”Movimento 5 Stelle” electoral consensus in favor of ”Lega”, which was rewarded with an unprecedented success.

Differently from 2018 [23] we in fact observed one main cited leader: Matteo Salvini (”Lega” party). This is consistent with a recent report on online hate speech [48], contributed by Amnesty International, which has shown that his activity (and reception) on Twitter and Facebook is 5 times higher than Luigi Di Maio (leader of ”Movimento 5 Stelle”); not surprisingly, his main agenda focuses (negatively) on immigration, refugees and Islam (which generated most of online interactions in 2018 [23]), which are also the main objects of hate speech and controversy in online conversations of Italian political representatives overall.

It appears that mainstream news actually disregarded European elections in the months preceding them, focusing on arguments of national debate [49]; this trend was also observed in other European countries according to FactCheckEU [50], claiming that misinformation was not prominent in online conversations mainly because European elections are not particularly polarized and are seen as less important compared to national elections. We believe that this might have affected the agenda of disinformation outlets, which are in general susceptible to traditional media coverage [51], thus explaining the focus on different targets in their deceptive strategies.

Fig 8. The cloud of words for Top-50 most frequent hashtags embedded in the users' profile description.

Usage of hashtags

Among most relevant hashtags shared along with tweets–in terms of number of tweets and unique users who used them (see Fig 7)–a few indicate main political parties (cf. "m5s", "pd", "lega") and others convey supporting messages for precise factions, mostly "Lega" (cf. "salvininonmollare", "26maggiovotolega"); some hashtags manifest instead active engagement in public debates which ignited on polarizing and controversial topics (such as immigrants hospitality, vaccines, the Romani community and George Soros). We also found explicit references to (former) far-right party "CasaPound" and the associated "Altaforte" publishing house, as well as some disinformation websites (with a remarkable polarization on "criminiimmigrati" which was shared more than 5000 times by only a few hundred accounts).

We also extracted hashtags directly embedded in the profile description of users collected in our data, for which we provide a cloud of words in Fig 8. The majority of them expresses extreme positions in matter of Europe and immigration: beside explicit references to "Lega" and "Movimento 5 Stelle", we primarily notice euro-skeptical (cf. "italexit", "noue"), anti-Islam (cf. "noislam") and anti-immigration positions (cf. "noiussoli", "chiudiamo i porti") and, surprisingly enough, also a few (alleged) Trump followers (cf. "maga" and "kag"). The latter finding is odd but somehow reflects the vicinity of Matteo Salvini and Donald Trump on several political matters (such as refugees and national security). On the other hand, we also notice "facciamorete", which refers to a Twitter grassroots anti-fascist and anti-racist movement that was born on December 2018, as a reaction to the recent policies in matter of immigration and national security of the Italian establishment.

Principal spreaders of disinformation

Central users in the main core

In order to identify most influential nodes in the diffusion network, we computed the value of several centrality measures for each account. We show in Table 2 the list of Top-10 users according to each centrality measure, and we also indicate whether they

Table 2. List of Top-10 users according to different centrality measures, namely In-strength, Out-Strength, Betweenness and PageRank; we indicate with a cross nodes that do not belong to the main K-core (k=47) of the network.

Rank	In-Strength	Out-Strength	Betweenness	PageRank
**1**	napolinordsud ×	Filomen30847137	IlPrimatoN	IlPrimatoN
**2**	RobertoPer1964	POPOLOdiTW1TTER	matteosalvinimi	matteosalvinimi
**3**	razorblack66	laperlaneranera	Filomen30847137	Sostenitori1 ×
**4**	polizianuovanaz ×	byoblu	byoblu	armidmar
**5**	Giulia46489464	IlPrimatoN	a_meluzzi	Conox_it ×
**6**	geokawa	petra_romano	AdryWebber	lauraboldrini ×
**7**	Gianmar26145917	araldoiustitia	claudioerpiu	pdnetwork ×
**8**	pasqualedimaria ×	max_ronchi	razorblack66	libreidee ×
**9**	il_brigante07	Fabio38437290	armidmar	byoblu
**10**	AngelaAnpoche	claudioerpiu	Sostenitori1 ×	Pontifex_it ×

belong or not to the main K-core of the network [32]; this corresponds to the sub-graph of neighboring nodes with degree greater or equal than $k = 47$, which is shown in Fig 9. We color nodes according to the communities identified by the Louvain modularity-based community algorithm [34] run on the original diffusion network (over 20k nodes and 100k edges).

Although we expect centrality measures to display small differences in their ranking, we can notice that the majority of nodes with highest values of In-Strength, Out-Strength and Betweenness centralities also belong to the main K-core of the network; the same does not hold for users which have a large PageRank centrality value. A few users strike the eye:

1. `matteosalvinimi` is Matteo Salvini, leader of the far-right wing ”Lega” party; he is not an active spreader of disinformation, being responsible for just one (true) story coming from disinformation outlet ”lettoquotidiano.com” (available at `https://twitter.com/matteosalvinimi/status/1102654128944308225`), which was shared over 1800 times. He is generally passively involved in deceptive strategies of malicious users who attempt to ”lure” his followers by attaching disinformation links in replies/re-tweets/mentions to his account.

2. `a_meluzzi` is Alessandro Meluzzi, a former representative of centre-right wing ”Forza Italia” party (whose leader is Silvio Berlusconi); he is a well-known supporter of conspiracy theories and a very active user in the disinformation network, with approximately 400 deceptive stories shared overall.

3. Accounts associated to disinformation outlets, namely `IlPrimatoN` with

Fig 9. The main K-core ($k = 47$) of the re-tweeting diffusion network. Colors correspond to different communities identified with the Louvain's algorithm. Node size depends on the total Strength (In + Out) and edge color is determined by the source node.

"ilprimatonazionale.it", `byoblu` with "byoblu.com", `libreidee` with "libreidee.org", `Sostenitori1` with "sostenitori.info" and `Conox_it` with "conoscenzealconfine.it".

A manual inspection revealed that most of the influential users are indeed actively involved in the spread of disinformation, with the only exception of `matteosalvinimi` who is rather manipulated by other users, via mentions/retweets/replies, as to mislead his huge community of followers (more than 2 millions). The story shared by Matteo Salvini underlines a common strategy of disinformation outlets identified in this analysis: they often publish simple true and factual news as to bait users and expose them to other harmful and misleading content present on the same website.

Besides, we recognized a few influential users who are (or have been in the past) target of several disinformation campaigns:

I'd like more on method here. Or did you literally just "recognize" some of the names? If so, I would suggest removing this section.

1. `lauraboldrini` is Laura Boldrini, representative of left-wing "Liberi e Uguali" party and actual member of the Italian Parliament; in the last few years she has been repetitively a target of fake news.

2. `pdnetwork` is the account of the centre-left "Partito Democratico" party; as the former ruling party it has been severely attacked in the propaganda of both actual "Lega" and "Movimento 5 Stelle".

3. `Pontifex_it` is the account of Papa Francesco; due to his recent statements showing empathy for migrants he has become another target of Italian far-right online hateful speech.

We also report a suspended account (`polizianuovanaz`), a protected one (`Giulia46489464`) and a deleted user (`pasqualedimaria`).

In addition, we investigated communities of users in the main K-core–which contains 218 nodes (see Fig 9)–and we noticed systematic interactions between distinct accounts. We manually inspected usernames, most frequent hashtags and referenced sources, deriving the following qualitative characterizations:

1. the **Green** community corresponds to "Lega" party official accounts: matteosalvinimi and legasalvini, whereas the third account, noipersalvini, belongs to the same community but does not appear in the core.

2. the **Red** community represents Italian far-right supporters, with several representatives of CasaPound (former) party (including his secretary distefanoTW who does not appear in the core), who obviously refer to "ilprimatonazionale.it" news outlet.

3. the **Yellow** community is strongly associated to two disinformation outlets, namely "silenziefalsita.it" (SilenzieFalsita) and "jedanews.it" (jedasupport); the latter was one of the pages identified in Avaaz report [26] and deleted by Facebook.

4. the **Orange** community is associated to the euro-skeptical and conspiratory outlet "byoblu.com" (byoblu), and it also features Antonio Maria Rinaldi (a_rinaldi), a well-known euro-skeptic economist who has just been elected with "Lega" in the European Parliament.

5. the **Purple** community corresponds to the community associated to "tuttiicriminidegliimmigrati.com" (TuttICrimin) disinformation outlet.

6. the remaining **Blue** (Filomen30847137), **Light-blue** (araldoiustitia) and **Brown** communities (petra_romano) represent different groups of very active "loose cannons" who do not exhibit a clear affiliation.

Eventually, we employed Botometer algorithm [52] as to detect the presence of social bots among users in the main core of the network. We set a threshold of 50% on the Complete Automation Probability (CAP)–i.e. the probability of an account to be completely automated–which, according to the authors, is a more conservative measure that takes into account an estimate of the overall presence of bots on the network; besides, we computed the CAP value based on the language independent features only, as the model includes also some features conceived for English-language users. We only detected two bot-like accounts, namely simonemassetti and jedanews, respectively with probabilities 58% and 64%, that belong to the same Purple community. A manual check confirmed that the former habitually shares random news content (also

Fig 10. Results of different network dismantling strategies w.r.t to remaining unique disinformation articles in the network. The x-axis indicates the number of disconnected accounts and the y-axis the fraction of remaining items in the network.

mainstream news) in an automatic flavour whereas the latter is the official spammer account of ”jedanews.it” disinformation outlet. We argue that the impact of automated accounts in the diffusion of malicious information is quite negligible compared to findings reported in [8], where about 25% of accounts in the main core of the US disinformation diffusion network were classified as bots.

Dismantling the disinformation network

Similar to [8], we performed an exercise of network dismantling analysis using different centrality measures, as to investigate possible intervention strategies that could prevent disinformation from spreading with the greatest effectiveness.

We first ranked nodes in decreasing order w.r.t to each metric, plus the core number–the largest k for which the node is present in the corresponding k-core–and the In and Out-degree, which exhibited the same Top 10 ranking as their weighted formulation (Strengths), but they do entail different results at dismantling the network. Next we delete them one by one while tracking the resulting fraction of remaining edges, tweets and unique articles in the network.

We observed that eliminating a few hundred nodes with largest values of Out-Degree promptly disconnects the network; in fact these users alone account for 90% of the total number of interactions between users. For what concerns the number of tweets sharing disinformation articles, the best strategy would be to target users with largest values of In-Strength who, according to our network representation, are likely to be users with a high re-tweeting activity; in fact, confirming previous observations, a few thousand nodes account for more than 75% of the total number of tweets shared in the five months before the elections. However, as shown in Fig 10, it is more challenging to prevent users to be exposed from even a tiny fraction of disinformation articles, as the network exhibits an almost linear relationship between the number of users disconnected and the corresponding number of remaining stories; as such the spread of malicious information would be completely prevented only blocking the entire network.

Fig 11. Two different views of the network of websites; the size of each node is adjusted w.r.t to the Out-strength, the color of edges is determined by the target node and the thickness depends on the weight (i.e. the number of shared tweets containing an article with that hyperlink).
A (Left). The main core of the network ($k = 14$); blue nodes are Italian disinformation websites, green ones are Italian traditional news outlets, red nodes are social networks, the sky-blue node is a video sharing website and the pink one is an online encyclopedia.
B (Right). The sub-graph of Russian (orange), EU (olive green), US (violet) and Italian (blue) disinformation outlets.

Coordinated strategies of deception

I'd like this modified in line with the abstract.

To investigate existing connections between different disinformation outlets and other external sources, we first analyzed the network of websites with a core decomposition [32], obtaining a main core ($k = 14$) which contains 35 nodes as a result of over 75,000 external re-directions via hyperlinks (shown in Fig 11A). Over 99% of the articles includes a hyperlink in the body. We may first notice frequent connections between distinct disinformation outlets, suggesting the presence of shared agendas and presumably coordinated deceptive tactics, as well as frequent mentions to reputable news websites; among them we distinguish ”IlFattoQuotidiano”, which is a historical supporter of ”Movimento 5 Stelle”, and conservative outlets such as ”IlGiornale” and ”LiberoQuotidiano” which lean instead towards ”Lega”. We also observe that most of the external re-directions point to social networks (Facebook and Twitter) and video sharing websites (Youtube); this is no wonder given that disinformation is often shared on social networks as multimedia content [1, 3]. In addition, we inspected nodes with the largest number of incoming edges (In-degree) in the original network, discovering among uppermost 20 nodes a few misleading reports originated on dubious websites (such as ”neoingegneria.com”), flagged by fact-checkers but that were not included in any blacklist. We believe that a more detailed network analysis could reveal additional relevant connections and we leave it for future research.

Furthermore, we focused on the sub-graph composed of three particular classes of nodes, namely Russian (RU) sources, EU/US disinformation websites and our list of Italian (IT) outlets; we manually identified notable Russian sources (”RussiaToday” and ”SputnikNews” networks) and we resorted to notable blacklists to spotlight other EU/US disinformation websites–namely ”opensources.co”, ”décodex.fr”, the list compiled by Hoaxy [27] and references to junk news in latest data memos by

COMPROP research group [14, 16–18].

The resulting bipartite network–we filtered out intra-edges between IT sources to better visualize connections with the ”outside” world–contains over 60 foreign websites (RU, US and EU) and it is shown in Fig 11B.

We observe a considerable number of external connections (over 500 distinct hyperlinks present in articles shared more than 5 thousand times) with other countries sources, which were primarily included within ”voxnews.info”, ”ilprimatonazionale.it” and ”jedanews.it”. Among foreign sources we encounter several well-known US sources (”breitbart.com”, ”naturalnews.com” and ”infowars.com” to mention a few) as well as RU (”rt.com”, ”sputniknews.com” and associated networks in several countries), but we also find interesting connections with notable disinformation outlets from France (”fdesouche.com” and ”breizh-info.com”), Germany (”tagesstimme.com), Spain (”latribunadeespana.com”) and even Sweden (”nyheteridag.se” and ”samnytt.se”). Besides, a manual inspection of a few articles revealed that stories often originated in one country were immediately translated and promoted from outlets in different countries (see Fig 12). Such findings suggest the existence of coordinated deceptive strategies which span across several countries, consistently with claims in latest report by Avaaz [26] which revealed the existence of a network of far-right and anti-EU websites, leading to the shutdown of hundreds of Facebook pages with more than 500 million views just ahead of the elections. Far-right disinformation tactics comprised the massive usage of fake and duplicate accounts, recycling followers and bait and switch of pages covering topics of popular interest (e.g. sport, fitness, beauty).

It is interesting that Facebook decided on the basis of external insights to shutdown pages delivering misleading content and hate speech; differently from the recent past [3, 7, 8] it might signal that social media are more willing to take action against the spread of deceptive information in coordination with findings from third-party researchers. Nevertheless, we argue that closing malicious pages is not sufficient and more proactive strategies should be followed [3, 26].

Finally, we performed a Mann-Kendall test to see whether there was an increasing trend, towards the elections, in the number of external connections with US and RU disinformation websites; we rejected it at $\alpha = 0.05/2 = 0.0025$.

Fig 12. An example of disinformation story who was published on a Swedish website ("friatider.se") and then reported by an Italian outlet ("voxnews.info"). Interestingly, this news is old (July 2018) but it was diffused again in the first months of 2019.

Conclusions

We studied the reach of Italian disinformation on Twitter for a period of five months immediately preceding the European elections **(RQ1)** by analyzing the content production of websites producing disinformation, and the characteristics of users sharing malicious items on the social platform. Overall, thousands of articles–which included hoaxes, propaganda, hyper-partisan and conspiratorial news–were shared in the period preceding the elections. We observed that a few outlets accounted for most of the deceptive information circulating on Twitter; among them, we also encountered a few websites which were recently banned from Facebook after violating the platform's terms of use. We identified a heterogeneous yet limited community of thousands of users who were responsible for sharing disinformation. The majority of the accounts (more than 75%) occasionally engaged with malicious content, sharing less than 10 stories each, whereas only a few hundred accounts were responsible for (the spreading) of thousands of articles (see Fig 5).

We singled out the most debated topics of disinformation **(RQ2)** by inspecting news items and Twitter hashtags. We observed that they mostly concern polarizing and controversial arguments of the local political debate such as immigration, crime and national safety, whereas discussion around the topics of Europe global management had a negligible presence throughout the collection period; the lack of European topics was also reported in the agenda of mainstream media.

Then we identified the most influential accounts in the diffusion network resulting from users sharing disinformation articles on Twitter **(RQ3)**, so as to detect the presence of active groups with precise political affiliations. We discovered strong ties with the Italian far-right and conservative community, in particular with "Lega" party, as most of the users manifested explicit support to the party agenda through the use of keywords and hashtags. Besides, a common deceptive strategy was to passively involve his leader Matteo Salvini via mentions, quotes and replies as to potentially mislead his audience of million of followers. We found limited evidence of bot activity in the main

core, and we observed that disabling a limited number of central users in the network would promptly interrupt to a certain extent the spread of disinformation circulating on Twitter, but it would immediately raise censorship concerns.

Finally, we investigated inter-connections within different deceptive agents **(RQ4)**, thereby observing that they repeatedly linked to each other websites during the period preceding the elections. Moreover we discovered many cases where the same (or similar) stories were shared in different languages across different European countries, as well as U.S. and Russia.

This analysis confirms that disinformation is present on Twitter and that its spread shows some peculiarities in terms of topics being discussed and of political affiliation of the key members of the information spreading community. We are aware that disinformation news in Italy have a higher share on Facebook than Twitter and that the use of Twitter in Italy as a social channel is limited compared to other social platforms such as Facebook, WhatsApp or Instagram. Therefore similar studies on other social media platforms will be needed and beneficial to our understanding of the spread of disinformation.

Acknowledgements

F.P. and S.C. are supported by the PRIN grant HOPE (FP6, Italian Ministry of Education). S.C. is partially supported by ERC Advanced Grant 693174. The authors are very grateful to Hoaxy support team at Bloomington Indiana University.

References

Check reference style! Some are missing journal or are in e.g. all-caps.

1. Pierri F, Ceri S. False news on social media: a data-driven survey. ACM SIGMOD Record Vol 48 Issue 2 (June). 2019;.
2. Allcott H, Gentzkow M. Social media and fake news in the 2016 election. Journal of Economic Perspectives. 2017;31(2):211–36.
3. Lazer DMJ, Baum MA, Benkler Y, Berinsky AJ, Greenhill KM, Menczer F, et al. The science of fake news. Science. 2018;359(6380):1094–1096. doi:10.1126/science.aao2998.

4. Ferrara E. Disinformation and social bot operations in the run up to the 2017 French presidential election. First Monday. 2017;22(8).

5. Bastos MT, Mercea D. The Brexit botnet and user-generated hyperpartisan news. Social Science Computer Review. 2019;37(1):38–54.

6. Vosoughi S, Roy D, Aral S. The spread of true and false news online. Science. 2018;359(6380):1146–1151. doi:10.1126/science.aap9559.

7. Shao C, Ciampaglia GL, Varol O, Yang KC, Flammini A, Menczer F. The spread of low-credibility content by social bots. Nature communications. 2018;9(1):4787.

8. Shao C, Hui PM, Wang L, Jiang X, Flammini A, Menczer F, et al. Anatomy of an online misinformation network. PLOS ONE. 2018;13(4):1–23. doi:10.1371/journal.pone.0196087.

9. Grinberg N, Joseph K, Friedland L, Swire-Thompson B, Lazer D. Fake news on Twitter during the 2016 U.S. presidential election. Science. 2019;363(6425):374–378. doi:10.1126/science.aau2706.

10. Bovet A, Makse HA. Influence of fake news in Twitter during the 2016 US presidential election. Nature Communications. 2019;10(1):7. doi:10.1038/s41467-018-07761-2.

11. Pierri F, Piccardi C, Ceri S. Topology comparison of Twitter diffusion networks reliably reveals disinformation news. arXiv. 2019;.

12. Henley J. How populism emerged as an electoral force in Europe. The Guardian. 2018;.

13. Dennison S, Zerka P. THE 2019 EUROPEAN ELECTION: HOW ANTI-EUROPEANS PLAN TO WRECK EUROPE AND WHAT CAN BE DONE TO STOP IT. European council on foreign relations. 2019;.

14. Howard PN, Bradshaw S, Kollanyi B, Bolsolver G. Junk News and Bots during the French Presidential Election: What Are French Voters Sharing Over Twitter In Round Two?;.

15. Stella M, Ferrara E, De Domenico M. Bots increase exposure to negative and inflammatory content in online social systems. Proceedings of the National Academy of Sciences. 2018;115(49):12435–12440.

16. Hedman F, Sivnert F, Howard P. News and political information consumption in Sweden: Mapping the 2018 Swedish general election on Twitter; 2018.

17. Kollanyi B, Howard PN. Junk news and bots during the German parliamentary election: What are German voters sharing over Twitter; 2017.

18. Marchal N, Kollanyi B, Neudert LM, Howard PN. Junk News During the EU Parliamentary Elections: Lessons from a Seven-Language Study of Twitter and Facebook. 2019;.

19. Commission E. Tackling online disinformation;. Available from: `https://ec.europa.eu/digital-single-market/en/tackling-online-disinformation`.

20. Nielsen RK, Newman N, Fletcher R, Kalogeropoulos A. Reuters Institute Digital News Report 2019. Report of the Reuters Institute for the Study of Journalism. 2019;.

21. Del Vicario M, Gaito S, Quattrociocchi W, Zignani M, Zollo F. News consumption during the Italian referendum: A cross-platform analysis on facebook and twitter. In: 2017 IEEE International Conference on Data Science and Advanced Analytics (DSAA). IEEE; 2017. p. 648–657.

22. Vicario MD, Quattrociocchi W, Scala A, Zollo F. Polarization and fake news: Early warning of Potential misinformation targets. ACM Transactions on the Web (TWEB). 2019;13(2):10.

23. Giglietto F, Iannelli L, Rossi L, Valeriani A, Righetti N, Carabini F, et al. Mapping Italian News Media Political Coverage in the Lead-Up to 2018 General Election. Available at SSRN 3179930. 2018;.

24. AGCOM. News vs Fake nel sistema dell'informazione; 2018.

25. Cantarella M, Fraccaroli N, Volpe R. Does Fake News Affect Voting Behaviour? 2019;.

26. Avaaz. Far Right Networks of Deception; 2019. ← e.g URL?

27. Shao C, Ciampaglia GL, Flammini A, Menczer F. Hoaxy: A Platform for Tracking Online Misinformation. In: Proceedings of the 25th International Conference Companion on World Wide Web. WWW '16 Companion. Republic and Canton of Geneva, Switzerland: International World Wide Web Conferences Steering Committee; 2016. p. 745–750. Available from: https://doi.org/10.1145/2872518.2890098.

28. Zollo F, Bessi A, Del Vicario M, Scala A, Caldarelli G, Shekhtman L, et al. Debunking in a world of tribes. PloS one. 2017;12(7):e0181821.

29. Rieder B. Studying Facebook via data extraction: the Netvizz application. In: Proceedings of the 5th annual ACM web science conference. ACM; 2013. p. 346–355.

30. Hagberg A, Swart P, S Chult D. Exploring network structure, dynamics, and function using NetworkX. Los Alamos National Lab.(LANL), Los Alamos, NM (United States); 2008.

31. Barabási AL. Network science. Cambridge university press; 2016.

32. Batagelj V, Zaversnik M. An O(m) algorithm for cores decomposition of networks. arXiv preprint cs/0310049. 2003;.

33. Fortunato S, Hric D. Community detection in networks: A user guide. Physics reports. 2016;659:1–44.

34. Blondel VD, Guillaume JL, Lambiotte R, Lefebvre E. Fast unfolding of communities in large networks. Journal of statistical mechanics: theory and experiment. 2008;2008(10):P10008.

35. Girvan M, Newman ME. Community structure in social and biological networks. Proceedings of the national academy of sciences. 2002;99(12):7821–7826.

36. Freeman LC. A set of measures of centrality based on betweenness. Sociometry. 1977; p. 35–41.

37. Page L, Brin S, Motwani R, Winograd T. The PageRank citation ranking: Bringing order to the web. Stanford InfoLab; 1999.

38. Mann HB. Nonparametric tests against trend. Econometrica: Journal of the Econometric Society. 1945; p. 245–259.

39. Kendall MG. Rank correlation methods. 1948;. Journal etc or publisher

40. Efron B, Hastie T. Computer age statistical inference. vol. 5. Cambridge University Press; 2016.

41. Ratkiewicz J, Conover MD, Meiss M, Gonçalves B, Flammini A, Menczer FM. Detecting and tracking political abuse in social media. In: Fifth international AAAI conference on weblogs and social media; 2011.

42. McCombs ME, Shaw DL, Weaver DH. New directions in agenda-setting theory and research. Mass communication and society. 2014;17(6):781–802.

43. Vargo CJ, Guo L, Amazeen MA. The agenda-setting power of fake news: A big data analysis of the online media landscape from 2014 to 2016. New Media & Society. 2018;20(5):2028–2049. doi:10.1177/1461444817712086.

44. Horne BD, Adali S. This just in: fake news packs a lot in title, uses simpler, repetitive content in text body, more similar to satire than real news. arXiv preprint arXiv:170309398. 2017;.

45. Zajonc RB. Mere exposure: A gateway to the subliminal. Current directions in psychological science. 2001;10(6):224–228.

46. Blei DM, Ng AY, Jordan MI. Latent dirichlet allocation. Journal of machine Learning research. 2003;3(Jan):993–1022.

47. Wang P, Angarita R, Renna I. Is this the era of misinformation yet: combining social bots and fake news to deceive the masses. In: Companion Proceedings of the The Web Conference 2018. International World Wide Web Conferences Steering Committee; 2018. p. 1557–1561.

48. Amnesty I. Il Barometro dell'odio - Elezioni europee 2019; 2019. URL?

Treated like a person name.

49. Conti N. Elezioni europee, ma poca Europa. La Repubblica. 2019;.

50. FactCheckEU. Good news and bad news after election week-end. 2019;.

51. McCombs M. Setting the agenda: Mass media and public opinion. John Wiley & Sons; 2018.

52. Davis CA, Varol O, Ferrara E, Flammini A, Menczer F. Botornot: A system to evaluate social bots. In: Proceedings of the 25th International Conference Companion on World Wide Web. International World Wide Web Conferences Steering Committee; 2016. p. 273–274.

Manuscript PONE-D-19-20362
Investigating Italian disinformation spreading on Twitter in the context of 2019 European elections

Response to Reviewers

We appreciate the interest that the Editor and the Reviewers have shown in our manuscript and the constructive criticism they have given. We addressed the concerns of all Reviewers, which you will find below in a point-by-point response. Correspondingly, we revised several parts of the manuscript (the new or modified text is in blue in the revised manuscript).

In summary:

- We clarified the main issues raised by reviewers, namely **(1)** the discrepancy between our results and the data we made publicly available, **(2)** the definition of re-tweeting network and **(3)** the topic analysis. The first was basically due to the upload of the wrong file which would not allow to reproduce our results. The second was a misuse of the term "re-tweeting", and we accordingly renamed the section "Building Twitter diffusion network": we actually considered Twitter social interactions altogether when building the network, i.e. tweets, retweets, quotes, replies and mentions. Finally, we reformulated the section on the topic analysis adding more details on the followed procedure and providing a table for keywords belonging to different topics.
- We addressed minor issues highlighted by reviewers and we overall improved the readability of the manuscript, correcting typos, grammar mistakes and rephrasing sentences.

We are grateful to the Reviewers for their comments and suggestions, which contributed to improve clarity and completeness of our manuscript.

REVIEWER 1

The manuscript offers an interesting and timely perspective on the public debate in the Italian Twittersphere during the last European Parliamentary Elections.
The few available academic contributions so far seem to agree on the limited scope of online disinformation during the campaign; social bots and botnets also seem to have played a limited role. In this respect, the work under examination appears to be in line with other studies on the same topic.
The authors adopt a balanced and informed stance. The manuscript does not offer - nor promise, indeed - an innovative approach to the study of online disinformation, yet it can usefully contribute to the interdisciplinary analysis of political participation during a main event such as the EP elections.
The technical analyses have been carried out appropriately and clearly described.
The manuscript is well organized and written clearly enough to be accessible to non specialists.

I have three comments on:
1) data collection
2) bot detection
3) style - some suggestions to improve the readability of the manuscript (typos, false friends, possible rephrasing).

1) The authors state (line 109) that they gathered "100% of shared tweets matching the defined query (see next).". I wonder whether they applied for and got access to the new Premium APIs, or adopted the standard Streaming API - as declared above (line 106). I think this point might need some clarification.

We actually used the standard (free) Streaming API. We added a comment in that sense to clarify it. The 100% argument was taken from ref'8 (Shao et al. 2018) I find nothing on this in Shao et al.

?

2) The authors are well aware that social bots can have a direct role in spreading disinformation, therefore adopting state-of-the-art bot detection techniques might be beneficial for a thorough analysis of the pollution of the online debate. Recent and promising techniques are oriented towards group (rather than account-by-account) analysis and unsupervised (rather than supervised) approaches; among them: Mazza M. et al. (2019), RTBust, Cresci S. et al. (2017), Social Fingerprinting. I am not implying the authors shoud adopt one of them, but they might consider justifying the use of the Botometer algorithm compared to other

approaches.

We thank the reviewer for the references; we practically used Botometer to compare results with findings in ref'8 (although we used only language independent features whereas Shao et al. employed them all). We added a paragraph where we describe such comparison, i.e. bots have a limited impact in the Italian misinformation network.

3) I will list my suggestions: …

We followed the suggestions and revised the text accordingly.

REVIEWER 2

The paper discusses some results related to the spreading of false news in the period preceding the European elections. Overall, the paper is interesting, clear and relatively sound in the methods. I recommend the paper for publication after some minor issues will be fixed.

I think the authors should introduce a bit better the context of European elections as well as the major concerns in the public debate regarding such an event. This would provide a better contextualization to the paper.

We added several paragraphs in that sense in the Introduction section.

Line 28: "As more people is more and more suspicious towards traditional media coverage". This statement should be supported by some empirical evidence (facts) or by some citations.

We added the corresponding citation.

Line 48: "trust in news is today particularly low (40% in general, 23% on social media)". What these percentages refer to?

According to Reuters 2019 Digital News report, these percentages were obtained with a questionnaire (available at http://media.digitalnewsreport.org/wp-content/uploads/2018/06/2018-Digital-News-Report-Survey-FINAL.pdf?x89475). We clarified in the text that 40% refers to the fraction of people that trust overall news most of the time, whereas only 23% trust news in social media most of the time.

Line 134: "We can observe that, in general, articles devoted to debunk false claims were barely engaged, accounting only for 6% of the total volume of tweets spreading disinformation 135 in the same period;". This is expected also considering the very low effectiveness of debunking strategies (see Zollo, F., Bessi, A., Del Vicario, M., Scala, A., Caldarelli, G., Shekhtman, L., ... & Quattrociocchi, W. (2017). Debunk- ing in a world of tribes. PloS one, 12(7), e0181821).

We added a comment in that sense (and the reference was already included in our bibliography).

Legend of Figure 2 is too small.

We modified the figure with a bigger legend.

Line 155: Authors use as subsection name "Building the re-tweeting network". How- ever, as far as I understood, their network doesn't comprehend re-tweets only. About this aspect: the authors aggregate different kind of interactions that sometimes are treated separately. Indeed the re-tweets can be associated to an endorsement pro- cedure while mentions etc.. can be associated to a communication procedure. The fact of aggregating is not necessarily wrong, however the authors should discuss the different possibilities of treating their data.

There was actually a misuse of the term “re-tweeting”: we are in fact considering Twitter social interactions altogether and we renamed the section as “Building Twitter diffusion network”. Treating separately different actions is an interesting point but it is out the of scope of our analysis.

Line 222-226: this paragraph results hard to follow. I suggest to the authors to spend few more lines in explaining the ratio behind the FWER.

We revised the sub-section and improved its readability.

Figure 3: In the caption the author say ”total number of shared articles” but the axis annotation says ”published”. I see that the two words can be used interchangeably but in the context of social media where ”sharing” and ”publishing” refer to a precise actions I would suggest to not interchange the two words.

We modified the figure accordingly.

Line 285: The authors divide users in different classes based on their activity. How the partitioning that they implement resembles the distribution of users’ activity? On what principles is their partitioning based?

Our partitioning is arbitrary and by no means definitive: our aim was to basically separate different users activity between two extremes (only 1 article and more than 1,000), which denote respectively a negligible involvement and a massive participation in the diffusion of malicious items.

Lines 301-315: The authors discuss a series of findings that are not supported by Figures or other material.

We added a figure (now Fig 5A) in that sense.

Figure 5: Pie charts are well known for being bad tools for summarizing data. Maybe a stacked bar chart would be better.

We modified the plot (now Fig 5B) with a stacked bar-chart.

Line 342: Are the titles somehow pre-processed? All the words mentioned by the authors are nouns so I assume that POS tagging has been performed. Additionally, the list of keywords provided by the volunteer should be better specified. How many words did they provide per topic? Afterwards they say that one of the most frequent keyword is ”video”. So I assume that volunteers provided the list of keywords while looking at the data rather than just providing words ”of interest” during the period of european elections. Also the topics that ”emerge” from the data are sometimes referred as categories. Summarizing I think that this part of the paper should be written in a more clear and precise way.

We actually considered all words, regardless of their Part-of-Speech tagging. We reformulated the section clarifying the “data-driven” flavor of our dictionary-based approach, and providing a table (now Table 1) with Top-20 keywords in each topic. We also dropped the word “categories” which could raise misunderstandings.

Line 356: ”proving that a remarkable fraction of disinformation was shared as mul- timedia content”. Reference/s needed.

We added a reference and we changed “proving” with “suggesting”, which seems more appropriate in this context.

Line 427: ”Despite centrality measures do not generally agree in their ranking”. However, centrality measures (degree, eigenvector, closeness) normally display strong correlations among each other so they may display small differences in the ranking but overall display a good agreement. (see for instance, Valente, T. W., Coronges, K., Lakon, C., & Costenbader, E. (2008). How correlated are network centrality measures?. Connections (Toronto, Ont.), 28(1), 16., among others)

We thank the reviewer for the reference. We originally reported that sentence from ref'4 *("Given that these metrics capture fundamentally different notions of centrality, we expect them to produce different rankings of the nodes in the network")*; we rephrased the sentence according to the reviewer comment.

• Line 495: "measures" is used instead of "measure"

We corrected it.

REVIEWER 3

This paper gives an overview of the presence of misinformation in the Italian 2019 European Parliament elections. The paper looks at both Twitter, and a set of misinformation websites that are mentioned on Twitter, and their hyperlink network.

To give a view of what the paper actually does, I listed the headers, their focus, and their results:

1. Assessing the reach
1.1 Sources of disinformation: the outlets behind most tweeted items; a few outlets represented most of the deceptive information.
1.2 User activity: distribution of sharing: a few users were responsible for most of the sharing.
2. The agenda-setting of disinformation
2.1 Topic analysis: comparing prevalence between a number of topics: European election receives limited focus compared to local political debate issues.
2.2 Use of hashtags: common hashtags: mostly support certain parties and debate topics (not mentioned in conclusions)
3. Relevant spreaders of disinformation
3.1 Central users in the main core: top central users of spreading, top targets, community identification, prevalence of bots: identifies top users according to different measures, top targets according to unspecified measures, manually classified communities, and that there are 2 bots.
3.2 Dismantling the disinformation network: removing users with top-in-strength is the most efficient way to break up the network.
4. Coordinated strategies of deception: looking at hyperlink network: the websites often link each other and sometimes feature translated articles.

As can be seen here, the paper effectively gives an overview by answering a number of questions about misinformation in the Italian context. I in particular appreciate the way the paper mixes computational methods with qualitative approaches involving actually looking at the data. While there are a number of issues, the paper is useful enough to warrant publication, given that a number of problems are addressed.

First an issue of concern, which I'm sure that there's a good explanation for. The articles specifies that 16,867 articles were collected (L131). Looking at the articles in the uploaded data material however, there are 27,436 articles. Over 21% of these are shorter than 50 letters, and their content tends to be things like "0", 'no_content', 'Scarica (PDF, 1.58MB)' – implying that the scraper failed in their collection. So, first, why the discrepancy in numbers between the pkl and the paper? Second, this makes me concerned that some articles were left out due to scraper error – which would imply a sampling problem.

We thank the reviewer for pointing out the issue and we apologize for the extra-effort required to assess the correctness of our results. We actually uploaded the wrong file, which contains also fact-checking articles and several duplicates (same title and body but different URL). Therefore we changed it with the correct file which allows to reproduce our results (and which contains exactly 16,867 articles). Ok!
Concerning possible scraping errors, we are aware that our crawler (which is actually composed of 20+ scripts due to the very different layouts of disinformation websites) might have failed to scrape the content of some articles—we count 5% of articles with empty body, corresponding to 11k shared tweets, but this could be due also to the fact that "video-only" articles have no content—as the same websites could exhibit different HTML structure for different articles. However, the issue with the content does not affect neither the descriptive statistics nor the topic analysis (as we focused on titles, which are missing/incorrect in only a dozen of items) → Wouldn't it also affect the hyperlink network? In any case this might be worth mentioning as a limitation?

Since the paper is basically doing a broad explorative overview over some arbitrarily selected – but interesting – questions, it becomes a challenge for the authors to make it seem coherent. Now, I don't mind the questions being arbitrary (others, however, may certainly mind) – but the paper does need to feel like a more or less coherent assemblage, rather than a motley pile of investigations. At times, the authors are not quite able to walk this tightrope, and the paper ends up feeling "too much", or just poorly structured. I would advise the authors to give the paper another run-through, attempting to tighten it, and work with well-polished signposting to tell a clearer and more coherent story. I would also consider removing some of the analyses, if they cannot be made to fit an overall narrative, or aren't used in conclusions or abstract (for instance the hashtag analysis, bot analysis and dismantling come to mind.) Try to focus it a bit more.

→ *Relatedly, I would also recommend having the text checked by a proofreader before publication, as there are some language issues.* This applies extra to the new text.

Another major issue is that there are multiple problems with how the results of the empirical work are interpreted by the authors. Worryingly, there are a number of statements that to me seem stronger than the actual results would permit. This leaves the feeling of "hyping" the results.

For instance, already in the abstract:
--"the deceptive information … was driven by controversial and polarizing topics of debate such as immigration, national safety and (Italian) nationalism" (c.f. RQ4) Since the "topics" are hand-coded, I don't see how this could be concluded from the method: you're able to distinguish how these selected topics compare to each other, given some assumptions, but not how well they cover the material. (OTHER captures articles that contain no keywords – this is not a satisfying method for making this statement.) See also comment below.
--"We unraveled the existence of an intricate network of connections between different disinformation outlets across Europe, U.S. and Russia, which seemingly acted in a coordinated manner in the period before the elections." This, in my understanding, points to the study of the hyperlink network of the misinformation sites and that some sites feature translations of other sites' articles. To describe this as to "unravel" an "intricate network" acting in a "coordinated manner" is, to say the least, to oversell the analysis. Where is the evidence of coordination? That I'm hyperlinking, or even translating, from another website does not imply that we are coordinated. More accurately would be to say that "different misinformation sites across Europe link to each other and feature similar, even translated, articles".

Overall, the paper therefore paints an image of sinister Russian interests meddling in the elections – which I find inadequate backing for in the analyses. (A more honest abstract would probably mention that there is limited interest for the European Election in both the news and in the disinformation outlets...)

We thank the reviewer for the insightful comments, and we revised our manuscript accordingly as not to deceive the reader with an "over-sold" analysis. The reviewer will appreciate that all the above comments have been taken into account in the revision of the Abstract, Introduction and Conclusions sections (as well as minor fixes throughout the paper). Good!

RQ2: The question conflates "narrative" and "topic". These are two distinct things, with particular meanings in the literature. How do you conceptualize your method?

We dropped the term "narrative" as it would imply a deeper analysis of the style and communication strategies employed by disinformation media, whereas we only focus on the content and the topics of deceptive outlets. ok!

Topic analysis: for this analysis, you focus on the titles of the articles that were shared at least once. You then conclude that e.g. "automatic" topic modeling (I assume you are referring to unsupervised methods such as LDA) work poorly on short texts, and therefore you go ahead and select a method that doesn't seem able to answer your research question? This is rather strange to me: you do have the full article, so why don't you just go ahead and use it for "automatic" topic modeling – it should definitely be long enough? In any case, as I've already noted, if your question is the prevalence of these topics, the method is less than great. Even using the hashtags for this analysis would be more convincing.

We re-formulated the section and clarified some aspects of our procedure, including the hand-coding of topics and the keywords employed. We focused on titles because we aim to take into account the perceived

prevalence on Twitter of disinformation topics, as titles might impact on people who do not visit explicitly these links but encounter them on their social feed (we added a reference in the initial paragraph of the "Topic Analysis" section, i.e. *Robert B Zajonc. Mere exposure: A gateway to the subliminal. Current directions in psychological science, 10(6):224–228, 2001)*. Besides, we avoided LDA-like techniques because they did not entail satisfactory results.

fair enough. would like more detailed description of method though, or a reference.

The "retweeting diffusion network" is in fact not a retweet network, but also includes e.g. replies and mentions. There seem to me to have quite different meanings: replies and mentions are quite likely to be critical, etc. (There's plenty of literature on this.) Mixing them might be fine, but it should be motivated by an answer to the question: what is the network actually intended to represent?
The wheels hit the road on this question when looking for instance at Table 1, in which centrality is evaluated through various measures. That matteosalvinimi is identified as a top disinformation user, despite having a single misinformation tweet, begs the question of what is actually measured.

We acknowledge a misuse of the word "re-tweeting" and we renamed the section in "Building Twitter diffusion networks" as we actually intend to represent the entire network of social interactions, thus considering Twitter actions altogether (tweets, retweets, replies, quotes, mentions). We thank the reviewer for highlighting a deeper analysis on different actions and we leave it for future research. OK

Some of the analyses aren't described in sufficient detail. For instance, (pp17) how are "targets" of misinformation identified? Do go through and double-check methodological specificity.

We manually recognized, in the rankings of centrality measures, public figures which have been repeatedly reported as targets of misinformation campaigns on Italian newspapers.

This sounds so unsystematic that I would prefer just leaving out this part...

"The agenda-setting of misinformation". I see no evidence for the "setting" in the "agenda-setting" here, these are just themes, and it anyway seems to be suggested that they follow mainstream news. Perhaps just "content of misinformation" would suffice?

We added a comment to clarify the meaning of agenda-setting effects at the beginning of "Topic analysis" subsection. As a matter of fact we referred to a passage from "***The agenda-setting power of fake news: A big data analysis of the online media landscape from 2014 to 2016***" *(Varguo et al. 2018) which we report here:*

"Agenda-setting theory originally examined what topics trend in the news and how that affects the opinions of audiences (McCombs, 2014). The first level of agenda setting asserts that the frequency in which news media mention and cover objects (e.g. issues and public figures) largely dictates what objects audiences think are important to society. This is not to say that audiences blindly believe the news. Instead, the news media sets the public salience for objects or attributes. When substantial news coverage is dedicated to an issue (e.g. economy), people consider the economy an important issue—even though audiences may have diverging opinions about the issue (e.g. how to fix the economy). This nuance is critical when considering the agenda-setting power of fake news: even if some audience members are aware that fake news is fake, the mere rise in coverage (fake or real) could result in an agenda-setting effect."

OK, fair!

The edge weight of the hyperlink network is a function of the number of tweets linking to the source. I'd like some type of motivation for this, as it doesn't make obvious intuitive sense to me.

We aimed to measure the extent of different inter-connections in terms of their social audience, thus projecting the corresponding number of shares in the articles containing hyper-links. Therefore, we arbitrarily give more importance to articles that were repeatedly shared on Twitter.

Well, OK...

Comments on figures:

- *Figure 1. "Network failure": does this mean that the tweet collection went down? Perhaps better to just leave out the data points as it just makes the graph hard to read? That the number of tweets peaks before election should perhaps be emphasized a bit more: while the election isn't a thematic focus of misinformation, this implies that it does seem to be a strategic focus.*

The failure refers to our data collection, which we prefer to keep in the figure as it denotes some periods where we couldn't cover disinformation spreading. For what concerns the increasing trend before the election, this is detected only in "byoblu.com" (and we already remarked it) and we actually observe that the

higher volume is only due to the outlet “Ilprimatonazionale.it”, which we added at the end of April, as discussed in the sub-section “Sources of disinformation”.

- *Figure 6: Pie chart on distribution of sharing. I’m not completely content with this vis. Perhaps a standard log-log plot would be better?*

We changed the figure a stacked-bar chart.

- *Figure5B.tiff: I’m not able to match this to any figure in the text?*

The reference was actually in the text, and now you can find it at Line 353 (renamed as Fig5C).

- *Figure11A.tiff: perhaps add labels on the nodes?*

We noticed that the figure was hard to read, with too many (and very long) labels on nodes. Therefore, we prefer the current more succinct version with a simple legenda for different communities.

Other minor comments:

- *Please use commas for thousands separators throughout: “23243” is hard to read.*
- *Time series analysis is introduces on pp8 without any indications for what it will be used. Add a sentence.*
- *“proving that a remarkable fraction of disinformation” – this is too strong. It may “imply” or “suggest” it.*
- *Pp 14, L 389-390. “because European elections are extremely un-polarised and not very interesting compared to national elections.” I would avoid this formulation (also, “extremely un-polarised” is a terrible phrasing.)*
- *Pp15 L.417: “**Relevant** spreaders of disinformation”. Why relevant? Choice of words.*
- *Pp 17 L 452: “a major issue with disinformation”. Rather a “strategy” than an “issue”?*

We followed the suggestions and changed the text accordingly.

Data Review URL

Click here to download Data Review URL
http://doi.org/10.7910/DVN/OQHLAJ
