# Supplementary material for: Investigating Italian disinformation spreading on Twitter in the context of 2019 European elections
Source: PLoS One. 2020 Jan 17;15(1):e0227821. doi: 10.1371/journal.pone.0227821 (PMC6968875; doi:10.1371/journal.pone.0227821)
Supplement: Attachment — Submitted filename: paper.pdf [file pone.0227821.s005.pdf]

The authors improved the paper and (shortly) addressed my concerns. Reading again the paper I noted very few points that captured my attention and that need further clarification:

- One of the main findings of the article is the evidence of the interconnections among different outlets across the world with evident connections between Italy and Russia. Every analysis in the paper has been subject to statistical validation except this one. I think the author should perform a statistical validation of such evidence. For instance, they could run several instances of a rewiring algorithm (degree preserving or strength preserving or both) over the network of Fig 11B to test if the percentage of links across countries in higher/lower than expected.
- Figure 1: the authors should specify what they mean by network failure.
- Figure 5: what are the clearer bars on top of the darker bars? Perhaps confidence intervals? The authors should clarify this aspect.
- A paragraph is titled "Dismantling the disinformation network". In the paper there are two networks the Twitter diffusion network and the network of websites. The disinformation network (according to what I understood and guessing from the analysis they carried on) should be the Twitter diffusion network but I am not sure. The authors should be clear in this regard.

As a final remark I suggest the authors to be very specific and precise when referring to or when interlinking the different elements of the paper. Indeed, they carry on a lot of different analyses on different networks and the feeling of the reader is to be, from time to time, a bit lost.
