# Supplementary material for: Investigating Italian disinformation spreading on Twitter in the context of 2019 European elections
Source: PLoS One. 2020 Jan 17;15(1):e0227821. doi: 10.1371/journal.pone.0227821 (PMC6968875; doi:10.1371/journal.pone.0227821)
Supplement: Attachment — Submitted filename: review.docx [file pone.0227821.s002.docx]

This paper gives an overview of the presence of misinformation in the Italian 2019 European Parliament elections. The paper looks at both Twitter, and a set of misinformation websites that are mentioned on Twitter, and their hyperlink network.

To give a view of what the paper actually does, I listed the headers, their focus, and their results:

1. Assessing the reach

1.1 Sources of disinformation: *the outlets behind most tweeted items; a few outlets represented most of the deceptive information.*

1.2 User activity: *distribution of sharing: a few users were responsible for most of the sharing.*

2. The agenda-setting of disinformation

2.1 Topic analysis: *comparing prevalence between a number of topics: European election receives limited focus compared to local political debate issues.*

2.2 Use of hashtags: *common hashtags: mostly support certain parties and debate topics* (not mentioned in conclusions)

3. Relevant spreaders of disinformation

3.1 Central users in the main core: *top central users of spreading, top targets, community identification, prevalence of bots: identifies top users according to different measures, top targets according to unspecified measures, manually classified communities, and that there are 2 bots.*

3.2 Dismantling the disinformation network: *removing users with top-in-strength is the most efficient way to break up the network.*

4. Coordinated strategies of deception: *looking at hyperlink network: the websites often link each other and sometimes feature translated articles.*

As can be seen here, the paper effectively gives an overview by answering a number of questions about misinformation in the Italian context. I in particular appreciate the way the paper mixes computational methods with qualitative approaches involving actually looking at the data. While there are a number of issues, the paper is useful enough to warrant publication, given that a number of problems are addressed.

First an issue of concern, which I’m sure that there’s a good explanation for. The articles specifies that 16,867 articles were collected (L131). Looking at the articles in the uploaded data material however, there are 27,436 articles. Over 21% of these are shorter than 50 letters, and their content tends to be things like “0”, ‘no_content’, ‘Scarica (PDF, 1.58MB)’ – implying that the scraper failed in their collection. So, first, why the discrepancy in numbers between the pkl and the paper? Second, this makes me concerned that some articles were left out due to scraper error – which would imply a sampling problem.

Since the paper is basically doing a broad explorative overview over some arbitrarily selected – but interesting – questions, it becomes a challenge for the authors to make it seem coherent. Now, I don’t mind the questions being arbitrary (others, however, may certainly mind) – but the paper does need to feel like a more or less coherent assemblage, rather than a motley pile of investigations. At times, the authors are not quite able to walk this tightrope, and the paper ends up feeling “too much”, or just poorly structured. I would advise the authors to give the paper another run-through, attempting to tighten it, and work with well-polished signposting to tell a clearer and more coherent story. I would also consider removing some of the analyses, if they cannot be made to fit an overall narrative, or aren’t used in conclusions or abstract (for instance the hashtag analysis, bot analysis and dismantling come to mind.) Try to focus it a bit more.

Relatedly, I would also recommend having the text checked by a proofreader before publication, as there are some language issues.

Another major issue is that there are multiple problems with how the results of the empirical work are interpreted by the authors. Worryingly, there are a number of statements that to me seem stronger than the actual results would permit. This leaves the feeling of “hyping” the results.

For instance, already in the abstract:

--“the deceptive information … was driven by controversial and polarizing topics of debate such as immigration, national safety and (Italian) nationalism” (c.f. RQ4) Since the “topics” are hand-coded, I don’t see how this could be concluded from the method: you’re able to distinguish how these selected topics compare to each other, given some assumptions, but not how well they cover the material. (OTHER captures articles that contain no keywords – this is not a satisfying method for making this statement.) See also comment below.

--“We unraveled the existence of an intricate network of connections between different disinformation outlets across Europe, U.S. and Russia, which seemingly acted in a coordinated manner in the

period before the elections.” This, in my understanding, points to the study of the hyperlink network of the misinformation sites and that some sites feature translations of other sites’ articles. To describe this as to “unravel” an “intricate network” acting in a “coordinated manner” is, to say the least, to oversell the analysis. Where is the evidence of coordination? That I’m hyperlinking, or even translating, from another website does not imply that we are coordinated. More accurately would be to say that “different misinformation sites across Europe link to each other and feature similar, even translated, articles”.

Overall, the paper therefore paints an image of sinister Russian interests meddling in the elections – which I find inadequate backing for in the analyses. (A more honest abstract would probably mention that there is limited interest for the European Election in both the news and in the disinformation outlets...)

RQ2: The question conflates “narrative” and “topic”. These are two distinct things, with particular meanings in the literature. How do you conceptualize your method?

Topic analysis: for this analysis, you focus on the titles of the articles that were shared at least once. You then conclude that e.g. “automatic” topic modeling (I assume you are referring to unsupervised methods such as LDA) work poorly on short texts, and therefore you go ahead and select a method that doesn’t seem able to answer your research question? This is rather strange to me: you do have the full article, so why don’t you just go ahead and use it for “automatic” topic modeling – it should definitely be long enough? In any case, as I’ve already noted, if your question is the prevalence of these topics, the method is less than great. Even using the hashtags for this analysis would be more convincing.

The “retweeting diffusion network” is in fact not a retweet network, but also includes e.g. replies and mentions. There seem to me to have quite different meanings: replies and mentions are quite likely to be critical, etc. (There’s plenty of literature on this.) Mixing them might be fine, but it should be motivated by an answer to the question: what is the network actually intended to represent?

The wheels hit the road on this question when looking for instance at Table 1, in which centrality is evaluated through various measures. That matteosalvinimi is identified as a top disinformation user, despite having a single misinformation tweet, begs the question of what is actually measured.

Some of the analyses aren’t described in sufficient detail. For instance, (pp17) how are “targets” of misinformation identified? Do go through and double-check methodological specificity.

“The agenda-setting of misinformation”. I see no evidence for the “setting” in the “agenda-setting” here, these are just themes, and it anyway seems to be suggested that they follow mainstream news. Perhaps just “content of misinformation” would suffice?

The edge weight of the hyperlink network is a function of the number of tweets linking to the source. I’d like some type of motivation for this, as it doesn’t make obvious intuitive sense to me.

Comments on figures:

- Figure 1. “Network failure”: does this mean that the tweet collection went down? Perhaps better to just leave out the data points as it just makes the graph hard to read? That the number of tweets peaks before election should perhaps be emphasized a bit more: while the election isn’t a thematic focus of misinformation, this implies that it does seem to be a strategic focus.
- Figure 6: Pie chart on distribution of sharing. I’m not completely content with this vis. Perhaps a standard log-log plot would be better?
- Figure5B.tiff: I’m not able to match this to any figure in the text?
- Figure11A.tiff: perhaps add labels on the nodes?

Other minor comments:

- Please use commas for thousands separators throughout: “23243” is hard to read.
- Time series analysis is introduces on pp8 without any indications for what it will be used. Add a sentence.
- “proving that a remarkable fraction of disinformation” – this is too strong. It may “imply” or “suggest” it.
- Pp 14, L 389-390. “because European elections are extremely un-polarised and not very interesting compared to national elections.” I would avoid this formulation (also, “extremely un-polarised” is a terrible phrasing.)
- Pp15 L.417: “**Relevant** spreaders of disinformation”. Why relevant? Choice of words.
- Pp 17 L 452: “a major issue with disinformation”. Rather a “strategy” than an “issue”?
