# Supplementary material for: Investigating Italian disinformation spreading on Twitter in the context of 2019 European elections
Source: PLoS One. 2020 Jan 17;15(1):e0227821. doi: 10.1371/journal.pone.0227821 (PMC6968875; doi:10.1371/journal.pone.0227821)
Supplement: Attachment — Submitted filename: response_reviewers_final.docx [file pone.0227821.s003.docx]

**Manuscript PONE-D-19-20362**

**Investigating Italian disinformation spreading on Twitter in the context of 2019 European elections**

# Response to Reviewers

## We appreciate the interest that the Editor and the Reviewers have shown in our manuscript and the constructive criticism they have given. We addressed the concerns of all Reviewers, which you will find below in a point-by-point response. Correspondingly, we revised several parts of the manuscript (the new or modified text is in blue in the revised manuscript).

## In summary:

## We clarified the main issues raised by reviewers, namely **(1)** the discrepancy between our results and the data we made publicly available, **(2)** the definition of re-tweeting network and **(3)** the topic analysis. The first was basically due to the upload of the wrong file which would not allow to reproduce our results. The second was a misuse of the term “re-tweeting”, and we accordingly renamed the section “Building Twitter diffusion network”: we actually considered Twitter social interactions altogether when building the network, i.e. tweets, retweets, quotes, replies and mentions. Finally, we reformulated the section on the topic analysis adding more details on the followed procedure and providing a table for keywords belonging to different topics.

## We addressed minor issues highlighted by reviewers and we overall improved the readability of the manuscript, correcting typos, grammar mistakes and rephrasing sentences.

## We are grateful to the Reviewers for their comments and suggestions, which contributed to improve clarity and completeness of our manuscript.

**REVIEWER 1**

*The manuscript offers an interesting and timely perspective on the public debate in the Italian Twittersphere during the last European Parliamentary Elections.
The few available academic contributions so far seem to agree on the limited scope of online disinformation during the campaign; social bots and botnets also seem to have played a limited role. In this respect, the work under examination appears to be in line with other studies on the same topic.
The authors adopt a balanced and informed stance. The manuscript does not offer - nor promise, indeed - an innovative approach to the study of online disinformation, yet it can usefully contribute to the interdisciplinary analysis of political participation during a main event such as the EP elections.
The technical analyses have been carried out appropriately and clearly described.
The manuscript is well organized and written clearly enough to be accessible to non specialists.

I have three comments on:
1) data collection
2) bot detection
3) style - some suggestions to improve the readability of the manuscript (typos, false friends, possible rephrasing).*

*1) The authors state (line 109) that they gathered "100% of shared tweets matching the defined query (see next).". I wonder whether they applied for and got access to the new Premium APIs, or adopted the standard Streaming API - as declared above (line 106). I think this point might need some clarification.*

We actually used the standard (free) Streaming API. We added a comment in that sense to clarify it. The 100% argument was taken from ref’8 (Shao et al. 2018)

*2) The authors are well aware that social bots can have a direct role in spreading disinformation, therefore adopting state-of-the-art bot detection techniques might be beneficial for a thorough analysis of the pollution of the online debate. Recent and promising techniques are oriented towards group (rather than account-by-account) analysis and unsupervised (rather than supervised) approaches; among them: Mazza M. et al. (2019), RTBust, Cresci S. et al. (2017), Social Fingerprinting. I am not implying the authors shoud adopt one of them, but they might consider justifying the use of the Botometer algorithm compared to other approaches.*

We thank the reviewer for the references; we practically used Botometer to compare results with findings in ref’8 (although we used only language independent features whereas Shao et al. employed them all). We added a paragraph where we describe such comparison, i.e. bots have a limited impact in the Italian misinformation network.

*3) I will list my suggestions: …*

We followed the suggestions and revised the text accordingly.

**REVIEWER 2**

*The paper discusses some results related to the spreading of false news in the period preceding the European elections. Overall, the paper is interesting, clear and relatively sound in the methods. I recommend the paper for publication after some minor issues will be fixed.*

*I think the authors should introduce a bit better the context of European elections as well as the major concerns in the public debate regarding such an event. This would provide a better contextualization to the paper.*

We added several paragraphs in that sense in the Introduction section.

*Line 28: ”As more people is more and more suspicious towards traditional media coverage”. This statement should be supported by some empirical evidence (facts) or by some citations.*

We added the corresponding citation.

*Line 48: ”trust in news is today particularly low (40% in general, 23% on social media)”. What these percentages refer to?*

According to Reuters 2019 Digital News report, these percentages were obtained with a questionnaire (available at <http://media.digitalnewsreport.org/wp-content/uploads/2018/06/2018-Digital-News-Report-Survey-FINAL.pdf?x89475>). We clarified in the text that 40% refers to the fraction of people that trust overall news most of the time, whereas only 23% trust news in social media most of the time.

*Line 134: ”We can observe that, in general, articles devoted to debunk false claims were barely engaged, accounting only for 6% of the total volume of tweets spreading disinformation 135 in the same period;”. This is expected also considering the very low effectiveness of debunking strategies (see Zollo, F., Bessi, A., Del Vicario, M., Scala, A., Caldarelli, G., Shekhtman, L., ... & Quattrociocchi, W. (2017). Debunk- ing in a world of tribes. PloS one, 12(7), e0181821).*

We added a comment in that sense (and the reference was already included in our bibliography).

*Legend of Figure 2 is too small.*

We modified the figure with a bigger legend.

*Line 155: Authors use as subsection name ”Building the re-tweeting network”. How- ever, as far as I understood, their network doesn’t comprehend re-tweets only. About this aspect: the authors aggregate different kind of interactions that sometimes are treated separately. Indeed the re-tweets can be associated to an endorsement pro- cedure while mentions etc.. can be associated to a communication procedure. The fact of aggregating is not necessarily wrong, however the authors should discuss the different possibilities of treating their data.*

There was actually a misuse of the term “re-tweeting”: we are in fact considering Twitter social interactions altogether and we renamed the section as “Building Twitter diffusion network”. Treating separately different actions is an interesting point but it is out the of scope of our analysis.

*Line 222-226: this paragraph results hard to follow. I suggest to the authors to spend few more lines in explaining the ratio behind the FWER.*

We revised the sub-section and improved its readability.

*Figure 3: In the caption the author say ”total number of shared articles” but the axis annotation says ”published”. I see that the two words can be used interchangeably but in the context of social media where ”sharing” and ”publishing” refer to a precise actions I would suggest to not interchange the two words.*

We modified the figure accordingly.

*Line 285: The authors divide users in different classes based on their activity. How the partitioning that they implement resembles the distribution of users’ activity? On what principles is their partitioning based?*

Our partitioning is arbitrary and by no means definitive: our aim was to basically separate different users activity between two extremes (only 1 article and more than 1,000), which denote respectively a negligible involvement and a massive participation in the diffusion of malicious items.

*Lines 301-315: The authors discuss a series of findings that are not supported by Figures or other material.*

We added a figure (now Fig 5A) in that sense.

*Figure 5: Pie charts are well known for being bad tools for summarizing data. Maybe a stacked bar chart would be better.*

We modified the plot (now Fig 5B) with a stacked bar-chart.

*Line 342: Are the titles somehow pre-processed? All the words mentioned by the authors are nouns so I assume that POS tagging has been performed. Additionally, the list of keywords provided by the volunteer should be better specified. How many words did they provide per topic? Afterwards they say that one of the most frequent keyword is ”video”. So I assume that volunteers provided the list of keywords while looking at the data rather than just providing words ”of interest” during the period of european elections. Also the topics that ”emerge” from the data are sometimes referred as categories. Summarizing I think that this part of the paper should be written in a more clear and precise way.*

We actually considered all words, regardless of their Part-of-Speech tagging. We reformulated the section clarifying the “data-driven” flavor of our dictionary-based approach, and providing a table (now Table 1) with Top-20 keywords in each topic. We also dropped the word “categories” which could raise misunderstandings.

*Line 356: ”proving that a remarkable fraction of disinformation was shared as mul- timedia content”. Reference/s needed.*

We added a reference and we changed “proving” with “suggesting”, which seems more appropriate in this context.

*Line 427: ”Despite centrality measures do not generally agree in their ranking”. However, centrality measures (degree, eigenvector, closeness) normally display strong correlations among each other so they may display small differences in the ranking but overall display a good agreement. (see for instance, Valente, T. W., Coronges, K., Lakon, C., & Costenbader, E. (2008). How correlated are network centrality measures?. Connections (Toronto, Ont.), 28(1), 16., among others)*

We thank the reviewer for the reference. We originally reported that sentence from ref’4 *(“Given that these metrics capture fundamentally different notions of centrality, we expect them to produce different rankings of the nodes in the network”*); we rephrased the sentence according to the reviewer comment.

*• Line 495: ”measures” is used instead of ”measure”*

We corrected it.

**REVIEWER 3**

*This paper gives an overview of the presence of misinformation in the Italian 2019 European Parliament elections. The paper looks at both Twitter, and a set of misinformation websites that are mentioned on Twitter, and their hyperlink network.*

*To give a view of what the paper actually does, I listed the headers, their focus, and their results:*

*1. Assessing the reach*

*1.1 Sources of disinformation: the outlets behind most tweeted items; a few outlets represented most of the deceptive information.*

*1.2 User activity: distribution of sharing: a few users were responsible for most of the sharing.*

*2. The agenda-setting of disinformation*

*2.1 Topic analysis: comparing prevalence between a number of topics: European election receives limited focus compared to local political debate issues.*

*2.2 Use of hashtags: common hashtags: mostly support certain parties and debate topics (not mentioned in conclusions)*

*3. Relevant spreaders of disinformation*

*3.1 Central users in the main core: top central users of spreading, top targets, community identification, prevalence of bots: identifies top users according to different measures, top targets according to unspecified measures, manually classified communities, and that there are 2 bots.*

*3.2 Dismantling the disinformation network: removing users with top-in-strength is the most efficient way to break up the network.*

*4. Coordinated strategies of deception: looking at hyperlink network: the websites often link each other and sometimes feature translated articles.*

*As can be seen here, the paper effectively gives an overview by answering a number of questions about misinformation in the Italian context. I in particular appreciate the way the paper mixes computational methods with qualitative approaches involving actually looking at the data. While there are a number of issues, the paper is useful enough to warrant publication, given that a number of problems are addressed.*

*First an issue of concern, which I’m sure that there’s a good explanation for. The articles specifies that 16,867 articles were collected (L131). Looking at the articles in the uploaded data material however, there are 27,436 articles. Over 21% of these are shorter than 50 letters, and their content tends to be things like “0”, ‘no_content’, ‘Scarica (PDF, 1.58MB)’ – implying that the scraper failed in their collection. So, first, why the discrepancy in numbers between the pkl and the paper? Second, this makes me concerned that some articles were left out due to scraper error – which would imply a sampling problem.*

We thank the reviewer for pointing out the issue and we apologize for the extra-effort required to assess the correctness of our results. We actually uploaded the wrong file, which contains also fact-checking articles and several duplicates (same title and body but different URL). Therefore we changed it with the correct file which allows to reproduce our results (and which contains exactly 16,867 articles).
Concerning possible scraping errors, we are aware that our crawler (which is actually composed of 20+ scripts due to the very different layouts of disinformation websites) might have failed to scrape the content of some articles—we count 5% of articles with empty body, corresponding to 11k shared tweets, but this could be due also to the fact that “video-only” articles have no content—as the same websites could exhibit different HTML structure for different articles. However, the issue with the content does not affect neither the descriptive statistics nor the topic analysis (as we focused on titles, which are missing/incorrect in only a dozen of items)

*Since the paper is basically doing a broad explorative overview over some arbitrarily selected – but interesting – questions, it becomes a challenge for the authors to make it seem coherent. Now, I don’t mind the questions being arbitrary (others, however, may certainly mind) – but the paper does need to feel like a more or less coherent assemblage, rather than a motley pile of investigations. At times, the authors are not quite able to walk this tightrope, and the paper ends up feeling “too much”, or just poorly structured. I would advise the authors to give the paper another run-through, attempting to tighten it, and work with well-polished signposting to tell a clearer and more coherent story. I would also consider removing some of the analyses, if they cannot be made to fit an overall narrative, or aren’t used in conclusions or abstract (for instance the hashtag analysis, bot analysis and dismantling come to mind.) Try to focus it a bit more.*

*Relatedly, I would also recommend having the text checked by a proofreader before publication, as there are some language issues.*

*Another major issue is that there are multiple problems with how the results of the empirical work are interpreted by the authors. Worryingly, there are a number of statements that to me seem stronger than the actual results would permit. This leaves the feeling of “hyping” the results.*

*For instance, already in the abstract:*

*--“the deceptive information … was driven by controversial and polarizing topics of debate such as immigration, national safety and (Italian) nationalism” (c.f. RQ4) Since the “topics” are hand-coded, I don’t see how this could be concluded from the method: you’re able to distinguish how these selected topics compare to each other, given some assumptions, but not how well they cover the material. (OTHER captures articles that contain no keywords – this is not a satisfying method for making this statement.) See also comment below.*

*--“We unraveled the existence of an intricate network of connections between different disinformation outlets across Europe, U.S. and Russia, which seemingly acted in a coordinated manner in the*

*period before the elections.” This, in my understanding, points to the study of the hyperlink network of the misinformation sites and that some sites feature translations of other sites’ articles. To describe this as to “unravel” an “intricate network” acting in a “coordinated manner” is, to say the least, to oversell the analysis. Where is the evidence of coordination? That I’m hyperlinking, or even translating, from another website does not imply that we are coordinated. More accurately would be to say that “different misinformation sites across Europe link to each other and feature similar, even translated, articles”.*

*Overall, the paper therefore paints an image of sinister Russian interests meddling in the elections – which I find inadequate backing for in the analyses. (A more honest abstract would probably mention that there is limited interest for the European Election in both the news and in the disinformation outlets...)*

We thank the reviewer for the insightful comments, and we revised our manuscript accordingly as not to deceive the reader with an “over-sold” analysis. The reviewer will appreciate that all the above comments have been taken into account in the revision of the Abstract, Introduction and Conclusions sections (as well as minor fixes throughout the paper).

*RQ2: The question conflates “narrative” and “topic”. These are two distinct things, with particular meanings in the literature. How do you conceptualize your method?*

We dropped the term “narrative” as it would imply a deeper analysis of the style and communication strategies employed by disinformation media, whereas we only focus on the content and the topics of deceptive outlets.

*Topic analysis: for this analysis, you focus on the titles of the articles that were shared at least once. You then conclude that e.g. “automatic” topic modeling (I assume you are referring to unsupervised methods such as LDA) work poorly on short texts, and therefore you go ahead and select a method that doesn’t seem able to answer your research question? This is rather strange to me: you do have the full article, so why don’t you just go ahead and use it for “automatic” topic modeling – it should definitely be long enough? In any case, as I’ve already noted, if your question is the prevalence of these topics, the method is less than great. Even using the hashtags for this analysis would be more convincing.*

We re-formulated the section and clarified some aspects of our procedure, including the hand-coding of topics and the keywords employed. We focused on titles because we aim to take into account the perceived prevalence on Twitter of disinformation topics, as titles might impact on people who do not visit explicitly these links but encounter them on their social feed (we added a reference in the initial paragraph of the “Topic Analysis” section, i.e. *Robert B Zajonc. Mere exposure: A gateway to the subliminal. Current directions in psychological science, 10(6):224–228, 2001)*. Besides, we avoided LDA-like techniques because they did not entail satisfactory results.

*The “retweeting diffusion network” is in fact not a retweet network, but also includes e.g. replies and mentions. There seem to me to have quite different meanings: replies and mentions are quite likely to be critical, etc. (There’s plenty of literature on this.) Mixing them might be fine, but it should be motivated by an answer to the question: what is the network actually intended to represent?*

*The wheels hit the road on this question when looking for instance at Table 1, in which centrality is evaluated through various measures. That matteosalvinimi is identified as a top disinformation user, despite having a single misinformation tweet, begs the question of what is actually measured.*

We acknowledge a misuse of the word “re-tweeting” and we renamed the section in “Building Twitter diffusion networks” as we actually intend to represent the entire network of social interactions, thus considering Twitter actions altogether (tweets, retweets, replies, quotes, mentions). We thank the reviewer for highlighting a deeper analysis on different actions and we leave it for future research.

*Some of the analyses aren’t described in sufficient detail. For instance, (pp17) how are “targets” of misinformation identified? Do go through and double-check methodological specificity.*

We manually recognized, in the rankings of centrality measures, public figures which have been repeatedly reported as targets of misinformation campaigns on Italian newspapers.

*“The agenda-setting of misinformation”. I see no evidence for the “setting” in the “agenda-setting” here, these are just themes, and it anyway seems to be suggested that they follow mainstream news. Perhaps just “content of misinformation” would suffice?*

We added a comment to clarify the meaning of agenda-setting effects at the beginning of “Topic analysis” subsection. As a matter of fact we referred to a passage from “***The agenda-setting power of fake news: A big data analysis of the online media landscape from 2014 to 2016****“ (Varguo et al. 2018) which we report here:*

*”Agenda-setting theory originally examined what topics trend in the news and how that affects the opinions of audiences (McCombs, 2014). The first level of agenda setting asserts that the frequency in which news media mention and cover objects (e.g. issues and public figures) largely dictates what objects audiences think are important to society. This is not to say that audiences blindly believe the news. Instead, the news media sets the public salience for objects or attributes. When substantial news coverage is dedicated to an issue (e.g. economy), people consider the economy an important issue—even though audiences may have diverging opinions about the issue (e.g. how to fix the economy). This nuance is critical when considering the agenda-setting power of fake news: even if some audience members are aware that fake news is fake, the mere rise in coverage (fake or real) could result in an agenda-setting effect.”*

*The edge weight of the hyperlink network is a function of the number of tweets linking to the source. I’d like some type of motivation for this, as it doesn’t make obvious intuitive sense to me.*

We aimed to measure the extent of different inter-connections in terms of their social audience, thus projecting the corresponding number of shares in the articles containing hyper-links. Therefore, we arbitrarily give more importance to articles that were repeatedly shared on Twitter.

*Comments on figures:*

- *Figure 1. “Network failure”: does this mean that the tweet collection went down? Perhaps better to just leave out the data points as it just makes the graph hard to read? That the number of tweets peaks before election should perhaps be emphasized a bit more: while the election isn’t a thematic focus of misinformation, this implies that it does seem to be a strategic focus.*

The failure refers to our data collection, which we prefer to keep in the figure as it denotes some periods where we couldn’t cover disinformation spreading. For what concerns the increasing trend before the election, this is detected only in “byoblu.com” (and we already remarked it) and we actually observe that the higher volume is only due to the outlet “Ilprimatonazionale.it”, which we added at the end of April, as discussed in the sub-section “Sources of disinformation”.

- *Figure 6: Pie chart on distribution of sharing. I’m not completely content with this vis. Perhaps a standard log-log plot would be better?*

We changed the figure a stacked-bar chart.

- *Figure5B.tiff: I’m not able to match this to any figure in the text?*

The reference was actually in the text, and now you can find it at Line 353 (renamed as Fig5C).

- *Figure11A.tiff: perhaps add labels on the nodes?*

We noticed that the figure was hard to read, with too many (and very long) labels on nodes. Therefore, we prefer the current more succinct version with a simple legenda for different communities.

*Other minor comments:*

- *Please use commas for thousands separators throughout: “23243” is hard to read.*
- *Time series analysis is introduces on pp8 without any indications for what it will be used. Add a sentence.*
- *“proving that a remarkable fraction of disinformation” – this is too strong. It may “imply” or “suggest” it.*
- *Pp 14, L 389-390. “because European elections are extremely un-polarised and not very interesting compared to national elections.” I would avoid this formulation (also, “extremely un-polarised” is a terrible phrasing.)*
- *Pp15 L.417: “****Relevant*** *spreaders of disinformation”. Why relevant? Choice of words.*
- *Pp 17 L 452: “a major issue with disinformation”. Rather a “strategy” than an “issue”?*

We followed the suggestions and changed the text accordingly.
