# Supplementary material for: Investigating Italian disinformation spreading on Twitter in the context of 2019 European elections
Source: PLoS One. 2020 Jan 17;15(1):e0227821. doi: 10.1371/journal.pone.0227821 (PMC6968875; doi:10.1371/journal.pone.0227821)
Supplement: Attachment — Submitted filename: response_reviewers_final_final.docx [file pone.0227821.s006.docx]

**Manuscript PONE-D-19-20362**

**Investigating Italian disinformation spreading on Twitter in the context of 2019 European elections**

# Response to Reviewers

## We appreciate the interest that the Editor and the Reviewers have shown in our manuscript and the constructive criticism they have given. We addressed the concerns of all Reviewers, which you will find below in a point-by-point response. Correspondingly, we revised several parts of the manuscript (the new or modified text is in blue in the revised manuscript).

## In summary:

## We added a reference for the topic analysis and provided a statistical validation for connections in the network of websites; we also discussed in detail the observation on the Twitter Streaming API and addressed the issue with influential targets of misinformation by re-sizing the corresponding section.

## We revised carefully the text in order to avoid inconsistencies and improve the clarity of the manuscript.

## We are grateful to the Reviewers for their comments and suggestions, which contributed to improve clarity and completeness of our manuscript.

**REVIEWER 2**

*The authors improved the paper and (shortly) addressed my concerns. Reading again the paper I noted very few points that captured my attention and that need further clarification:*

- *One of the main findings of the article is the evidence of the interconnections among different outlets across the world with evident connections between Italy and Russia. Every analysis in the paper has been subject to statistical validation except this one. I think the author should perform a statistical validation of such evidence. For instance, they could run several instances of a rewiring algorithm (degree preserving or strength preserving or both) over the network of Fig 11B to test if the percentage of links across countries in higher/lower than expected.*

We followed the suggestion and added a paragraph in that sense (lines 612-620).

- *Figure 1: the authors should specify what they mean by network failure.*

We added a comment in that sense in the caption.

- *Figure 5: what are the clearer bars on top of the darker bars? Perhaps confidence intervals? The authors should clarify this aspect.*

They correspond to the fraction of tweets published by recently created users as already specified in the caption:

“Fractions of users created in the six months before the elections are indicated with **lighter shades**; these account respectively for 0.18% (Rare), 0.6% (Low), 2.04% (Medium) and 2.98% (High) of total tweets”

- *A paragraph is titled ”Dismantling the disinformation network”. In the paper there are two networks the Twitter diffusion network and the network of websites. The disinformation network (according to what I understood and guessing from the analysis they carried on) should be the Twitter diffusion network but I am not sure. The authors should be clear in this regard.*

We made the analysis clearer by modifying the title in “Dismantling the disinformation network on Twitter”.

*As a final remark I suggest the authors to be very specific and precise when referring to or when interlinking the different elements of the paper. Indeed, they carry on a lot of different analyses on different networks and the feeling of the reader is to be, from time to time, a bit lost.*

**REVIEWER 3**

*I'm pretty much fine with the current version, but I’ve made comments handwriting in the document (second half of the pdf.) See attached. (And sorry for my handwriting. :)*

We are very thankful for carefully reviewing the entire text. *-- I checked Shao et al 2008 for any mention of the claim that 100% of tweets would be captured, and found nothing. What page/line are you referring to? I then looked at Twitter API documentation on how much the free streaming API captures, and only came up with something like 1%. There’s no mention that this would be different just because you’re tracking URLs. There’s also plenty of literature on the limitations of using the Free Streaming API, for instance that the selection of tweets is not a random sample. I would like a reference to the documentation where it’s stated that this in fact captures 100% of tweets. Or if it in fact does not capture 100%, a mention of this issue in the limitation section. (If it does not capture 100% of tweets, this likely impacts other parts of the text making claims of the prevalence of misinformation tweets.)*

Indeed, we built our work based on Hoaxy framework and the contributions that resulted from the tool, namely Refs 7-8-27-28.

The “completeness argument” is provided in:

**Ref 7 (former Ref’8):**

(Introduction, page 3)

*“Hoaxy retrieves the full and comprehensive set of tweets that share (i.e., include a link to)*

*articles and fact-checks. These tweets are important because, by tracking them, we can observe how a particular piece of content spreads over the social network. It is important to note that Hoaxy collects 100% of these tweets, not a sample.”*

(Discussion, page 19)

“*Recall that Hoaxy collects 100% ofthe tweets carrying each piece ofmisinformation in our collection, not a sample. As a result, our analysis provides a complete picture ofthe anatomy of the misinformation network.”*

**Ref 28 (Introduction, page 1):**

*“Note that the particular Twitter API endpoint we used for the collection enables Hoaxy to obtain not a sample stream, but a complete set of tweets that link to our target domains.”*

Whereas in **Ref’27** there is no explicit mention to the issue:

**(System Architecture, page 2):**

*“For example, we use the Twitter streaming API to do real-time tracking of news sharing. Because tweets are limited to 140 characters, the most common method to share a news story on Twitter is to include directly a link to its Web article. This means that we can focus only on tweets containing links to specific domains (websites), a task that is performed efficiently by the filter endpoint of the Twitter streaming API.”*

We actually talked in the past to developers of Hoaxy who told us what follows:

*“*The streaming API already returns 100% of the tweets that match the keywords. *The only caveat is that in this way you can collect up to 1% of the total volume of the stream for that day. So when you select domains of italian websites, make sure that the overall traffic does not exceed that amount. Keep in mind that these days there are roughly 300M tweets / day. So you have to make sure you don't track domains that generate more than 3M tweets / day. If you go beyond that, you may not get all the tweets (you will receive quota limit messages if that happens).* ***This is not documented anywhere on the official documentation of Twitter. We discovered it on our own and was confirmed to us by multiple people.****”*

We believe that the lack of documentation is probably the reason why they never mentioned such “caveat” in above mentioned contributions (which we repetitiously mention throughout the manuscript, see for instance lines 95,131,135).

*-- The method of the theme/topic modeling is still a bit unclear to me. E.g. how are the list of topic words summed up? Adding a method reference to someone else using the same method would be good.*

We added a reference (Ref’47) for dictionary-based text-analysis in the first paragraph and specified next that we counted keywords appearing in titles as to compute the relative presence of each theme (see lines 379-383, 396-397).

*-- The "targets of misinformation" section method is now clarified as "we recognized a few influential users". Is this really the method? I'm all for qualitative approaches, but just looking for things that you recognize seems a bit strong? Also, is the section really necessary - I'm not sure what it adds?*

We reformulated that part of the manuscript (see lines 492-494) which now is less emphasized: as a matter of fact, the three examples as qualitatively recognized (just like Matteo Salvini leader of the “Lega” party) and the “influence” is however related to their presence in Top-10 rankings. We believe that this small paragraph is necessary to show that the centrality in the network does not necessarily mean an active role in the spreading, just like we observed with Matteo Salvini who is “manipulated” to deceive other users.

*-- The new text needs to be checked for spelling and grammar. See comments in pdf.
-- Check reference list. Some entries lack year/journal/etc.
-- Check that terminology is consistent: now it uses theme/topic, fake/false/junk news, etc.
-- The pp22 section is still called "coordinated strategies of deception" even though the rest of the text is changed. Do change this.*

We revised the text accordingly (see lines 64,366, 371-373, 559, 599 and reference list).
