# Supplementary material for: Investigating Italian disinformation spreading on Twitter in the context of 2019 European elections
Source: PLoS One. 2020 Jan 17;15(1):e0227821. doi: 10.1371/journal.pone.0227821 (PMC6968875; doi:10.1371/journal.pone.0227821)
Supplement: Attachment — Submitted filename: rebuttal_letter.docx [file pone.0227821.s007.docx]

**Manuscript PONE-D-19-20362**

**Investigating Italian disinformation spreading on Twitter in the context of 2019 European elections**

# Response to the Editor

## We appreciate the interest that the Editor and the Reviewers have shown in our manuscript and the constructive criticism they have given, and we are glad of the positive judgement of both Reviewers and the Editor. We addressed the final concerns of the Editor regarding the limitations of our work by adding a new related sub-section in the “Methods” named “Limitations” (the new or modified text is in blue in the revised manuscript). We summarize below these limitations:

*The reviewers are satisfied with your revision, however the manuscript is still lacking a dedicated Limitations section, as requested in previous revisions. I would suggest the authors to use this section to discuss all limitations emerged during the review process.*

We thank the editor for suggesting this additional (sub)section. We expanded on the following limitations:

1. We remark that we focus only on Twitter which has limited usage for news consumption in Italy compared to other platforms such as Facebook and Whatsapp, which should be investigated further if only data was available (as mentioned in the Introduction and reiterated in the Conclusions)
2. We comment on the usage of Twitter Streaming API and the 1% rate limit which might apply (as highlighted by Reviewer#1 and Reviewer#3 in several reviews); we found a reference in past literature (ref’42) of such behavior but there is no mention of this in the current Twitter documentation. Authors of refs’7-8-27, who inspired our work and who used the same data collection approach, confirmed that this limitation is most likely to be still present; however, as the daily volume of tweets exceed 200 million tweets per day (cf. <https://blog.twitter.com/official/en_us/a/2011/200-million-tweets-per-day.html>) and we only collect 2-3k tweets, our collection is not hindered by such limitation (and thus neither our final results).
3. We remark that we only monitor disinformation which takes place as news articles shared on Twitter via URLs and that originate from a limited set of sources; we are missing the “big picture” relative to photos and videos that circulate on-line, and we do not verify the veracity of any of our news items. Though, we believe that we are drawing a consistent picture of Italian disinformation on Twitter, in line with the methodology of ref’7-8-27.
4. We stress the fact that when we observe hyperlinks from Italian sources to other disinformation websites, we are just showing that outlets sharing disinformation news often refer to similar sources and tend to deliver similar stories; we cannot prove actual coordination between different outlets and countries (although Avaaz report suggested it, ref’26), as remarked by both the Editor and Reviewer #3.

*I would also invite the authors to work more to smooth their claims, especially about the Russian influence.*

We thank the Editor for raising this concern; as a matter of fact, we already rephrased in previous revisions several of our claims in a more neutral way thanks to suggestions of Reviewer#3. In the revised manuscript, we have just 6 references to “Russia”, cf. lines 18, 41, 606, 607 and Fig. 11, and in all these cases we do only observe the presence of hyper-links to Russian websites, without making particular claims. Nonetheless, we additionally re-phrased a paragraph in both the Abstract and the Introduction (see lines 17-19, 121-122) and added a specific mention to connections between websites in the Limitations section (see lines 282-287).
